# Magnesium Supplementation Modifies Arthritis Synovial and Splenic Transcriptomic Signatures Including Ferroptosis and Cell Senescence Biological Pathways

**DOI:** 10.3390/nu16234247

**Published:** 2024-12-09

**Authors:** Teresina Laragione, Carolyn Harris, Pércio S. Gulko

**Affiliations:** Division of Rheumatology, Department of Medicine, Icahn School of Medicine at Mount Sinai, New York, NY 10029, USA

**Keywords:** arthritis, inflammation, autoimmune, diet, senescence

## Abstract

Background: Rheumatoid arthritis (RA) is a common systemic autoimmune inflammatory disease that can cause joint damage. We have recently reported that oral magnesium supplementation significantly reduces disease severity and joint damage in models of RA. Methods: In the present study, we analyzed the transcriptome of spleens and synovial tissues obtained from mice with KRN serum-induced arthritis (KSIA) consuming either a high Mg supplemented diet (Mg2800; n = 7) or a normal diet (Mg500; n = 7). Tissues were collected at the end of a 15-day KSIA experiment. RNA was extracted and used for sequencing and analyses. Results: There was an enrichment of differentially expressed genes (DEGs) belonging to Reactome and Gene Ontology (GO) pathways implicated in RA pathogenesis such as RHO GTPases, the RUNX1 pathway, oxidative stress-induced senescence, and the senescence-associated secretory phenotype. Actc1 and Nr4a3 were among the genes with the highest expression, while Krt79 and Ffar2 were among the genes with the lowest expression in synovial tissues of the Mg2800 group compared with the Mg500 group. Spleens had an enrichment for the metabolism of folate and pterines and the HSP90 chaperone cycle for the steroid hormone receptor. Conclusions: We describe the tissue transcriptomic consequences of arthritis-protecting Mg supplementation in KSIA mice. These results show that oral Mg supplementation may interfere with the response to oxidative stress and senescence and other processes known to participate in RA pathogenesis. We provide new evidence supporting the disease-suppressing effect of increased Mg intake in arthritis and its potential to become a new addition to the therapeutic options for RA and other autoimmune and inflammatory diseases.

## 1. Introduction

Rheumatoid arthritis (RA) affects nearly 1% of the population and is associated with increased risk of disability and reduced longevity [1]. RA has both genetic and non-genetic risk factors. While knowledge about genetic susceptibility has expanded [2], little is known about environmental and dietary contributions to this disease [3]. RA disease remission remains uncommon [4]; therefore, identifying and modifying environmental and dietary risk factors has the potential to further help understand disease susceptibility and improve disease control.

Magnesium (Mg) is the second most abundant intracellular cation in the human body and is involved in several biochemical functions including enzymatic activity and gene transcription [5]. In vitro studies show that increased concentrations of Mg reduce LPS-induced levels of pro-inflammatory cytokines such as TNFα, IL-6, and IL-8 and suppress NFκB activation in cultured macrophages or placental explants [6,7]. Levels of TNFα, IL-6, IL-8, and NFκB activity are increased in RA and have been associated with joint inflammation and implicated in synovial hyperplasia and joint damage [1,8]. Furthermore, Mg is typically deficient in the US diet, with nearly 40% of the population consuming less than the required amount [9]. Therefore, we considered that the commonly Mg-deficient US diet might favor pro-inflammatory pathways contributing to RA susceptibility and/or disease severity. We hypothesized that the administration of Mg might be beneficial in the treatment of RA and rodent models of RA. We tested this hypothesis in mouse models of RA induced with the KRN serum transfer and in collagen-induced arthritis and showed that the mice receiving a high Mg diet were significantly protected, developing lower arthritis severity scores and preserving a nearly normal joint architecture without erosive changes [10]. Arthritis protection in the high Mg diet was also associated with decreased expression of the pro-inflammatory cytokines TNFα, IL1β, and IL6 and increased numbers of IL10-producing Tr1 and Foxp3+ Treg cells [10]. The Mg diet’s protective effect was microbiome dependent [10].

However, it was not clear how the high Mg diet affected gene expression in tissues, particularly the synovial tissues. In the present study, we describe the transcriptomic changes induced by an arthritis-protecting high Mg diet in synovial tissues and spleens from arthritic mice and identify new pathways and genes relevant to disease pathogenesis that are regulated by Mg supplementation.

Our discoveries raise the possibility that oral Mg treatment has the potential to become a new, inexpensive, and benign treatment for RA and perhaps for other inflammatory and autoimmune diseases as well.

## 2. Materials and Methods

**Mice.** Male C57BL/6 mice were purchased from Taconic (Rensselaer, NY, USA). Male NOD/ShiLtJ were purchased from Jackson Laboratories (Farmington, CT, USA). KBxN (KRN) TCR transgenic mice (gift from Dr. C. Benoist, Boston, MA, USA) were bred and maintained at Mount Sinai. All mice were housed under specific pathogen-free conditions and a 12 h light–dark cycle. All experiments were conducted under protocol number 2014-0283 approved by the Mount Sinai Institutional Animal Care and Use Committee.

**KRN serum-induced arthritis (KSIA).** KRN TCR transgenic mice were crossed with NOD (KRN x NOD F1) and the arthritogenic serum was collected from 60-day-old arthritic mice. Serum from different batches was pooled and administered to male C57BL/6 mice at 100 µL IP on days 0 and 2. Mice typically developed arthritis on day 3 and were followed for 15 days and scored three times a week [11,12]. Mice used in the arthritis and diet experiments were kept in the same room, shared the same rack, and were monitored daily. Analgesics or anti-inflammatory drugs were not used as they interfere with arthritis inflammation and immune responses.

**Arthritis activity and severity scoring.** The clinical arthritis score was determined according to a scoring scale ranging from 0 to 16 per mouse per day, as previously reported, where 1 = swelling and erythema in a single joint, 2 = swelling and erythema in more than one joint, 3 = swelling of the entire paw, and 4 = swelling of paw and inability to bear weight [12,13].

**Mg Dietary Regimens.** The diets were purchased from Teklad-Envigo Laboratories (Somerset, NJ, USA). Mice received identical diets, except for the amount of magnesium. Specifically, the diets were irradiated and had the following contents (g/kg): protein (17.7), carbohydrates (64.4), fat (6.2), casein (200), DL-methionine (3.0), sucrose (415), corn starch (250), soybean oil (60), cellulose (30), vitamin mix (Teklad 40060), ethoxyquin (antioxidant) (0.01), calcium phosphate, dibasic (13.7), potassium citrate (monohydrate) (7.7), calcium carbonate (4.8), sodium chloride (2.6), potassium sulfate (1.82), ferric citrate (0.25), manganous carbonate (0.12), zinc carbonate (0.056), chromium potassium sulfate (dodecahydrate) (0.02), cupric carbonate (0.012), potassium iodate (0.0004), and sodium selenite, (pentahydrate) (0.0004). The regular Mg diet had Mg oxide 0.822 g/kg of chow (Mg 500 ppm), and the high Mg diet had Mg oxide 2.3 g/kg of chow (Mg 2800 ppm).

**Figure 1:**
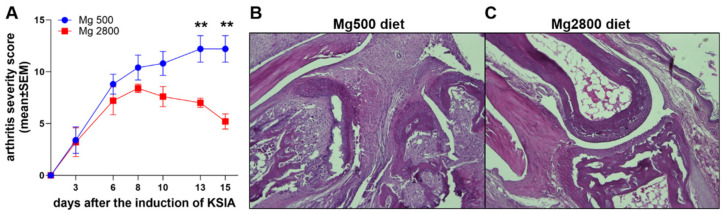
Arthritis severity scores of mice with KRN serum-induced arthritis (KSIA). (**A**) Mice were placed on either a normal Mg500 (n = 7) or a high Mg2800 (n = 7) diet 14 days prior to the induction of KSIA and kept on the same diet for an additional 15 days and scored for disease severity (** *p* = 0.004993 and *p* = 0.001462, respectively; non-paired *t*-test). Representative histology sections of KSIA mice on (**B**) the normal Mg500 diet, showing pronounced synovial hyperplasia and joint damage, and (**C**) the high Mg2800 diet, showing a protected and normal-looking joint without synovial hyperplasia or damage (H&E staining, 200× magnification).

Male C57BL/6 mice were fed either a normal Mg diet, Mg500, or a high Mg diet, Mg2800, for 14 days before the induction of KSIA. Following the induction of KSIA, the mice were kept on the same diet for an additional period of 15 days (Figure 1).

### 2.1. RNA Sequencing and Analyses

Synovial tissues and spleens from five different mice per Mg diet group were used for RNA extraction and sequencing. Total RNA was isolated from spleens and synovial tissues using an RNeasy Plus kit (Qiagen, Germantown, MD, USA) and quantified using a Nanodrop. Next, 400 ng of RNA per mouse was sent to Novogene (Beijing, China) for sequencing on Illumina platforms and analysis (Supplemental File S1). Briefly, differential expression analysis of two conditions/groups (five biological replicates per condition) was performed using the DESeq2 R package (1.20.0) [14]. The resulting *p*-values were adjusted using Benjamini and Hochberg’s approach for controlling the false discovery rate.

Gene Ontology (GO) enrichment analysis of differentially expressed genes was performed with the clusterProfiler R package. GO terms with a corrected *p*-value less than 0.05 were considered significantly enriched by differentially expressed genes. The Reactome database brings together the various reactions and biological pathways of human model species. Reactome pathways corrected *p*-value of less than 0.05 were considered significantly enriched by differentially expressed genes. ClusterProfiler software (version 3.2) was used to test the statistical enrichment of differentially expressed genes in the Reactome pathways (Supplemental File S1).

### 2.2. Statistics

Means were compared with the *t*-test or paired *t*-test and medians were compared with the rank-sum test whenever indicated using GraphPad Prism 6 (San Diego, CA, USA).

## 3. Results

**The Mg2800 diet significantly protects mice with KSIA.** C57BL/6 mice were placed on either the normal Mg500 or high Mg2800 diet prior to the induction of KSIA and kept on the same diet for an additional period of 15 days (n = 7 per diet group). Mice on the Mg2800 diet were protected and had lower arthritis severity scores that reached statistical significance on days 13 and 15 (*p* = 0.004993 and *p* = 0.001462, respectively; non-paired *t*-test; Figure 1).

**Figure 2:**
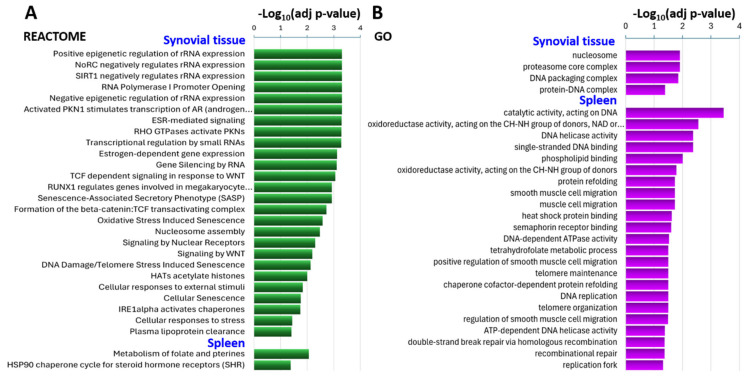
Biological pathways enriched in the DEGs between KSIA arthritic mice on Mg2800 and Mg500 diets. (**A**) Selected Reactome biological pathways and cellular processes enriched in synovial tissues (top section) and spleens (bottom section). (**B**) Selected Gene Ontology (GO) pathways enriched in the DEGs between mice on Mg2800 and Mg500 diets in the synovial tissues (top section), and spleens (bottom section). (See Supplemental Tables S1 and S2 for additional details).

**Synovial tissue enrichment for Reactome and GO pathways implicated in RA pathogenesis, including senescence.** There was a predominance of DEGs with reduced expression levels in the Mg2800 synovial tissues compared with the Mg500 group. There was synovial tissue enrichment for genes involved in several Reactome pathways implicated in gene transcription and gene regulation such as epigenetic regulation, RNA polymerase activity, transcriptional regulation, nuclear receptor transcription (androgen and estrogen receptor dependent), and gene silencing by small RNAs (Figure 2A, Supplemental Table S1).

Reactome processes implicated in RA pathogenesis such as the RUNX1 pathway, the formation of the beta-catenin:TCF transactivating complex, RHO GTPases activating PKNs, and signaling by WNT were also enriched among the DEGs in synovial tissues (Figure 2A, Supplemental Table S1).

There was an enrichment of genes in the Reactome pathways senescence-associated secretory phenotype (SASP), oxidative stress-induced senescence, cellular senescence, and DNA damage/telomere stress-induced senescence (Figure 2A, Supplemental Table S1).

There were four GO pathways enriched among the DEGs in synovial tissues, specifically the nucleosome, proteasome core complex, DNA packaging complex, and protein–DNA complex (Figure 2B, Supplemental Table S2).

**Figure 3:**
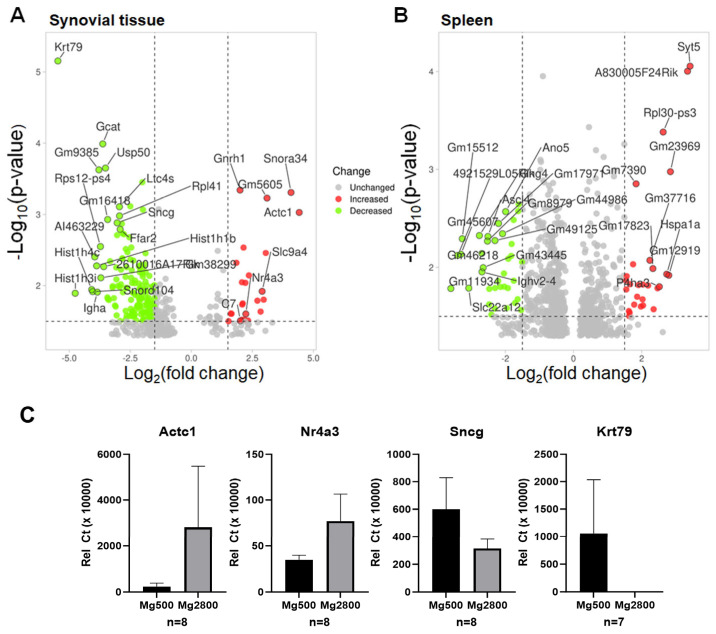
Volcano plots of the DEGs between KSIA arthritic mice on the Mg2800 diet and those on the Mg500 diet and selected genes’ qPCR confirmation. (**A**) Volcano plot of DEGs in synovial tissues. (**B**) Volcano plot of DEGs in spleens. (**C**) Quantitative PCR (qPCR) confirmation of selected genes expressed in increased and decreased levels in the Mg2700 diet synovial tissues showing a trend in the same direction as seen in the RNA sequencing analyses (*p* > 0.05).

The genes with the most significantly increased expression in the synovial tissues of the arthritis-protected Mg2800 diet group included Snora34, Gnrh1, Actc1, Nr4a3, and Slc9a4 (Figure 3A, Supplemental Table S3). The genes with the most significantly decreased expression in synovial tissues of the Mg2800 diet group, compared with the Mg500 diet group, included Krt79, Pth1r, Gcat, Ffar2, and Sncg (Figure 3A, Supplemental Table S3). Also, among the genes with the most significantly reduced expression in the Mg2800 diet group were neutrophil genes S100a13, S100b, Elane, and MPO (Supplemental Table S3), likely reflecting decreased neutrophil influx into the synovial tissues.

Two of the genes expressed in increased levels (Actc1 and Nr4a3) and two of the genes expressed in lower levels (Sncg and Krt79) in synovial tissues from mice in the Mg2800 diet group, compared with the Mg500 diet group, were further confirmed with qPCR.

**Gene enrichment for Reactome and GO pathways in the spleens.** Two Reactome pathways, the metabolism of folate and pterines, and the HSP90 chaperone cycle for steroid hormone receptors were enriched among the splenic DEGs (Figure 2A, Supplemental Table S1). GO pathway-enriched DEGs in the spleens included protein refolding, cell migration (smooth muscle and muscle), heat shock protein binding, and semaphorin receptor binding (Figure 2B, Supplemental Table S2). As seen in synovial tissues, there was also enrichment for pathways involved in DNA stability and replication and senescence, including DNA helicase activity, telomere maintenance, oxidoreductase activity, and others (Figure 2B, Supplemental Table S2).

The genes with the most significantly increased expression in spleens from the Mg2800 diet group included Syt5 and Hspa1a (Figure 3B, Supplemental Table S4). The genes with the most significantly decreased expression in spleens from the Mg2800 diet group included Ano5 and Gng4, two genes (Figure 3B, Supplemental Table S4). To our knowledge, none of these genes has previously been implicated in autoimmune or inflammatory diseases.

## 4. Discussion

RA is a common chronic autoimmune and inflammatory disease that can be debilitating and cause disability. While there are strong genetic and non-genetic components in the regulation of susceptibility and severity [15,16], very little is known about non-genetic environmental and dietary factors [3]. Smoking has been strongly associated with RA, but only a few other environmental or dietary factors have been reproducibly implicated in disease susceptibility or severity [15]. Mg is typically deficient in the US diet with nearly 40% of the population consuming less than the required amounts [9]. Similarly, there is also evidence that RA patients have a diet deficient in Mg [17], raising the possibility that it might be a dietary risk factor for disease.

We have recently demonstrated that increasing the dietary intake of Mg has a significant protective effect in mouse models of RA [10]. The high Mg2800 diet reduced arthritis severity and joint damage and reduced synovial inflammation and the expression of cytokines while increasing the numbers of CD4+Foxp3+ Treg cells and IL10-producing Tr1 cells [10]. We demonstrated that this arthritis-suppressing effect was highly dependent on the intestinal microbiome [10]. In the present study, we describe for the first time the transcriptomic changes induced by the increased dietary intake of Mg (Mg2800) in the synovial tissues and spleens of arthritic mice.

Mg is required for several cellular and enzymatic processes, including gene transcription, energy metabolism, and others [5], and has also been implicated in epigenetic regulation [18,19], including gene methylation [20]. Among the most significantly enriched pathways and processes we detected in the synovial tissues were those implicated in the epigenetic regulation of gene expression, including “SIRT1 negatively regulates rRNA expression”, “nucleosome”, and “RUNX1 regulates genes involved in megakaryocyte differentiation and platelet function.” RUNX1 is associated with the susceptibility risk for RA [21,22] and was recently shown to epigenetically regulate gene expression, contributing to reduced disease severity in autoimmune arthritis [23]. Epigenetic regulation and epigenetic abnormalities have been described in the synovial tissues and implicated in RA pathogenesis [24,25,26,27,28]. Oral supplementation of Mg significantly reduces the expression of inflammatory genes in overweight patients [29] and in experimental autoimmune arthritis [10], and our results suggest that this effect may be in part epigenetically regulated by Mg.

“Senescence” and “oxidative stress-induced senescence”, as well as “telomerase maintenance”, were also among the most significantly enriched pathways in the synovial tissues. Mg deficiency has been shown to accelerate cellular senescence and telomerase attrition [30,31], and Mg supplementation can affect senescence via interference with oxidative stress [32]. Cellular senescence has been implicated in the pathogenesis of RA and other autoimmune diseases [33,34], and interfering with senescence has the potential to improve disease control [35,36,37]. Taken together, our results suggest that Mg supplementation ameliorates arthritis in part via changes in cellular senescence pathways implicated in arthritis and other chronic diseases.

Other RA-associated pathways enriched in the synovial tissues’ DEGs included “beta-catenin:TCF transactivating complex”, “signaling by WNT”, and “RHO GTPases activate PKNs”. Beta-catenins and WNT pathway genes are expressed by the synovial tissues [38,39] and have been implicated in joint damage [40], including the activated and pro-inflammatory behavior of the RA synovial fibroblast [41,42]. RHO GTPases RHOA, RHOB, RHOC, and RAC1 bind PKN1, PKN2, and PKN3 [43,44,45,46,47]. Of these RHO GTPases, RHOA and RAC1 regulate synovial fibroblast behavior and arthritis severity and joint damage in autoimmune arthritis [48,49,50,51]. These findings suggest new mechanisms of action for oral Mg supplementation in arthritis by interfering with the expression of genes known to regulate RA pathogenesis and joint damage.

The genes with the most significantly increased expression in the synovial tissues of the arthritis-protected Mg2800 diet group included Actc1 (actin alpha cardiac muscle 1) and Nr4a3 (nuclear receptor subfamily 4 group A member 3) (Figure 3A, Supplemental Table S3). Actc1 is involved in cell motility, muscle regeneration [52], and ferroptosis [53]. It is not only associated with poor prognosis in glioblastomas [54] but also metastasis-free survival in prostate cancer [55], and low levels are associated with aging [56], suggesting anti-aging or anti-senescence activity, which in the present study was induced by Mg supplementation.

Nr4a3 encodes a member of the steroid–thyroid hormone–retinoid receptor superfamily and can dimerize with retinoid X receptors (RXR) [57]. NR4a3 has anti-oxidative activity in glioblastoma cells [58] and has been suggested to have tumor-suppressive activity [59]. These functions would be beneficial in the arthritic synovial tissues in reducing oxidative damage, with them having potential anti-inflammatory activity, particularly when dimerizing with RXR, and reducing synovial hyperplasia.

The genes with the most significantly decreased expression in synovial tissues of the Mg2800 diet group, compared with the Mg500 diet group, included Krt79 (keratin 79), Ffar2 (free fatty acid receptor 2, or Gpr43), and Sncg (synuclein gamma) (Figure 3A, Supplemental Table S3). Also, among the genes with the most significantly reduced expression in the Mg2800 diet group were neutrophil genes S100a13, S100b, Elane, and MPO (Supplemental Table S3).

Krt79 is expressed in the skin and fat and by granulocytes, macrophages, and T cells (The Human Protein Atlas, www.proteinatlas.org). Krt79 is expressed by some cancers such as leukemias and sarcomas (www.proteinatlas.org) and may be involved in the regulation of immune responses [60]. Its reduced expression in synovial tissues from arthritis-protected mice on the Mg2800 diet could represent decreased leukocyte tissue infiltration, including reduced numbers of granulocytes, and it will be interesting to examine its role in inflammation and the parallel between its role in sarcoma growth and invasion and in synovial tissues and synovial fibroblasts.

Ffar2 encodes a G protein-coupled receptor for short-chain free fatty acids and may be involved in the inflammatory response and ferroptosis [61]. Ffar2 regulates metabolite sensing by colonic innate lymphoid cells [62], and its loss exacerbates colonic inflammation [63] and promotes colon cancer [64]. Ffar2 is expressed by chondrocytes, where it can suppress inflammation induced by IL-1β and inhibit NFkB activation [65]. In RA synovial fibroblasts, Ffar2 activation suppresses TNFα-induced inflammatory responses such as IL-1β, chemokines and ROS production, and NFkB activation [66]. Ffr2 can also activate neutrophils via acetoacetate, a cell central to KSIA and RA [67]. Therefore, given that most of the literature suggests anti-inflammatory activity for Ffar2, we consider that its reduced expression in synovial tissues from the protected mice on the Mg2800 diet represented, along with the reduced expression of S100a13, S100b, Elane, and MPO, decreased neutrophil infiltration in the tissues.

Sncg has been implicated in cancer metastasis via the MAPK pathway [68] and also in cancer cell migration [69]. Sncg is also involved in the regulation of cell senescence [70], giving rise to yet another senescence pathway gene regulated by Mg that was identified in the present study.

We also analyzed the spleens as peripheral lymphoid tissues representative of the systemic effects of the different Mg diets on different cells that may or may not be present in the synovial tissues. The genes with the most significantly increased expression in spleens from the Mg2800 diet group included Hspa1a (heat shock protein family A, member 1A) and Syt5 (syaptotagmin 5). Hspa1a stabilizes existing proteins to prevent aggregation [71] and is involved in the ubiquitin–proteasome pathway [72]. Increased expression of Hspa1a can also protect cells from thermal [73] and oxidative damage [74]. Recently, levels of Hspa1a protein in the synovial tissues of RA, OA, and calcium pyrophosphate disease were associated with less severe histology scores [75], further suggesting a potentially protective effect. While the present study did not examine levels of protein, our observations raise the possibility that Mg supplementation may be a new option to increase levels of this potentially protective gene.

Little is known about the exocytosis regulator gene Syt5, but its expression is associated with increased survival in renal carcinoma [76] and glioblastomas [77]. Our observations show that Mg supplementation increases the levels of Syt5, and while the precise mechanism of action of this gene in arthritis and cancer remains unknown, increasing dietary Mg may be an option worth considering in future trials in RA and cancer.

The genes with the most significantly decreased expression in spleens from the Mg2800 diet group included Ano5 (anoctamin 5) [78] and Gng4 (G protein subunit gamma 4) [79,80], and both are associated with worse cancer outcomes and favor proliferation, migration, and invasion. Therefore, the reduced expression of Ano5 and Gng4 may be beneficial in reducing lymphocytes’ and other immune cells’ migration into the synovial tissues.

Lastly, another gene among the most significantly decreased expression genes in the Mg2800 diet group was Ascl4 (achaete-scute family bHLH transcription factor 4). Ascl4 is required for ferroptosis [81], a process implicated in RA pathogenesis [82,83]. Therefore, the Mg-induced reduced expression of Ascl4 may reduce ferroptosis and contribute to a protective effect in arthritis.

Several of the most significant DEGs were involved in ferroptosis, which is a newly discovered form of cell-regulated death characterized by iron-dependent lipid peroxidation [82,84]. Ferroptosis has been implicated in the pathogenesis of RA and rodent models of RA [82,83,85], cancer [53,55], metabolic dysfunction-associated steatohepatitis (MASH) [86], and other forms of liver disease, including liver fibrosis [86,87,88]. Not all Mg2800-induced ferroptosis DEGs were expressed in the same orientation and not all of them will necessarily have a major role in synovial ferroptosis. Nevertheless, our observations suggest that one of the mechanisms of the arthritis-improving effect of oral Mg supplementation may be related to regulating ferroptosis. Interestingly, magnesium isoglycyrrhizinate, which is a magnesium salt, inhibits ferroptosis and prevents experimental liver fibrosis [89,90], supporting our data.

## 5. Conclusions

In conclusion, we describe for the first time the transcriptomic changes caused by an arthritis-protective high Mg2800 diet. In the spleens and synovial tissues of the Mg2800 diet group, there was an enrichment of several biological pathways implicated in RA pathogenesis such as RHO GTPases, epigenomic regulation of genes, senescence, and ferroptosis. The most significantly expressed DEGs were also involved in some of these processes, particularly cell senescence and ferroptosis, and some cancer-associated genes. Our findings provide additional evidence supporting the multiple processes affected by Mg that have the potential to be beneficial for RA patients and perhaps other inflammatory diseases, which are safe and low cost and require testing in humans.

## Data Availability

All data are available in the main text or the Supplementary Materials, and the RNA sequences have been deposited in the GEO NCBI repository under accession number GSE276773.

## Supplementary Materials

The following supporting information can be downloaded at: https://www.mdpi.com/article/10.3390/nu16234247/s1. File S1: Supplemental methods and details about RNA sequencing analyses; Table S1: Reactome Pathways and processes enriched in differentially expressed genes in the synovial tissues and spleens; Table S2: Gene Ontology GO enriched pathways in Mg2800 vs Mg500 synovial tissues and spleens; Table S3: DEG in the synovial tissues of mice on the Mg2800 versus Mg500 diets. Table S4: DEG in the spleens of mice on the Mg2800 versus Mg500 diets.
