# Supplementary material for: Magnesium Supplementation Modifies Arthritis Synovial and Splenic Transcriptomic Signatures Including Ferroptosis and Cell Senescence Biological Pathways
Source: Nutrients. 2024 Dec 9;16(23):4247. doi: 10.3390/nu16234247 (PMC11644223; doi:10.3390/nu16234247)
Supplement: Supplementary file 1 [file nutrients-16-04247-s001.zip › Supplementary Table S1_ Reactome enriched pathways in Mg2800 vs Mg500_synovial tissues and spleens.pdf]

Table S1. Reactome Pathways and processes enriched in differentially expressed genes in the synovial tissues.

ReactomeI	Description	GeneRatio	BgRatio	pvalue	padj	-log10(adj pvalue)	geneID	geneName	EntrezID	Count	down	up
	**SYNOVIAL TISSUES**											
R-MMU-52	Positive epigenetic regulation of rRNA expression	10/165	75/8206	2.21E-06	0.000495	3.305426841	ENSMUSG(	Hist1h3e/Hist1h2bb/Hist1h2ad/Hist2h	319151/31	10	10	0
R-MMU-52	B-WICH complex positively regulates rRNA expression	10/165	75/8206	2.21E-06	0.000495	3.305426841	ENSMUSG(	Hist1h3e/Hist1h2bb/Hist1h2ad/Hist2h	319151/31	10	10	0
R-MMU-42	NoRC negatively regulates rRNA expression	8/165	46/8206	3.07E-06	0.000495	3.305426841	ENSMUSG(	Hist1h3e/Hist1h2bb/Hist1h2ad/Hist2h	319151/31	8	8	0
R-MMU-42	SIRT1 negatively regulates rRNA expression	8/165	48/8206	4.29E-06	0.000495	3.305426841	ENSMUSG(	Hist1h3e/Hist1h2bb/Hist1h2ad/Hist2h	319151/31	8	8	0
R-MMU-73	RNA Polymerase I Promoter Opening	8/165	48/8206	4.29E-06	0.000495	3.305426841	ENSMUSG(	Hist1h3e/Hist1h2bb/Hist1h2ad/Hist2h	319151/31	8	8	0
R-MMU-52	Negative epigenetic regulation of rRNA expression	8/165	49/8206	5.04E-06	0.000495	3.305426841	ENSMUSG(	Hist1h3e/Hist1h2bb/Hist1h2ad/Hist2h	319151/31	8	8	0
R-MMU-56	Activated PKN1 stimulates transcription of AR (androgen receptor) regu	8/165	50/8206	5.90E-06	0.000497	3.304072109	ENSMUSG(	Hist1h3e/Hist1h2bb/Hist1h2ad/Hist2h	319151/31	8	8	0
R-MMU-89	ESR-mediated signaling	11/165	104/8206	6.90E-06	0.000508	3.29385569	ENSMUSG(	Hist1h3e/Hist1h2bb/Hist1h2ad/Hist2h	319151/31	11	11	0
R-MMU-56	RHO GTPases activate PKNs	9/165	69/8206	8.71E-06	0.000515	3.288045895	ENSMUSG(	Hist1h3e/Hist1h2bb/Hist1h2ad/Hist2h	319151/31	9	9	0
R-MMU-92	Nonsense-Mediated Decay (NMD)	10/165	88/8206	9.62E-06	0.000515	3.288045895	ENSMUSG(	Rpl41/Rps27a/Rpl27/LOC108167922/R	67945/782	10	8	0
R-MMU-97	Nonsense Mediated Decay (NMD) enhanced by the Exon Junction Comp	10/165	88/8206	9.62E-06	0.000515	3.288045895	ENSMUSG(	Rpl41/Rps27a/Rpl27/LOC108167922/R	67945/782	10	8	0
R-MMU-55	Transcriptional regulation by small RNAs	10/165	89/8206	1.07E-05	0.000523	3.281572369	ENSMUSG(	Hist1h3e/Hist1h2bb/Hist1h2ad/Hist2h	319151/31	10	10	0
R-MMU-73	RNA Polymerase I Promoter Clearance	10/165	90/8206	1.18E-05	0.000534	3.272682237	ENSMUSG(	Hist1h3e/Hist1h2bb/Hist1h2ad/Hist2h	319151/31	10	10	0
R-MMU-73	RNA Polymerase I Transcription	10/165	91/8206	1.30E-05	0.000547	3.261810283	ENSMUSG(	Hist1h3e/Hist1h2bb/Hist1h2ad/Hist2h	319151/31	10	10	0
R-MMU-22	Condensation of Prophase Chromosomes	8/165	56/8206	1.41E-05	0.000553	3.256995204	ENSMUSG(	Hist1h3e/Hist1h2bb/Hist1h2ad/Hist2h	319151/31	8	8	0
R-MMU-21	Epigenetic regulation of gene expression	10/165	93/8206	1.58E-05	0.000582	3.23542121	ENSMUSG(	Hist1h3e/Hist1h2bb/Hist1h2ad/Hist2h	319151/31	10	10	0
R-MMU-21	PRC2 methylates histones and DNA	8/165	58/8206	1.84E-05	0.000637	3.196038634	ENSMUSG(	Hist1h3e/Hist1h2bb/Hist1h2ad/Hist2h	319151/31	8	8	0
R-MMU-90	Estrogen-dependent gene expression	10/165	97/8206	2.29E-05	0.000751	3.124384082	ENSMUSG(	Hist1h3e/Hist1h2bb/Hist1h2ad/Hist2h	319151/31	10	10	0
R-MMU-21	Gene Silencing by RNA	10/165	98/8206	2.51E-05	0.000779	3.108617555	ENSMUSG(	Hist1h3e/Hist1h2bb/Hist1h2ad/Hist2h	319151/31	10	10	0
R-MMU-20	TCF dependent signaling in response to WNT	14/165	192/8206	2.99E-05	0.000881	3.054934261	ENSMUSG(	Hist1h3e/Wnt8b/Hist1h2bb/Hist1h2ad	319151/22	14	13	1
R-MMU-89	RUNX1 regulates genes involved in megakaryocyte differentiation and p	8/165	65/8206	4.29E-05	0.00117	2.931715666	ENSMUSG(	Hist1h3e/Hist1h2bb/Hist1h2ad/Hist2h	319151/31	8	8	0
R-MMU-25	Senescence-Associated Secretory Phenotype (SASP)	9/165	84/8206	4.37E-05	0.00117	2.931715666	ENSMUSG(	Hist1h3e/Hist1h2bb/Hist1h2ad/Hist2h	319151/31	9	9	0
R-MMU-20	Formation of the beta-catenin:TCF transactivating complex	8/165	70/8206	7.38E-05	0.001889	2.723747775	ENSMUSG(	Hist1h3e/Hist1h2bb/Hist1h2ad/Hist2h	319151/31	8	8	0
R-MMU-25	Oxidative Stress Induced Senescence	9/165	94/8206	0.000106	0.002611	2.583202245	ENSMUSG(	Hist1h3e/Hist1h2bb/Hist1h2ad/Hist2h	319151/31	9	9	0
R-MMU-60	Deposition of new CENPA-containing nucleosomes at the centromere	7/165	58/8206	0.000148	0.003348	2.475268723	ENSMUSG(	Hist1h2bb/Hist1h2ad/Hist2h2ac/H2afj,	319178/31	7	7	0
R-MMU-77	Nucleosome assembly	7/165	58/8206	0.000148	0.003348	2.475268723	ENSMUSG(	Hist1h2bb/Hist1h2ad/Hist2h2ac/H2afj,	319178/31	7	7	0
R-MMU-88	Transcriptional regulation by RUNX1	12/165	170/8206	0.000153	0.003348	2.475268723	ENSMUSG(	Hist1h3e/Hist1h2bb/Hist1h2ad/Hist2h	319151/31	12	12	0
R-MMU-32	RMTs methylate histone arginines	7/165	62/8206	0.000227	0.004777	2.320885584	ENSMUSG(	Hist1h3e/Hist1h2ad/Hist2h2ac/H2afj/H	319151/31	7	7	0
R-MMU-90	Signaling by Nuclear Receptors	11/165	153/8206	0.000247	0.00502	2.299273931	ENSMUSG(	Hist1h3e/Hist1h2bb/Hist1h2ad/Hist2h	319151/31	11	11	0
R-MMU-73	Chromosome Maintenance	8/165	84/8206	0.000268	0.005271	2.278127859	ENSMUSG(	Hist1h2bb/Hist1h2ad/Hist2h2ac/H2afj,	319178/31	8	8	0
R-MMU-19	Signaling by WNT	15/165	270/8206	0.000337	0.006401	2.19378232	ENSMUSG(	Hist1h3e/Wnt8b/Hist1h2bb/Hist1h2ad	319151/22	15	14	1
R-MMU-25	DNA Damage/Telomere Stress Induced Senescence	7/165	68/8206	0.000405	0.007448	2.127958278	ENSMUSG(	Hist1h2bb/Hist1h1b/Hist1h2ad/Hist2h	319178/56	7	7	0
R-MMU-68	Mitotic Prophase	9/165	114/8206	0.00046	0.008216	2.085345306	ENSMUSG(	Hist1h3e/Hist1h2bb/Hist1h2ad/Hist2h	319151/31	9	9	0
R-MMU-32	HATs acetylate histones	7/165	72/8206	0.000575	0.00996	2.001753389	ENSMUSG(	Hist1h3e/Hist1h2bb/Hist1h2ad/Hist2h	319151/31	7	7	0
R-MMU-38	XBP1(S) activates chaperone genes	3/165	Oct-06	0.000863	0.01453	1.83773201	ENSMUSG(	Sec61g/Gm4184/LOC102637269	20335/100	3	1	0
R-MMU-89	Cellular responses to external stimuli	19/165	427/8206	0.000904	0.014797	1.829820656	ENSMUSG(	Hist1h3e/Hist1h2bb/Mt3/Hist1h1b/His	319151/31	19	18	1
R-MMU-25	Cellular Senescence	10/165	155/8206	0.001102	0.01755	1.755715088	ENSMUSG(	Hist1h3e/Hist1h2bb/Hist1h1b/Hist1h2	319151/31	10	10	0
R-MMU-38	IRE1alpha activates chaperones	3/165	Nov-06	0.00117	0.018131	1.741583706	ENSMUSG(	Sec61g/Gm4184/LOC102637269	20335/100	3	1	0
R-MMU-89	Metabolism of RNA	20/165	478/8206	0.001408	0.021269	1.672251318	ENSMUSG(	Rpl41/Rps27a/Ppie/Rpl27/LOC108167	67945/782	20	18	0
R-MMU-56	UCH proteinases	7/165	88/8206	0.001895	0.027903	1.554342793	ENSMUSG(	Hist1h2ad/Hist2h2ac/Rps27a/Tfpt/Psn	319165/31	7	7	0
R-MMU-76	RNA Polymerase III Transcription Initiation From Type 3 Promoter	4/165	28/8206	0.002214	0.031806	1.497485602	ENSMUSG(	Polr3h/Polr2h/Polr2k/Snapc2	78929/245	4	4	0
R-MMU-22	Cellular responses to stress	16/165	364/8206	0.002622	0.036772	1.434482904	ENSMUSG(	Hist1h3e/Hist1h2bb/Hist1h1b/Hist1h2	319151/31	16	16	0
R-MMU-89	Plasma lipoprotein clearance	4/165	30/8206	0.002872	0.039345	1.405110582	ENSMUSG(	Lipc/Apoc1/Ap2s1/Npc2	15450/118	4	4	0
	**SPLEENS**											
R-MMU-19	Metabolism of folate and pterines	7/412	17/8026	1.11E-05	0.00867	2.061963243	ENSMUSG(	Aldh1l2/Mthfd1l/Dhfr/Mthfd1/Mthfr/I	216188/27	7	3	4
R-MMU-33	HSP90 chaperone cycle for steroid hormone receptors (SHR)	10/412	47/8026	0.000107	0.041645	1.380438847	ENSMUSG(	Tubb2b/Hsp90aa1/Stip1/Hspa1a/Hspa	73710/155	10	2	8
