# Supplementary material for: Magnesium Supplementation Modifies Arthritis Synovial and Splenic Transcriptomic Signatures Including Ferroptosis and Cell Senescence Biological Pathways
Source: Nutrients. 2024 Dec 9;16(23):4247. doi: 10.3390/nu16234247 (PMC11644223; doi:10.3390/nu16234247)
Supplement: Supplementary file 1 [file nutrients-16-04247-s001.zip › Supplementary Table S2. Gene Ontology enriched pathway in Mg2800 vs Mg500.pdf]

**Table S2.** Gene Ontology GO enriched pathways in Mg2800 vs Mg500_synovial tissues and spleens table

| GO Category | GOID | Description | GeneRatio | BgRatio | pvalue | padj | -log10(adj p | geneID | geneName | Count | Down | Up |
|---|---|---|---|---|---|---|---|---|---|---|---|---|
| | | **SYNOVIAL TISSUES** | | | | | | | | | | |
| CC | GO:000078 | nucleosome | 8/362 | 62/17361 | 4.19E-05 | 0.0123833 | 1.9071631 | ENSMUSG0 | Hist1h3e/Hi | 8 | 8 | 0 |
| CC | GO:000583 | proteasome core complex | 5/362 | 21/17361 | 5.93E-05 | 0.0123833 | 1.9071631 | ENSMUSG0 | Psmb3/Psm | 5 | 5 | 0 |
| CC | GO:004481 | DNA packaging complex | 8/362 | 70/17361 | 0.0001012 | 0.0140993 | 1.8508024 | ENSMUSG0 | Hist1h3e/Hi | 8 | 8 | 0 |
| CC | GO:003299 | protein-DNA complex | 11/362 | 154/17361 | 0.0003924 | 0.0410011 | 1.3872049 | ENSMUSG0 | Hist1h3e/Hi | 11 | 11 | 0 |
| | | **SPLEENS** | | | | | | | | | | |
| MF | GO:014009 | catalytic activity, acting on DNA | 26/790 | 181/16652 | 4.37E-07 | 0.000358 | 3.4460753 | ENSMUSG0 | Dntt/Isg20/ | 26 | 7 | 19 |
| MF | GO:001664 | oxidoreductase activity, acting on the CH-NH group of donors, NAD or NADP as acceptor | 7/790 | 17/16652 | 6.74E-06 | 0.002762 | 2.5587792 | ENSMUSG0 | Aldh1l2/Mt | 7 | 3 | 4 |
| MF | GO:000367 | DNA helicase activity | 10/790 | 42/16652 | 2.01E-05 | 0.0042313 | 2.3735221 | ENSMUSG0 | Cdc45/Twnl | 10 | 1 | 9 |
| MF | GO:000369 | single-stranded DNA binding | 16/790 | 101/16652 | 2.07E-05 | 0.0042313 | 2.3735221 | ENSMUSG0 | Smug1/Cdc | 16 | 4 | 12 |
| MF | GO:000554 | phospholipid binding | 36/790 | 379/16652 | 6.05E-05 | 0.0099114 | 2.0038652 | ENSMUSG0 | Syt5/Rundc | 36 | 21 | 15 |
| MF | GO:001664 | oxidoreductase activity, acting on the CH-NH group of donors | 7/790 | 25/16652 | 0.0001195 | 0.0163079 | 1.7876017 | ENSMUSG0 | Aldh1l2/Mt | 7 | 3 | 4 |
| BP | GO:004202 | protein refolding | 8/800 | 23/16895 | 6.34E-06 | 0.0185548 | 1.7315428 | ENSMUSG0 | Hsp90aa1/H | 8 | 0 | 8 |
| BP | GO:001490 | smooth muscle cell migration | 15/800 | 85/16895 | 9.59E-06 | 0.0185548 | 1.7315428 | ENSMUSG0 | Itga2/Myc/I | 15 | 14 | 1 |
| BP | GO:001481 | muscle cell migration | 16/800 | 97/16895 | 1.20E-05 | 0.0185548 | 1.7315428 | ENSMUSG0 | Itga2/Myc/I | 16 | 15 | 1 |
| MF | GO:003107 | heat shock protein binding | 17/790 | 134/16652 | 0.0002032 | 0.023769 | 1.6239889 | ENSMUSG0 | Ppid/Hsph1 | 17 | 2 | 15 |
| MF | GO:003021 | semaphorin receptor binding | 6/790 | 20/16652 | 0.0002445 | 0.0250345 | 1.6014615 | ENSMUSG0 | Sema3d/Se | 6 | 6 | 0 |
| MF | GO:000809 | DNA-dependent ATPase activity | 12/790 | 79/16652 | 0.0003279 | 0.029835 | 1.5252736 | ENSMUSG0 | Chtf18/Ercc | 12 | 1 | 11 |
| BP | GO:004665 | tetrahydrofolate metabolic process | 7/800 | 21/16895 | 3.37E-05 | 0.0317668 | 1.4980268 | ENSMUSG0 | Aldh1l2/Mt | 7 | 3 | 4 |
| BP | GO:001491 | positive regulation of smooth muscle cell migration | 11/800 | 54/16895 | 3.70E-05 | 0.0317668 | 1.4980268 | ENSMUSG0 | Itga2/Myc/I | 11 | 10 | 1 |
| BP | GO:000072 | telomere maintenance | 17/800 | 120/16895 | 4.96E-05 | 0.0317668 | 1.4980268 | ENSMUSG0 | Hsp90aa1/N | 17 | 3 | 14 |
| BP | GO:005108 | chaperone cofactor-dependent protein refolding | 8/800 | 30/16895 | 5.64E-05 | 0.0317668 | 1.4980268 | ENSMUSG0 | Hsph1/Hspa | 8 | 0 | 8 |
| BP | GO:000626 | DNA replication | 27/800 | 250/16895 | 5.73E-05 | 0.0317668 | 1.4980268 | ENSMUSG0 | Chtf18/Cdc | 27 | 7 | 20 |
| BP | GO:003220 | telomere organization | 17/800 | 122/16895 | 6.14E-05 | 0.0317668 | 1.4980268 | ENSMUSG0 | Hsp90aa1/N | 17 | 3 | 14 |
| BP | GO:001491 | regulation of smooth muscle cell migration | 13/800 | 78/16895 | 6.94E-05 | 0.0323008 | 1.4907865 | ENSMUSG0 | Itga2/Myc/I | 13 | 12 | 1 |
| MF | GO:000400 | ATP-dependent DNA helicase activity | 7/790 | 31/16652 | 0.0005097 | 0.0417444 | 1.3794022 | ENSMUSG0 | Recql4/Xrcc | 7 | 1 | 6 |
| BP | GO:000072 | double-strand break repair via homologous recombination | 15/800 | 104/16895 | 0.0001104 | 0.0427995 | 1.3685609 | ENSMUSG0 | Tex15/Cdc4 | 15 | 4 | 11 |
| BP | GO:000072 | recombinational repair | 15/800 | 104/16895 | 0.0001104 | 0.0427995 | 1.3685609 | ENSMUSG0 | Tex15/Cdc4 | 15 | 4 | 11 |
| CC | GO:000565 | replication fork | 12/808 | 69/16943 | 9.16E-05 | 0.0483525 | 1.3155814 | ENSMUSG0 | Cdc45/Mcm | 12 | 1 | 11 |
