# Supplementary material for: Magnesium Supplementation Modifies Arthritis Synovial and Splenic Transcriptomic Signatures Including Ferroptosis and Cell Senescence Biological Pathways
Source: Nutrients. 2024 Dec 9;16(23):4247. doi: 10.3390/nu16234247 (PMC11644223; doi:10.3390/nu16234247)
Supplement: Supplementary file 1 [file nutrients-16-04247-s001.zip › Supplementary Table S3. DEG in synovial tissues from Mg2800 versus Mg500 diet mice..pdf]

Table S3. DEG in the synovial tissues of mice on the Mg2800 versus Mg500 diets*.

gene_name	SY_Mg2800	SY_Mg500	log2FoldChange (Mg2800 vs Mg 50	pvalue	padj	-log10(pvalue)	gene_name	gene_description
**Increased expression in Mg2800 versus Mg500**								
Actc1	4978.757337	232.5261704	4.42	0.000937258	0.9999452	3.03	Actc1	actin, alpha, cardiac muscle 1 [Source:MGI Symbol;Acc:MGI:87905]
Snora34	25.67950701	1.338897103	4.08	0.000491894	0.9999452	3.31	Snora34	small nucleolar RNA, H/ACA box 34 [Source:MGI Symbol;Acc:MGI:3819502]
Gm37876	8.284698479	0.802796733	3.32	0.032813185	0.9999452	1.48	Gm37876	predicted gene, 37876 [Source:MGI Symbol;Acc:MGI:5611104]
Gm5605	22.91203593	2.369980214	3.10	0.000589543	0.9999452	3.23	Gm5605	predicted gene 5605 [Source:MGI Symbol;Acc:MGI:3647902]
4930555A03Ri	34.98836722	3.918194609	3.05	0.003478431	0.9999452	2.46	4930555A03Ril	RIKEN cDNA 4930555A03 gene [Source:MGI Symbol;Acc:MGI:1922587]
Gm5780	13.62143414	1.423579489	3.03	0.042955314	0.9999452	1.37	Gm5780	predicted gene 5780 [Source:MGI Symbol;Acc:MGI:3645288]
Gm16016	20.94322602	2.440155971	2.96	0.01572136	0.9999452	1.80	Gm16016	predicted gene 16016 [Source:MGI Symbol;Acc:MGI:3802068]
Slc9a4	10.90757843	1.431125527	2.90	0.012003684	0.9999452	1.92	Slc9a4	solute carrier family 9 (sodium/hydrogen exchanger), member 4 [Source:MGI Symbol;Acc:MGI:105074]
Gm26616	26.68390056	3.700133558	2.84	0.023101532	0.9999452	1.64	Gm26616	predicted gene, 26616 [Source:MGI Symbol;Acc:MGI:5477110]
BC030343	30.87730668	4.528539333	2.72	0.032652895	0.9999452	1.49	BC030343	cDNA sequence BC030343 [Source:MGI Symbol;Acc:MGI:2679267]
Gm20597	38.33547527	5.62897067	2.71	0.016236922	0.9999452	1.79	Gm20597	predicted gene, 20597 [Source:MGI Symbol;Acc:MGI:5295703]
Scel	9.494130202	2.01585908	2.48	0.043896253	0.9999452	1.36	Scel	sciellin [Source:MGI Symbol;Acc:MGI:1891228]
Gm10184	24.94948943	4.366120763	2.36	0.007175588	0.9999452	2.14	Gm10184	predicted pseudogene 10184 [Source:MGI Symbol;Acc:MGI:3704480]
Gm42585	24.04117246	4.227857763	2.35	0.03493462	0.9999452	1.46	Gm42585	predicted gene 42585 [Source:MGI Symbol;Acc:MGI:5662722]
Trpv3	12.64403738	2.766580397	2.33	0.034905727	0.9999452	1.46	Trpv3	transient receptor potential cation channel, subfamily V, member 3 [Source:MGI Symbol;Acc:MGI:2181407]
Tpd52-ps	5.829365988	1.290270774	2.33	0.030812027	0.9999452	1.51	Tpd52-ps	tumor protein D52, pseudogene [Source:MGI Symbol;Acc:MGI:107737]
Gm18949	9.943714541	2.020947859	2.30	0.039269941	0.9999452	1.41	Gm18949	predicted gene, 18949 [Source:MGI Symbol;Acc:MGI:5011134]
Nr4a3	437.5761901	93.09957138	2.23	0.024930973	0.9999452	1.60	Nr4a3	nuclear receptor subfamily 4, group A, member 3 [Source:MGI Symbol;Acc:MGI:1352457]
2310043M15R	25.72924504	5.751898364	2.22	0.009131572	0.9999452	2.04	2310043M15Ri	RIKEN cDNA 2310043M15 gene [Source:MGI Symbol;Acc:MGI:1919180]
Gm19710	31.8921623	6.96852462	2.21	0.031851678	0.9999452	1.50	Gm19710	predicted gene, 19710 [Source:MGI Symbol;Acc:MGI:5011895]
Cspg5	12.50493183	2.751920161	2.19	0.030149555	0.9999452	1.52	Cspg5	chondroitin sulfate proteoglycan 5 [Source:MGI Symbol;Acc:MGI:1352747]
Lekr1	47.67934335	10.71041026	2.14	0.002913519	0.9999452	2.54	Lekr1	leucine, glutamate and lysine rich 1 [Source:MGI Symbol;Acc:MGI:3645902]
Gm26719	32.79493458	7.302010607	2.11	0.017718082	0.9999452	1.75	Gm26719	predicted gene, 26719 [Source:MGI Symbol;Acc:MGI:5477213]
Tgm4	44.84687999	10.37144997	2.09	0.018395356	0.9999452	1.74	Tgm4	transglutaminase 4 (prostate) [Source:MGI Symbol;Acc:MGI:3027002]
Gm21887	102.3368158	24.13449109	2.08	0.030004294	0.9999452	1.52	Gm21887	predicted gene, 21887 [Source:MGI Symbol;Acc:MGI:5434051]
Gm15751	76.17322561	17.55980165	2.08	0.008960425	0.9999452	2.05	Gm15751	predicted gene 15751 [Source:MGI Symbol;Acc:MGI:3783193]
Gm10044	25.35825649	5.689955912	2.07	0.047778352	0.9999452	1.32	Gm10044	predicted gene 10044 [Source:MGI Symbol;Acc:MGI:3641749]
Gm16894	22.46475983	5.036536064	2.04	0.036158273	0.9999452	1.44	Gm16894	predicted gene, 16894 [Source:MGI Symbol;Acc:MGI:4439818]
Gm973	34.59800522	8.421943915	2.02	0.045284292	0.9999452	1.34	Gm973	predicted gene 973 [Source:MGI Symbol;Acc:MGI:2685819]
C7	518.7923239	128.8276672	2.01	0.031043246	0.9999452	1.51	C7	complement component 7 [Source:MGI Symbol;Acc:MGI:88235]
Gnrh1	57.52593229	14.08804356	2.00	0.000455323	0.9999452	3.34	Gnrh1	gonadotropin releasing hormone 1 [Source:MGI Symbol;Acc:MGI:95789]
1810010D01Ri	21.23906023	5.238699012	1.95	0.046533376	0.9999452	1.33	1810010D01Ril	RIKEN cDNA 1810010D01 gene [Source:MGI Symbol;Acc:MGI:1916283]
Gm29787	13.94845821	3.567848603	1.91	0.047242933	0.9999452	1.33	Gm29787	predicted gene, 29787 [Source:MGI Symbol;Acc:MGI:5588946]
Gm5555	46.49317966	12.51400407	1.85	0.004782951	0.9999452	2.32	Gm5555	predicted pseudogene 5555 [Source:MGI Symbol;Acc:MGI:3648970]
Gm47583	17.14464278	4.828936376	1.83	0.040109919	0.9999452	1.40	Gm47583	predicted gene, 47583 [Source:MGI Symbol;Acc:MGI:6096621]
Gm17907	49.05558528	13.72800409	1.78	0.0474786	0.9999452	1.32	Gm17907	predicted gene, 17907 [Source:MGI Symbol;Acc:MGI:5010092]
Gm42732	75.92858156	22.01951558	1.76	0.03392944	0.9999452	1.47	Gm42732	predicted gene 42732 [Source:MGI Symbol;Acc:MGI:5662869]
Clvs1	19.57046356	5.792465286	1.67	0.049126289	0.9999452	1.31	Clvs1	clavesin 1 [Source:MGI Symbol;Acc:MGI:1921688]
B130024G19Ri	66.56820046	21.0346271	1.65	0.02477194	0.9999452	1.61	B130024G19Ri	RIKEN cDNA B130024G19 gene [Source:MGI Symbol;Acc:MGI:3604353]
Gm5763	26.84371425	8.22122468	1.62	0.0246532	0.9999452	1.61	Gm5763	predicted pseudogene 5763 [Source:MGI Symbol;Acc:MGI:3647841]
Gm17690	59.98995307	20.54751161	1.53	0.031436221	0.9999452	1.50	Gm17690	predicted gene, 17690 [Source:MGI Symbol;Acc:MGI:4937324]
Gm17275	152.3569042	56.35073224	1.43	0.017368019	0.9999452	1.76	Gm17275	predicted gene, 17275 [Source:MGI Symbol;Acc:MGI:4936909]
Fam161b	37.56308631	14.58269395	1.31	0.00606532	0.9999452	2.22	Fam161b	family with sequence similarity 161, member B [Source:MGI Symbol;Acc:MGI:2443027]
Gm8399	48.88504075	20.04278499	1.25	0.042652578	0.9999452	1.37	Gm8399	predicted gene 8399 [Source:MGI Symbol;Acc:MGI:3647971]
Cacna1h	176.7768649	74.47249456	1.21	0.016276441	0.9999452	1.79	Cacna1h	calcium channel, voltage-dependent, T type, alpha 1H subunit [Source:MGI Symbol;Acc:MGI:1928842]
Nr4a1	2302.908979	1022.486277	1.17	0.028298173	0.9999452	1.55	Nr4a1	nuclear receptor subfamily 4, group A, member 1 [Source:MGI Symbol;Acc:MGI:1352454]
D230017M19R	41.17705768	17.14033642	1.16	0.039768502	0.9999452	1.40	D230017M19R	RIKEN cDNA D230017M19 gene [Source:MGI Symbol;Acc:MGI:2445071]
Gm12404	52.51473522	22.215791	1.15	0.029888614	0.9999452	1.52	Gm12404	predicted gene 12404 [Source:MGI Symbol;Acc:MGI:3649601]
Sik1	1218.81674	572.5745014	1.09	0.029390721	0.9999452	1.53	Sik1	salt inducible kinase 1 [Source:MGI Symbol;Acc:MGI:104754]
Per1	1437.216238	677.3885682	1.09	0.0130787	0.9999452	1.88	Per1	period circadian clock 1 [Source:MGI Symbol;Acc:MGI:1098283]
4732440D04Ri	71.80275245	34.35353511	1.08	0.003243548	0.9999452	2.49	4732440D04Ril	RIKEN cDNA 4732440D04 gene [Source:MGI Symbol;Acc:MGI:3604103]
AC126457.1	63.54975397	28.36431434	1.07	0.027212804	0.9999452	1.57	AC126457.1	NSE3 homolog, SMC5-SMC6 complex component (Nsmce3) pseudogene
Slc52a3	104.7406628	49.50308152	1.07	0.015250653	0.9999452	1.82	Slc52a3	solute carrier protein family 52, member 3 [Source:MGI Symbol;Acc:MGI:1916948]
Dtwd1	96.4602302	45.32398449	1.06	0.030621614	0.9999452	1.51	Dtwd1	DTW domain containing 1 [Source:MGI Symbol;Acc:MGI:1916435]
Ppp1r26	50.69374132	23.70370937	1.05	0.048441716	0.9999452	1.31	Ppp1r26	protein phosphatase 1, regulatory subunit 26 [Source:MGI Symbol;Acc:MGI:2685193]
Meg3	1401.210509	679.7411092	1.04	0.02647853	0.9999452	1.58	Meg3	maternally expressed 3 [Source:MGI Symbol;Acc:MGI:1202886]
Cbarp	132.1506271	65.26075202	1.02	0.037536289	0.9999452	1.43	Cbarp	calcium channel, voltage-dependent, beta subunit associated regulatory protein [Source:MGI Symbol;Acc:MGI:1354170]
Gm12940	438.6617761	218.1299552	1.01	0.019530242	0.9999452	1.71	Gm12940	predicted gene 12940 [Source:MGI Symbol;Acc:MGI:3702626]

Hoxb9	31.44473798	16.50045785	1.00	0.038007405	0.9999452	1.42	Hoxb9	homeobox B9 [Source:MGI Symbol;Acc:MGI:96190]
Gm10125	67.371545	32.2376464	0.99	0.033615644	0.9999452	1.47	Gm10125	predicted gene 10125 [Source:MGI Symbol;Acc:MGI:3642044]
Otud1	359.7630105	181.6511898	0.98	0.040225127	0.9999452	1.40	Otud1	OTU domain containing 1 [Source:MGI Symbol;Acc:MGI:1918448]
Gm47071	89.57234399	45.27495953	0.95	0.032894056	0.9999452	1.48	Gm47071	predicted gene, 47071 [Source:MGI Symbol;Acc:MGI:6095789]
Pde4b	1104.505691	606.8494998	0.86	0.044645707	0.9999452	1.35	Pde4b	phosphodiesterase 4B, cAMP specific [Source:MGI Symbol;Acc:MGI:99557]
Rem1	156.6372195	87.61356799	0.80	0.033664169	0.9999452	1.47	Rem1	rad and gem related GTP binding protein 1 [Source:MGI Symbol;Acc:MGI:1097696]
Ccdc141	80.02736101	46.77060338	0.80	0.031691678	0.9999452	1.50	Ccdc141	coiled-coil domain containing 141 [Source:MGI Symbol;Acc:MGI:1919735]
Vsig10l	132.2217111	76.32149237	0.78	0.019817338	0.9999452	1.70	Vsig10l	V-set and immunoglobulin domain containing 10 like [Source:MGI Symbol;Acc:MGI:1922940]
Clk1	2125.527627	1259.358802	0.76	0.027116598	0.9999452	1.57	Clk1	CDC-like kinase 1 [Source:MGI Symbol;Acc:MGI:107403]
Nxpe4	361.3903483	214.8611165	0.73	0.027207801	0.9999452	1.57	Nxpe4	neurexophilin and PC-esterase domain family, member 4 [Source:MGI Symbol;Acc:MGI:1924792]
Pnn	1147.092351	752.0950935	0.61	0.029422103	0.9999452	1.53	Pnn	pinin [Source:MGI Symbol;Acc:MGI:1100514]
Ambra1	823.8615045	571.5736509	0.52	0.033097	0.9999452	1.48	Ambra1	autophagy/beclin 1 regulator 1 [Source:MGI Symbol;Acc:MGI:2443564]
Angel2	937.7926291	730.2155062	0.36	0.027794263	0.9999452	1.56	Angel2	angel homolog 2 [Source:MGI Symbol;Acc:MGI:1196310]
Cdc42ep1	558.5019605	899.5604739	-0.69	0.044930091	0.9999452	1.35	Cdc42ep1	CDC42 effector protein (Rho GTPase binding) 1 [Source:MGI Symbol;Acc:MGI:1929763]
**Decreased expression in Mg2800 versus Mg500**								
Krt79	6.075228	281.1829497	-5.46	7.02E-06	0.1932448	5.15	Krt79	keratin 79 [Source:MGI Symbol;Acc:MGI:2385030]
Hist1h3i	0.555260765	19.07649027	-4.75	0.012775152	0.9999452	1.89	Hist1h3i	histone cluster 1, H3i [Source:MGI Symbol;Acc:MGI:2448350]
Hist1h4c	1.004690888	20.09969293	-4.08	0.011355946	0.9999452	1.94	Hist1h4c	histone cluster 1, H4c [Source:MGI Symbol;Acc:MGI:2448421]
Snord104	6.801323759	114.78407	-4.04	0.012126773	0.9999452	1.92	Snord104	small nucleolar RNA, C/D box 104 [Source:MGI Symbol;Acc:MGI:3819515]
AI463229	1.412117027	24.81361362	-3.95	0.003954366	0.9999452	2.40	AI463229	expressed sequence AI463229 [Source:MGI Symbol;Acc:MGI:2145255]
Hist1h1b	2.71595678	42.77942697	-3.88	0.005273484	0.9999452	2.28	Hist1h1b	histone cluster 1, H1b [Source:MGI Symbol;Acc:MGI:1861461]
Igha	80.14947573	1157.434311	-3.85	0.012321073	0.9999452	1.91	Igha	immunoglobulin heavy constant alpha [Source:MGI Symbol;Acc:MGI:96444]
Gm9385	12.20718986	171.0365875	-3.78	0.000236804	0.9999452	3.63	Gm9385	predicted pseudogene 9385 [Source:MGI Symbol;Acc:MGI:3646182]
Rps12-ps4	4.682682442	54.53071044	-3.72	0.002817628	0.9999452	2.55	Rps12-ps4	ribosomal protein S12, pseudogene 4 [Source:MGI Symbol;Acc:MGI:3780139]
Gm38299	0.97785494	15.16705188	-3.70	0.007756923	0.9999452	2.11	Gm38299	predicted gene, 38299 [Source:MGI Symbol;Acc:MGI:5611527]
Gcat	14.91132808	168.6346152	-3.63	0.00010264	0.7062425	3.99	Gcat	glycine C-acetyltransferase (2-amino-3-ketobutyrate-coenzyme A ligase) [Source:MGI Symbol;Acc:MGI:1349389]
2610016A17Ri	3.920293775	50.20178051	-3.59	0.005418625	0.9999452	2.27	2610016A17Ril	RIKEN cDNA 2610016A17 gene [Source:MGI Symbol;Acc:MGI:1919653]
Usp50	5.123923582	57.770729	-3.51	0.000223635	0.9999452	3.65	Usp50	ubiquitin specific peptidase 50 [Source:MGI Symbol;Acc:MGI:1922333]
Camp	41.35117516	448.1704091	-3.43	0.014569083	0.9999452	1.84	Camp	cathelicidin antimicrobial peptide [Source:MGI Symbol;Acc:MGI:108443]
Gm16418	19.03098121	195.0260136	-3.42	0.001176458	0.9999452	2.93	Gm16418	predicted pseudogene 16418 [Source:MGI Symbol;Acc:MGI:3644548]
Elane	18.38158634	199.5398344	-3.42	0.015347459	0.9999452	1.81	Elane	elastase, neutrophil expressed [Source:MGI Symbol;Acc:MGI:2679229]
Mpo	31.65817864	331.0693139	-3.37	0.015892341	0.9999452	1.80	Mpo	myeloperoxidase [Source:MGI Symbol;Acc:MGI:97137]
Prg2	7.588300087	79.17920459	-3.32	0.004544695	0.9999452	2.34	Prg2	proteoglycan 2, bone marrow [Source:MGI Symbol;Acc:MGI:103294]
Fgf21	1.043103125	11.79108633	-3.25	0.047531952	0.9999452	1.32	Fgf21	fibroblast growth factor 21 [Source:MGI Symbol;Acc:MGI:1861377]
Gm5561	1.622264672	9.425572283	-3.23	0.03248609	0.9999452	1.49	Gm5561	predicted gene 5561 [Source:MGI Symbol;Acc:MGI:3804971]
Gm47644	2.803136431	28.87405457	-3.22	0.010635257	0.9999452	1.97	Gm47644	predicted gene, 47644 [Source:MGI Symbol;Acc:MGI:6096722]
Gm37108	0.666866034	8.019738136	-3.22	0.010165423	0.9999452	1.99	Gm37108	predicted gene, 37108 [Source:MGI Symbol;Acc:MGI:5610336]
Bglap	96.79793031	875.2675411	-3.17	0.014966015	0.9999452	1.82	Bglap	bone gamma carboxyglutamate protein [Source:MGI Symbol;Acc:MGI:88156]
Mettl5os	2.382290334	23.0230997	-3.16	0.047708168	0.9999452	1.32	Mettl5os	methyltransferase like 5, opposite strand [Source:MGI Symbol;Acc:MGI:1922537]
Gm42876	4.074257753	37.56925	-3.13	0.018906797	0.9999452	1.72	Gm42876	predicted gene 42876 [Source:MGI Symbol;Acc:MGI:5663013]
Sp7	45.92966071	398.9023754	-3.11	0.032998093	0.9999452	1.48	Sp7	Sp7 transcription factor 7 [Source:MGI Symbol;Acc:MGI:2153568]
Slurp1	4.343347676	39.01472061	-3.10	0.0196765	0.9999452	1.71	Slurp1	secreted Ly6/Plaur domain containing 1 [Source:MGI Symbol;Acc:MGI:1930923]
Irgc1	5.545026216	48.7706923	-3.09	0.047055959	0.9999452	1.33	Irgc1	immunity-related GTPase family, cinema 1 [Source:MGI Symbol;Acc:MGI:2685948]
Phyhip	5.988055987	53.00880895	-3.07	0.005559659	0.9999452	2.25	Phyhip	phytanoyl-CoA hydroxylase interacting protein [Source:MGI Symbol;Acc:MGI:1860417]
Calml3	4.787917733	42.05324393	-3.07	0.042599517	0.9999452	1.37	Calml3	calmodulin-like 3 [Source:MGI Symbol;Acc:MGI:1917655]
Smim27	9.729198966	84.67117058	-3.07	0.008152685	0.9999452	2.09	Smim27	small integral membrane protein 27 [Source:MGI Symbol;Acc:MGI:1913684]
Gm14794	1.928163982	17.85976018	-3.06	0.026069485	0.9999452	1.58	Gm14794	predicted gene 14794 [Source:MGI Symbol;Acc:MGI:3645637]
Gm11942	9.765967985	81.39082662	-3.04	0.001948092	0.9999452	2.71	Gm11942	predicted gene 11942 [Source:MGI Symbol;Acc:MGI:3650608]
Sncg	333.385661	2726.184071	-3.04	0.001311429	0.9999452	2.88	Sncg	synuclein, gamma [Source:MGI Symbol;Acc:MGI:1298397]
Gm10029	1.482160373	13.66713106	-3.01	0.048500653	0.9999452	1.31	Gm10029	predicted gene 10029 [Source:MGI Symbol;Acc:MGI:3641706]
2310034G01Ri	2.029298396	17.80844258	-3.00	0.010092834	0.9999452	2.00	2310034G01Ril	RIKEN cDNA 2310034G01 gene [Source:MGI Symbol;Acc:MGI:1922829]
D030025P21Ri	2.781427215	23.25654385	-2.96	0.032969196	0.9999452	1.48	D030025P21Ril	RIKEN cDNA D030025P21 gene [Source:MGI Symbol;Acc:MGI:3698049]
Ltc4s	52.10911185	401.5706343	-2.94	0.000778935	0.9999452	3.11	Ltc4s	leukotriene C4 synthase [Source:MGI Symbol;Acc:MGI:107498]
Rpl41	2966.527882	22748.19483	-2.94	0.00104706	0.9999452	2.98	Rpl41	ribosomal protein L41 [Source:MGI Symbol;Acc:MGI:1915195]
Gm10240	2.500003077	15.56249978	-2.93	0.010394251	0.9999452	1.98	Gm10240	predicted gene 10240 [Source:MGI Symbol;Acc:MGI:3704454]
A330069E16Ri	2.35478751	19.66481777	-2.92	0.031214002	0.9999452	1.51	A330069E16Ril	RIKEN cDNA A330069E16 gene [Source:MGI Symbol;Acc:MGI:3583899]
Ffar2	117.6481261	882.2130117	-2.91	0.001614044	0.9999452	2.79	Ffar2	free fatty acid receptor 2 [Source:MGI Symbol;Acc:MGI:2441731]
Pet100	57.40651984	413.4993078	-2.88	0.009586031	0.9999452	2.02	Pet100	PET100 homolog [Source:MGI Symbol;Acc:MGI:3615306]
Fcor	18.76737755	133.0253903	-2.88	0.005965822	0.9999452	2.22	Fcor	Foxo1 corepressor [Source:MGI Symbol;Acc:MGI:1915484]
Gm14586	16.86697452	120.2181477	-2.84	0.001351586	0.9999452	2.87	Gm14586	predicted gene 14586 [Source:MGI Symbol;Acc:MGI:3705507]
Slc6a13	22.26201952	162.2092106	-2.82	0.004137281	0.9999452	2.38	Slc6a13	solute carrier family 6 (neurotransmitter transporter, GABA), member 13 [Source:MGI Symbol;Acc:MGI:95629]

Mt3	11.64615447	85.46650963	-2.82	0.004154051	0.9999452	2.38	Mt3	metallothionein 3 [Source:MGI Symbol;Acc:MGI:97173]
Gm10277	3.193691713	24.76111914	-2.81	0.001842825	0.9999452	2.73	Gm10277	predicted gene 10277 [Source:MGI Symbol;Acc:MGI:3641921]
Slc39a5	6.94326023	50.65432219	-2.80	0.01237201	0.9999452	1.91	Slc39a5	solute carrier family 39 (metal ion transporter), member 5 [Source:MGI Symbol;Acc:MGI:1919336]
Gm37716	1.392846857	11.18571354	-2.80	0.038469456	0.9999452	1.41	Gm37716	predicted gene, 37716 [Source:MGI Symbol;Acc:MGI:5610944]
Gm12411	30.86932342	201.4107962	-2.76	0.001893905	0.9999452	2.72	Gm12411	predicted gene 12411 [Source:MGI Symbol;Acc:MGI:3649918]
Gabra2	3.301673719	23.17981666	-2.71	0.042204192	0.9999452	1.37	Gabra2	gamma-aminobutyric acid (GABA) A receptor, subunit alpha 2 [Source:MGI Symbol;Acc:MGI:95614]
Apoc1	87.05018062	559.8913347	-2.69	0.007519155	0.9999452	2.12	Apoc1	apolipoprotein C-I [Source:MGI Symbol;Acc:MGI:88053]
Dmp1	55.10679993	357.0022776	-2.69	0.043621019	0.9999452	1.36	Dmp1	dentin matrix protein 1 [Source:MGI Symbol;Acc:MGI:94910]
Etfb	202.7207695	1293.796227	-2.69	0.022531097	0.9999452	1.65	Etfb	electron transferring flavoprotein, beta polypeptide [Source:MGI Symbol;Acc:MGI:106098]
Ccl21a	81.96426794	528.871877	-2.68	0.029510819	0.9999452	1.53	Ccl21a	chemokine (C-C motif) ligand 21A (serine) [Source:MGI Symbol;Acc:MGI:1349183]
Trappc6a	96.55991352	624.2348726	-2.68	0.003056794	0.9999452	2.51	Trappc6a	trafficking protein particle complex 6A [Source:MGI Symbol;Acc:MGI:1914341]
Gm13868	6.586877882	44.64055668	-2.68	0.010715559	0.9999452	1.97	Gm13868	predicted gene 13868 [Source:MGI Symbol;Acc:MGI:3651789]
Atp5k	344.2428369	2181.886844	-2.67	0.034541449	0.9999452	1.46	Atp5k	ATP synthase, H+ transporting, mitochondrial F1F0 complex, subunit E [Source:MGI Symbol;Acc:MGI:106636]
Pnp2	7.48191546	48.83024565	-2.65	0.02629694	0.9999452	1.58	Pnp2	purine-nucleoside phosphorylase 2 [Source:MGI Symbol;Acc:MGI:3712328]
0610040B10Ri	4.738490101	32.1686678	-2.65	0.005506526	0.9999452	2.26	0610040B10Ril	RIKEN cDNA 0610040B10 gene [Source:MGI Symbol;Acc:MGI:1914922]
Bglap2	98.76015568	620.8820631	-2.65	0.031675278	0.9999452	1.50	Bglap2	bone gamma-carboxyglutamate protein 2 [Source:MGI Symbol;Acc:MGI:88157]
S1pr5	3.449658918	23.19041823	-2.61	0.010801912	0.9999452	1.97	S1pr5	sphingosine-1-phosphate receptor 5 [Source:MGI Symbol;Acc:MGI:2150641]
Snrpd2	365.9849618	2232.233701	-2.61	0.003391994	0.9999452	2.47	Snrpd2	small nuclear ribonucleoprotein D2 [Source:MGI Symbol;Acc:MGI:98345]
Ptprz1	12.4783471	76.41017596	-2.58	0.045761487	0.9999452	1.34	Ptprz1	protein tyrosine phosphatase, receptor type Z, polypeptide 1 [Source:MGI Symbol;Acc:MGI:97816]
Chrna1os	4.027200594	25.27451365	-2.57	0.033051217	0.9999452	1.48	Chrna1os	cholinergic receptor, nicotinic, alpha polypeptide 1 (muscle), opposite strand [Source:MGI Symbol;Acc:MGI:3651120]
Gm42847	5.469221317	33.93503813	-2.54	0.02663669	0.9999452	1.57	Gm42847	predicted gene 42847 [Source:MGI Symbol;Acc:MGI:5662984]
Fkbp2	255.2750478	1480.696632	-2.54	0.002088073	0.9999452	2.68	Fkbp2	FK506 binding protein 2 [Source:MGI Symbol;Acc:MGI:95542]
Gm10563	6.706556515	40.20596785	-2.51	0.037310415	0.9999452	1.43	Gm10563	predicted gene 10563 [Source:MGI Symbol;Acc:MGI:3642630]
Gm8242	10.02934988	53.02747891	-2.50	0.000768825	0.9999452	3.11	Gm8242	predicted gene 8242 [Source:MGI Symbol;Acc:MGI:3647216]
Gm7331	11.38767851	59.44405321	-2.49	0.004290104	0.9999452	2.37	Gm7331	predicted gene 7331 [Source:MGI Symbol;Acc:MGI:3644702]
Fam84a	5.075082288	30.24352203	-2.48	0.021110528	0.9999452	1.68	Fam84a	family with sequence similarity 84, member A [Source:MGI Symbol;Acc:MGI:2145011]
Syp	16.98879713	96.94134293	-2.47	0.015796357	0.9999452	1.80	Syp	synaptophysin [Source:MGI Symbol;Acc:MGI:98467]
Gm14719	5.953598003	26.78960415	-2.47	0.007746929	0.9999452	2.11	Gm14719	predicted gene 14719 [Source:MGI Symbol;Acc:MGI:3705771]
S100a13	333.2283377	1824.600664	-2.46	0.002901963	0.9999452	2.54	S100a13	S100 calcium binding protein A13 [Source:MGI Symbol;Acc:MGI:109581]
Lipc	9.22858261	52.63826274	-2.44	0.006115019	0.9999452	2.21	Lipc	lipase, hepatic [Source:MGI Symbol;Acc:MGI:96216]
Gm10269	5.82738392	31.26538231	-2.43	0.024410703	0.9999452	1.61	Gm10269	predicted gene 10269 [Source:MGI Symbol;Acc:MGI:3642381]
Rpl36-ps12	22.99269801	117.070626	-2.42	0.009165118	0.9999452	2.04	Rpl36-ps12	ribosomal protein L36, pseudogene 12 [Source:MGI Symbol;Acc:MGI:3782787]
Retn	656.9086468	3517.119501	-2.42	0.02683323	0.9999452	1.57	Retn	resistin [Source:MGI Symbol;Acc:MGI:1888506]
Cd59b	7.290017688	41.53673649	-2.42	0.022862652	0.9999452	1.64	Cd59b	CD59b antigen [Source:MGI Symbol;Acc:MGI:1888996]
Adig	178.264626	938.2275515	-2.40	0.023225374	0.9999452	1.63	Adig	adipogenin [Source:MGI Symbol;Acc:MGI:2675492]
Satb2	58.35769958	309.7004471	-2.40	0.033594072	0.9999452	1.47	Satb2	special AT-rich sequence binding protein 2 [Source:MGI Symbol;Acc:MGI:2679336]
Guca1a	16.48183718	88.99947341	-2.39	0.012563146	0.9999452	1.90	Guca1a	guanylate cyclase activator 1a (retina) [Source:MGI Symbol;Acc:MGI:102770]
Atox1	452.3981209	2335.780349	-2.37	0.004455428	0.9999452	2.35	Atox1	antioxidant 1 copper chaperone [Source:MGI Symbol;Acc:MGI:1333855]
Gm44763	4.185828541	23.34787448	-2.36	0.025411465	0.9999452	1.59	Gm44763	predicted gene 44763 [Source:MGI Symbol;Acc:MGI:5753339]
Gm9844	27.82222627	145.3597014	-2.35	0.012580723	0.9999452	1.90	Gm9844	predicted pseudogene 9844 [Source:MGI Symbol;Acc:MGI:3704288]
Rbp7	22.2375104	115.6745948	-2.35	0.049602293	0.9999452	1.30	Rbp7	retinol binding protein 7, cellular [Source:MGI Symbol;Acc:MGI:1890409]
Fam241b	3.947438574	21.73674081	-2.34	0.026847822	0.9999452	1.57	Fam241b	family with sequence similarity 241, member B [Source:MGI Symbol;Acc:MGI:1917144]
Gm6311	12.90753906	59.48576794	-2.32	0.00923387	0.9999452	2.03	Gm6311	predicted gene 6311 [Source:MGI Symbol;Acc:MGI:3644735]
Gm7206	4.11534604	19.41203405	-2.31	0.025329785	0.9999452	1.60	Gm7206	predicted pseudogene 7206 [Source:MGI Symbol;Acc:MGI:3646634]
Rps27a	1487.937921	7355.995353	-2.31	0.008453989	0.9999452	2.07	Rps27a	ribosomal protein S27A [Source:MGI Symbol;Acc:MGI:1925544]
Ifi27l2a	761.5777041	3755.498685	-2.30	0.013414372	0.9999452	1.87	Ifi27l2a	interferon, alpha-inducible protein 27 like 2A [Source:MGI Symbol;Acc:MGI:1924183]
Gm15952	3.564193858	18.99637162	-2.30	0.040304619	0.9999452	1.39	Gm15952	predicted gene 15952 [Source:MGI Symbol;Acc:MGI:3802149]
Gm14207	19.12955297	96.15026628	-2.29	0.021607186	0.9999452	1.67	Gm14207	predicted gene 14207 [Source:MGI Symbol;Acc:MGI:3649550]
Acoxl	4.748643455	24.81494033	-2.29	0.034803579	0.9999452	1.46	Acoxl	acyl-Coenzyme A oxidase-like [Source:MGI Symbol;Acc:MGI:1921371]
Stmn3	2.53932908	13.60426561	-2.26	0.030553837	0.9999452	1.51	Stmn3	stathmin-like 3 [Source:MGI Symbol;Acc:MGI:1277137]
Gm11730	2.344623066	12.17140539	-2.23	0.043059838	0.9999452	1.37	Gm11730	predicted gene 11730 [Source:MGI Symbol;Acc:MGI:3650941]
Romo1	261.8272989	1211.958446	-2.22	0.019375763	0.9999452	1.71	Romo1	reactive oxygen species modulator 1 [Source:MGI Symbol;Acc:MGI:1914317]
Gchfr	5.473905421	26.71861188	-2.21	0.046778853	0.9999452	1.33	Gchfr	GTP cyclohydrolase I feedback regulator [Source:MGI Symbol;Acc:MGI:2443977]
Tomm7	185.4731838	842.383838	-2.19	0.019822285	0.9999452	1.70	Tomm7	translocase of outer mitochondrial membrane 7 [Source:MGI Symbol;Acc:MGI:1913419]
Vkorc1	41.03160671	190.047653	-2.19	0.018615702	0.9999452	1.73	Vkorc1	vitamin K epoxide reductase complex, subunit 1 [Source:MGI Symbol;Acc:MGI:106442]
Rplp1	9044.528471	41256.64179	-2.19	0.014051739	0.9999452	1.85	Rplp1	ribosomal protein, large, P1 [Source:MGI Symbol;Acc:MGI:1927099]
Sptbn4	3.860221338	19.14935892	-2.19	0.019214719	0.9999452	1.72	Sptbn4	spectrin beta, non-erythrocytic 4 [Source:MGI Symbol;Acc:MGI:1890574]
Gm4705	3.814050472	16.27287642	-2.18	0.049653825	0.9999452	1.30	Gm4705	predicted gene 4705 [Source:MGI Symbol;Acc:MGI:3782885]
Snrpe	114.4153608	509.4413784	-2.15	0.005320821	0.9999452	2.27	Snrpe	small nuclear ribonucleoprotein E [Source:MGI Symbol;Acc:MGI:98346]
Gm11423	10.824289	50.8968337	-2.15	0.00933171	0.9999452	2.03	Gm11423	predicted gene 11423 [Source:MGI Symbol;Acc:MGI:3651335]
Rpl30-ps1	16.47390992	70.0317871	-2.12	0.01563451	0.9999452	1.81	Rpl30-ps1	ribosomal protein L30, pseudogene 1 [Source:MGI Symbol;Acc:MGI:1321399]

Trf	203.5457334	883.9564936	-2.12	0.010943939	0.9999452	1.96	Trf	transferrin [Source:MGI Symbol;Acc:MGI:98821]
Pmvk	231.924187	1001.662232	-2.12	0.019694766	0.9999452	1.71	Pmvk	phosphomevalonate kinase [Source:MGI Symbol;Acc:MGI:1915853]
Dbi	1501.440115	6496.492335	-2.11	0.019114661	0.9999452	1.72	Dbi	diazepam binding inhibitor [Source:MGI Symbol;Acc:MGI:94865]
Clec4b1	5.027380788	23.28807348	-2.11	0.016265871	0.9999452	1.79	Clec4b1	C-type lectin domain family 4, member b1 [Source:MGI Symbol;Acc:MGI:1917060]
Rpl28-ps1	140.6335865	603.6138738	-2.11	0.00648068	0.9999452	2.19	Rpl28-ps1	ribosomal protein L28, pseudogene 1 [Source:MGI Symbol;Acc:MGI:3705349]
Hddc2	129.7545609	551.1078693	-2.10	0.005698272	0.9999452	2.24	Hddc2	HD domain containing 2 [Source:MGI Symbol;Acc:MGI:1916942]
1700037C18Ri	24.08996367	105.3919971	-2.10	0.039627832	0.9999452	1.40	1700037C18Ril	RIKEN cDNA 1700037C18 gene [Source:MGI Symbol;Acc:MGI:1920511]
H2afj	423.1495168	1784.549278	-2.08	0.011300876	0.9999452	1.95	H2afj	H2A histone family, member J [Source:MGI Symbol;Acc:MGI:3606192]
Lsm7	122.4630724	506.5792426	-2.06	0.035897472	0.9999452	1.44	Lsm7	LSM7 homolog, U6 small nuclear RNA and mRNA degradation associated [Source:MGI Symbol;Acc:MGI:1913344]
Ndufa1	216.5036126	896.6420775	-2.06	0.025114582	0.9999452	1.60	Ndufa1	NADH:ubiquinone oxidoreductase subunit A1 [Source:MGI Symbol;Acc:MGI:1929511]
Ifitm6	33.44581824	143.2975209	-2.06	0.00510733	0.9999452	2.29	Ifitm6	interferon induced transmembrane protein 6 [Source:MGI Symbol;Acc:MGI:2686976]
Gm26917	6455.788059	26875.78752	-2.06	0.014974097	0.9999452	1.82	Gm26917	predicted gene, 26917 [Source:MGI Symbol;Acc:MGI:5504032]
AC121151.1	33.02654447	133.9483656	-2.04	0.022619043	0.9999452	1.65	AC121151.1	ribosomal protein L7A (Rpl7a) pseudogene
Smim4	68.42474553	283.7423359	-2.04	0.013818832	0.9999452	1.86	Smim4	small integral membrane protein 4 [Source:MGI Symbol;Acc:MGI:1913737]
Rpl17-ps9	4.551452633	20.74770756	-2.03	0.009976709	0.9999452	2.00	Rpl17-ps9	ribosomal protein L17, pseudogene 9 [Source:MGI Symbol;Acc:MGI:3641818]
Snhg8	96.8382812	389.9597253	-2.02	0.015106594	0.9999452	1.82	Snhg8	small nucleolar RNA host gene 8 [Source:MGI Symbol;Acc:MGI:1917145]
Saysd1	73.2824975	300.0621638	-2.02	0.011459358	0.9999452	1.94	Saysd1	SAYSVFN motif domain containing 1 [Source:MGI Symbol;Acc:MGI:1914759]
Pth1r	557.101456	2237.219179	-2.01	0.000353373	0.9999452	3.45	Pth1r	parathyroid hormone 1 receptor [Source:MGI Symbol;Acc:MGI:97801]
Aldh3a1	33.26191345	135.6738434	-2.00	0.03098121	0.9999452	1.51	Aldh3a1	aldehyde dehydrogenase family 3, subfamily A1 [Source:MGI Symbol;Acc:MGI:1353451]
Gm10177	57.57230088	231.9636994	-2.00	0.006811035	0.9999452	2.17	Gm10177	predicted gene 10177 [Source:MGI Symbol;Acc:MGI:3704330]
Vamp8	500.8437494	1980.163765	-1.98	0.007441954	0.9999452	2.13	Vamp8	vesicle-associated membrane protein 8 [Source:MGI Symbol;Acc:MGI:1336882]
Gm10461	7.414135817	32.61682483	-1.98	0.000857758	0.9999452	3.07	Gm10461	predicted gene 10461 [Source:MGI Symbol;Acc:MGI:3642164]
Naa38	95.85889194	373.2785498	-1.97	0.005505797	0.9999452	2.26	Naa38	N(alpha)-acetyltransferase 38, NatC auxiliary subunit [Source:MGI Symbol;Acc:MGI:1925554]
Atad3aos	5.281701237	22.18807697	-1.97	0.049326736	0.9999452	1.31	Atad3aos	ATPase family, AAA domain containing 3A, opposite strand [Source:MGI Symbol;Acc:MGI:1917698]
Gm9320	7.22762574	30.16769432	-1.96	0.023637468	0.9999452	1.63	Gm9320	predicted gene 9320 [Source:MGI Symbol;Acc:MGI:3645000]
Tulp1	2.562624354	10.90352465	-1.96	0.043707214	0.9999452	1.36	Tulp1	tubby like protein 1 [Source:MGI Symbol;Acc:MGI:109571]
G0s2	286.6899159	1106.464006	-1.95	0.029713028	0.9999452	1.53	G0s2	G0/G1 switch gene 2 [Source:MGI Symbol;Acc:MGI:1316737]
Gm10073	46.57489951	177.5614809	-1.94	0.026878609	0.9999452	1.57	Gm10073	predicted pseudogene 10073 [Source:MGI Symbol;Acc:MGI:3641908]
Gm6485	11.15246808	44.79406113	-1.93	0.018939346	0.9999452	1.72	Gm6485	predicted gene 6485 [Source:MGI Symbol;Acc:MGI:3644007]
Emc9	90.74393713	342.4349102	-1.92	0.005012921	0.9999452	2.30	Emc9	ER membrane protein complex subunit 9 [Source:MGI Symbol;Acc:MGI:1934682]
Clec2d	529.4736734	1974.027425	-1.90	0.03795002	0.9999452	1.42	Clec2d	C-type lectin domain family 2, member d [Source:MGI Symbol;Acc:MGI:2135589]
Gm45629	8.923479015	30.43415832	-1.90	0.023531459	0.9999452	1.63	Gm45629	predicted gene 45629 [Source:MGI Symbol;Acc:MGI:5791465]
Pcbd2	40.88076213	151.976998	-1.88	0.008786629	0.9999452	2.06	Pcbd2	pterin 4 alpha carbinolamine dehydratase/dimerization cofactor of hepatocyte nuclear factor 1 alpha (TCF1) 2 [Source:N
Lag3	90.91864321	335.7738193	-1.88	0.034764075	0.9999452	1.46	Lag3	lymphocyte-activation gene 3 [Source:MGI Symbol;Acc:MGI:106588]
Gm11361	35.35494861	125.6516263	-1.87	0.003508854	0.9999452	2.45	Gm11361	predicted pseudogene 11361 [Source:MGI Symbol;Acc:MGI:3649931]
Cox6a1	1728.519574	6322.63148	-1.87	0.006467135	0.9999452	2.19	Cox6a1	cytochrome c oxidase subunit 6A1 [Source:MGI Symbol;Acc:MGI:103099]
Rpl35a	1249.524195	4547.208424	-1.86	0.011526552	0.9999452	1.94	Rpl35a	ribosomal protein L35A [Source:MGI Symbol;Acc:MGI:1928894]
Myl6	3927.689519	14288.93274	-1.86	0.012666823	0.9999452	1.90	Myl6	myosin, light polypeptide 6, alkali, smooth muscle and non-muscle [Source:MGI Symbol;Acc:MGI:109318]
Gm12251	33.34959281	115.0028174	-1.82	0.046677988	0.9999452	1.33	Gm12251	predicted gene 12251 [Source:MGI Symbol;Acc:MGI:3652216]
Dnph1	21.65759765	78.34909224	-1.82	0.009910376	0.9999452	2.00	Dnph1	2'-deoxynucleoside 5'-phosphate N-hydrolase 1 [Source:MGI Symbol;Acc:MGI:3039376]
Ddt	61.52570058	215.7522239	-1.82	0.018280524	0.9999452	1.74	Ddt	D-dopachrome tautomerase [Source:MGI Symbol;Acc:MGI:1298381]
Pgls	1158.35319	4081.358065	-1.82	0.015156261	0.9999452	1.82	Pgls	6-phosphogluconolactonase [Source:MGI Symbol;Acc:MGI:1913421]
Nhp2	353.389375	1237.346197	-1.81	0.017860314	0.9999452	1.75	Nhp2	NHP2 ribonucleoprotein [Source:MGI Symbol;Acc:MGI:1098547]
Dnaaf2	9.047464214	33.89698311	-1.81	0.032561586	0.9999452	1.49	Dnaaf2	dynein, axonemal assembly factor 2 [Source:MGI Symbol;Acc:MGI:1923566]
Rps15a-ps6	11.29132752	40.14820736	-1.80	0.023379003	0.9999452	1.63	Rps15a-ps6	ribosomal protein S15A, pseudogene 6 [Source:MGI Symbol;Acc:MGI:3650298]
Ppp1r14a	77.74764235	270.6268837	-1.80	0.031341854	0.9999452	1.50	Ppp1r14a	protein phosphatase 1, regulatory inhibitor subunit 14A [Source:MGI Symbol;Acc:MGI:1931139]
Smpd3	213.0707676	741.7267419	-1.80	0.044261671	0.9999452	1.35	Smpd3	sphingomyelin phosphodiesterase 3, neutral [Source:MGI Symbol;Acc:MGI:1927578]
Ppie	175.8940941	606.2537025	-1.80	0.019638201	0.9999452	1.71	Ppie	peptidylprolyl isomerase E (cyclophilin E) [Source:MGI Symbol;Acc:MGI:1917118]
Zfp688	95.21733173	328.0916264	-1.79	0.016463111	0.9999452	1.78	Zfp688	zinc finger protein 688 [Source:MGI Symbol;Acc:MGI:1916484]
Gm8319	3.449101717	13.32815984	-1.77	0.023819445	0.9999452	1.62	Gm8319	predicted gene 8319 [Source:MGI Symbol;Acc:MGI:3646693]
Ap2s1	1025.116604	3472.362677	-1.76	0.016967264	0.9999452	1.77	Ap2s1	adaptor-related protein complex 2, sigma 1 subunit [Source:MGI Symbol;Acc:MGI:2141861]
Hint2	185.6288535	621.3742672	-1.76	0.038043781	0.9999452	1.42	Hint2	histidine triad nucleotide binding protein 2 [Source:MGI Symbol;Acc:MGI:1916167]
2410015M20R	332.7130007	1116.893872	-1.75	0.018493482	0.9999452	1.73	2410015M20Ri	RIKEN cDNA 2410015M20 gene [Source:MGI Symbol;Acc:MGI:2442174]
Zbtb11os1	34.2886748	118.6782116	-1.75	0.014736806	0.9999452	1.83	Zbtb11os1	zinc finger and BTB domain containing 11, opposite strand 1 [Source:MGI Symbol;Acc:MGI:1913641]
Gm7846	28.05803183	91.46494794	-1.74	0.039019834	0.9999452	1.41	Gm7846	predicted gene 7846 [Source:MGI Symbol;Acc:MGI:3645212]
Mien1	57.277759	187.092659	-1.73	0.025156223	0.9999452	1.60	Mien1	migration and invasion enhancer 1 [Source:MGI Symbol;Acc:MGI:1913678]
Epop	54.36457387	183.7657026	-1.73	0.019356432	0.9999452	1.71	Epop	elongin BC and polycomb repressive complex 2 associated protein [Source:MGI Symbol;Acc:MGI:2143991]
Ptgis	153.2552383	508.9743534	-1.73	0.027828903	0.9999452	1.56	Ptgis	prostaglandin I2 (prostacyclin) synthase [Source:MGI Symbol;Acc:MGI:1097156]
Alyref2	14.84003176	52.08981838	-1.72	0.007287507	0.9999452	2.14	Alyref2	Aly/REF export factor 2 [Source:MGI Symbol;Acc:MGI:1913144]
Pfdn6	254.8056438	827.7326448	-1.71	0.04541789	0.9999452	1.34	Pfdn6	prefoldin subunit 6 [Source:MGI Symbol;Acc:MGI:95908]
Gm34084	10.76221321	37.16652926	-1.71	0.042035247	0.9999452	1.38	Gm34084	predicted gene, 34084 [Source:MGI Symbol;Acc:MGI:5593243]

4931413K12Ril	24.20196896	80.13866387	-1.71	0.041064284	0.9999452	1.39	4931413K12Ril	RIKEN cDNA 4931413K12 gene [Source:MGI Symbol;Acc:MGI:1918237]
Dhrs13	29.42658609	98.59678905	-1.70	0.027773571	0.9999452	1.56	Dhrs13	dehydrogenase/reductase (SDR family) member 13 [Source:MGI Symbol;Acc:MGI:1917701]
Rogdi	313.4787364	1011.409615	-1.69	0.042329097	0.9999452	1.37	Rogdi	rogdi homolog [Source:MGI Symbol;Acc:MGI:1913299]
Edf1	1191.497864	3806.993271	-1.68	0.049083723	0.9999452	1.31	Edf1	endothelial differentiation-related factor 1 [Source:MGI Symbol;Acc:MGI:1891227]
Mecr	261.1787249	832.4031423	-1.68	0.022800114	0.9999452	1.64	Mecr	mitochondrial trans-2-enoyl-CoA reductase [Source:MGI Symbol;Acc:MGI:1349441]
Sec61g	280.474585	890.4365674	-1.67	0.030008006	0.9999452	1.52	Sec61g	SEC61, gamma subunit [Source:MGI Symbol;Acc:MGI:1202066]
Gm7600	12.80640931	37.53782813	-1.67	0.049970237	0.9999452	1.30	Gm7600	predicted gene 7600 [Source:MGI Symbol;Acc:MGI:3647502]
Nat9	203.6345403	643.7536566	-1.67	0.033040744	0.9999452	1.48	Nat9	N-acetyltransferase 9 (GCN5-related, putative) [Source:MGI Symbol;Acc:MGI:1913426]
Rpl37	2090.508269	6634.531592	-1.67	0.041715182	0.9999452	1.38	Rpl37	ribosomal protein L37 [Source:MGI Symbol;Acc:MGI:1914531]
Shf	74.06962481	237.911868	-1.67	0.033378298	0.9999452	1.48	Shf	Src homology 2 domain containing F [Source:MGI Symbol;Acc:MGI:3613669]
Ccdc12	375.1599676	1182.725384	-1.66	0.040721192	0.9999452	1.39	Ccdc12	coiled-coil domain containing 12 [Source:MGI Symbol;Acc:MGI:1919904]
Zfp969	9.335854667	31.47687638	-1.66	0.040836524	0.9999452	1.39	Zfp969	zinc finger protein 969 [Source:MGI Symbol;Acc:MGI:3782422]
Tmem120a	631.9057005	1986.846638	-1.65	0.016820182	0.9999452	1.77	Tmem120a	transmembrane protein 120A [Source:MGI Symbol;Acc:MGI:2686991]
Gm10288	29.89995517	88.134106	-1.64	0.037170434	0.9999452	1.43	Gm10288	predicted gene 10288 [Source:MGI Symbol;Acc:MGI:3704227]
Gm47175	6.292795229	20.89365416	-1.64	0.049648326	0.9999452	1.30	Gm47175	predicted gene, 47175 [Source:MGI Symbol;Acc:MGI:6095958]
Trmt112-ps2	45.40849039	144.7156445	-1.64	0.018123508	0.9999452	1.74	Trmt112-ps2	tRNA methyltransferase 11-2, pseudogene 2 [Source:MGI Symbol;Acc:MGI:3651720]
Gm4950	44.91791911	138.6034164	-1.64	0.031477219	0.9999452	1.50	Gm4950	predicted pseudogene 4950 [Source:MGI Symbol;Acc:MGI:3649015]
Cd177	42.08928259	133.2395607	-1.63	0.033167593	0.9999452	1.48	Cd177	CD177 antigen [Source:MGI Symbol;Acc:MGI:1916141]
Chchd6	133.3652706	409.2583424	-1.63	0.02879371	0.9999452	1.54	Chchd6	coiled-coil-helix-coiled-coil-helix domain containing 6 [Source:MGI Symbol;Acc:MGI:1913348]
H1fx	50.07646524	156.0868278	-1.61	0.018168366	0.9999452	1.74	H1fx	H1 histone family, member X [Source:MGI Symbol;Acc:MGI:2685307]
Mal2	10.21430676	33.5351279	-1.60	0.026929452	0.9999452	1.57	Mal2	mal, T cell differentiation protein 2 [Source:MGI Symbol;Acc:MGI:2146021]
2410006H16Ri	120.3286727	367.3562167	-1.60	0.018300848	0.9999452	1.74	2410006H16Ril	RIKEN cDNA 2410006H16 gene [Source:MGI Symbol;Acc:MGI:1916471]
Bscl2	1114.237306	3355.467404	-1.59	0.024147737	0.9999452	1.62	Bscl2	Berardinelli-Seip congenital lipodystrophy 2 (seipin) [Source:MGI Symbol;Acc:MGI:1298392]
Dcxr	139.4074572	420.1827808	-1.59	0.020425634	0.9999452	1.69	Dcxr	dicarbonyl L-xylulose reductase [Source:MGI Symbol;Acc:MGI:1915130]
Acp5	1651.777293	4971.632985	-1.59	0.010235897	0.9999452	1.99	Acp5	acid phosphatase 5, tartrate resistant [Source:MGI Symbol;Acc:MGI:87883]
Psmb3	665.8297104	1988.838658	-1.58	0.011817077	0.9999452	1.93	Psmb3	proteasome (prosome, macropain) subunit, beta type 3 [Source:MGI Symbol;Acc:MGI:1347014]
9130401M01R	101.6492145	306.3427346	-1.58	0.01045486	0.9999452	1.98	9130401M01Ri	RIKEN cDNA 9130401M01 gene [Source:MGI Symbol;Acc:MGI:1923008]
Tmem205	266.4271825	781.957449	-1.56	0.030556932	0.9999452	1.51	Tmem205	transmembrane protein 205 [Source:MGI Symbol;Acc:MGI:3045495]
Psmg3	55.43207007	162.5064313	-1.56	0.039223823	0.9999452	1.41	Psmg3	proteasome (prosome, macropain) assembly chaperone 3 [Source:MGI Symbol;Acc:MGI:1913756]
Tfr2	20.54910957	63.25940399	-1.55	0.033534664	0.9999452	1.47	Tfr2	transferrin receptor 2 [Source:MGI Symbol;Acc:MGI:1354956]
S100b	101.8456821	301.3574116	-1.55	0.032654082	0.9999452	1.49	S100b	S100 protein, beta polypeptide, neural [Source:MGI Symbol;Acc:MGI:98217]
Tfpt	136.7203738	394.8414717	-1.54	0.025916641	0.9999452	1.59	Tfpt	TCF3 (E2A) fusion partner [Source:MGI Symbol;Acc:MGI:1916964]
Selenoh	170.0439467	494.6917331	-1.54	0.021404021	0.9999452	1.67	Selenoh	selenoprotein H [Source:MGI Symbol;Acc:MGI:1919907]
Cgref1	139.8623522	404.5617274	-1.53	0.040930777	0.9999452	1.39	Cgref1	cell growth regulator with EF hand domain 1 [Source:MGI Symbol;Acc:MGI:1915817]
Rnaseh2a	180.646881	521.3781064	-1.53	0.011956818	0.9999452	1.92	Rnaseh2a	ribonuclease H2, large subunit [Source:MGI Symbol;Acc:MGI:1916974]
Ppp1r35	166.6214909	476.8341368	-1.53	0.048716825	0.9999452	1.31	Ppp1r35	protein phosphatase 1, regulatory subunit 35 [Source:MGI Symbol;Acc:MGI:1922853]
Ndufaf2	75.17123276	211.6434451	-1.52	0.019488852	0.9999452	1.71	Ndufaf2	NADH:ubiquinone oxidoreductase complex assembly factor 2 [Source:MGI Symbol;Acc:MGI:1922847]
Ndufaf3	106.2867832	302.1969903	-1.51	0.009845519	0.9999452	2.01	Ndufaf3	NADH:ubiquinone oxidoreductase complex assembly factor 3 [Source:MGI Symbol;Acc:MGI:1913956]
Rps16	2907.916908	8266.310358	-1.51	0.035968034	0.9999452	1.44	Rps16	ribosomal protein S16 [Source:MGI Symbol;Acc:MGI:98118]
Pemt	29.2309986	83.2199857	-1.51	0.015074336	0.9999452	1.82	Pemt	phosphatidylethanolamine N-methyltransferase [Source:MGI Symbol;Acc:MGI:104535]
Gm10146	74.93241133	210.6358239	-1.50	0.020525143	0.9999452	1.69	Gm10146	predicted gene 10146 [Source:MGI Symbol;Acc:MGI:3704367]
Tspo	1039.377313	2939.324129	-1.50	0.01144269	0.9999452	1.94	Tspo	translocator protein [Source:MGI Symbol;Acc:MGI:88222]
Cdv3-ps	5.617028146	17.32740937	-1.50	0.044076674	0.9999452	1.36	Cdv3-ps	Cdv3 retrotransposed pseudogene [Source:MGI Symbol;Acc:MGI:3646628]
Hdhd3	22.82740022	67.98156665	-1.50	0.012859403	0.9999452	1.89	Hdhd3	haloacid dehalogenase-like hydrolase domain containing 3 [Source:MGI Symbol;Acc:MGI:1919998]
Gm3235	18.03584698	52.872739	-1.49	0.024530078	0.9999452	1.61	Gm3235	predicted gene 3235 [Source:MGI Symbol;Acc:MGI:3781413]
Isoc2a	353.2279904	988.9886485	-1.49	0.028884957	0.9999452	1.54	Isoc2a	isochorismatase domain containing 2a [Source:MGI Symbol;Acc:MGI:3609243]
Gm7658	14.76827569	41.66136989	-1.49	0.0217954	0.9999452	1.66	Gm7658	predicted gene 7658 [Source:MGI Symbol;Acc:MGI:3648029]
Nutf2-ps1	14.98527587	43.71094363	-1.48	0.026454588	0.9999452	1.58	Nutf2-ps1	nuclear transport factor 2, pseudogene 1 [Source:MGI Symbol;Acc:MGI:108008]
Cela1	81.63053346	227.5230693	-1.48	0.026173507	0.9999452	1.58	Cela1	chymotrypsin-like elastase family, member 1 [Source:MGI Symbol;Acc:MGI:95314]
Cldn5	995.1914774	2775.714812	-1.48	0.032836914	0.9999452	1.48	Cldn5	claudin 5 [Source:MGI Symbol;Acc:MGI:1276112]
Ifi35	264.6844063	736.8186568	-1.48	0.049254903	0.9999452	1.31	Ifi35	interferon-induced protein 35 [Source:MGI Symbol;Acc:MGI:1917360]
Bcl7c	453.3089223	1252.263464	-1.47	0.031535659	0.9999452	1.50	Bcl7c	B cell CLL/lymphoma 7C [Source:MGI Symbol;Acc:MGI:1332237]
Polr2k	46.95743891	123.7649155	-1.47	0.040763081	0.9999452	1.39	Polr2k	polymerase (RNA) II (DNA directed) polypeptide K [Source:MGI Symbol;Acc:MGI:102725]
Cox8a	1318.896152	3642.718194	-1.47	0.024138294	0.9999452	1.62	Cox8a	cytochrome c oxidase subunit 8A [Source:MGI Symbol;Acc:MGI:105959]
Ift27	169.4521684	467.3007578	-1.46	0.021720552	0.9999452	1.66	Ift27	intraflagellar transport 27 [Source:MGI Symbol;Acc:MGI:1914292]
Alad	269.843939	737.4741998	-1.46	0.049565601	0.9999452	1.30	Alad	aminolevulinate, delta-, dehydratase [Source:MGI Symbol;Acc:MGI:96853]
Mdp1	373.7492641	1022.138117	-1.45	0.02497103	0.9999452	1.60	Mdp1	magnesium-dependent phosphatase 1 [Source:MGI Symbol;Acc:MGI:1915131]
Tbcb	383.8773786	1052.035273	-1.45	0.018837413	0.9999452	1.72	Tbcb	tubulin folding cofactor B [Source:MGI Symbol;Acc:MGI:1913661]
Kcnk2	39.30037231	110.4675417	-1.44	0.023324344	0.9999452	1.63	Kcnk2	potassium channel, subfamily K, member 2 [Source:MGI Symbol;Acc:MGI:109366]
Scrn2	77.80351968	207.8085985	-1.44	0.049542566	0.9999452	1.31	Scrn2	secernin 2 [Source:MGI Symbol;Acc:MGI:1343092]
Mrpl22	76.6404078	209.1519655	-1.44	0.010623684	0.9999452	1.97	Mrpl22	mitochondrial ribosomal protein L22 [Source:MGI Symbol;Acc:MGI:1333794]

Exosc4	242.7102504	651.6907608	-1.43	0.036009991	0.9999452	1.44	Exosc4	exosome component 4 [Source:MGI Symbol;Acc:MGI:1923576]
Ntm	12.5614384	36.01710921	-1.43	0.034064213	0.9999452	1.47	Ntm	neurotrimin [Source:MGI Symbol;Acc:MGI:2446259]
2310009A05Ri	42.39115618	113.4126419	-1.43	0.037808385	0.9999452	1.42	2310009A05Ril	RIKEN cDNA 2310009A05 gene [Source:MGI Symbol;Acc:MGI:1913614]
Use1	749.9672486	2011.842986	-1.43	0.046013478	0.9999452	1.34	Use1	unconventional SNARE in the ER 1 homolog (S. cerevisiae) [Source:MGI Symbol;Acc:MGI:1914273]
Unc93a2	8.227715416	23.94564133	-1.42	0.023007462	0.9999452	1.64	Unc93a2	unc-93 homolog A2 [Source:MGI Symbol;Acc:MGI:3712668]
Ifi27	1185.688427	3173.318973	-1.42	0.034617145	0.9999452	1.46	Ifi27	interferon, alpha-inducible protein 27 [Source:MGI Symbol;Acc:MGI:1277180]
Mettl1	185.9598192	497.2791976	-1.42	0.01731025	0.9999452	1.76	Mettl1	methyltransferase like 1 [Source:MGI Symbol;Acc:MGI:1339986]
Rpl11	3654.710146	9757.349788	-1.42	0.047565546	0.9999452	1.32	Rpl11	ribosomal protein L11 [Source:MGI Symbol;Acc:MGI:1914275]
Mrpl33	471.6476938	1246.607972	-1.41	0.04735758	0.9999452	1.32	Mrpl33	mitochondrial ribosomal protein L33 [Source:MGI Symbol;Acc:MGI:2137225]
2410022M11R	55.38972445	150.4636283	-1.40	0.031679397	0.9999452	1.50	2410022M11Ri	RIKEN cDNA 2410022M11 gene [Source:MGI Symbol;Acc:MGI:1917005]
Ubl5	496.8820151	1308.157613	-1.40	0.037565081	0.9999452	1.43	Ubl5	ubiquitin-like 5 [Source:MGI Symbol;Acc:MGI:1913427]
Gsta4	69.46467604	185.2692398	-1.40	0.037661875	0.9999452	1.42	Gsta4	glutathione S-transferase, alpha 4 [Source:MGI Symbol;Acc:MGI:1309515]
Lgals1	4943.358343	13001.21866	-1.40	0.012641898	0.9999452	1.90	Lgals1	lectin, galactose binding, soluble 1 [Source:MGI Symbol;Acc:MGI:96777]
Mcrip1	1104.75424	2893.394553	-1.39	0.031804877	0.9999452	1.50	Mcrip1	MAPK regulated corepressor interacting protein 1 [Source:MGI Symbol;Acc:MGI:2384752]
Gale	173.1715092	453.291859	-1.39	0.01488098	0.9999452	1.83	Gale	galactose-4-epimerase, UDP [Source:MGI Symbol;Acc:MGI:1921496]
Tpgs1	326.3924825	850.6340689	-1.39	0.025780823	0.9999452	1.59	Tpgs1	tubulin polyglutamylase complex subunit 1 [Source:MGI Symbol;Acc:MGI:106618]
Pgf	39.63303392	106.3079226	-1.38	0.027151711	0.9999452	1.57	Pgf	placental growth factor [Source:MGI Symbol;Acc:MGI:105095]
Rnf113a1	18.45553522	50.6029069	-1.38	0.038407463	0.9999452	1.42	Rnf113a1	ring finger protein 113A1 [Source:MGI Symbol;Acc:MGI:1917192]
Psmb6	1060.772927	2747.119324	-1.38	0.041189929	0.9999452	1.39	Psmb6	proteasome (prosome, macropain) subunit, beta type 6 [Source:MGI Symbol;Acc:MGI:104880]
Pradc1	94.28771612	238.9841409	-1.37	0.0257148	0.9999452	1.59	Pradc1	protease-associated domain containing 1 [Source:MGI Symbol;Acc:MGI:1920577]
Pop7	74.97781488	194.5979827	-1.36	0.022208796	0.9999452	1.65	Pop7	processing of precursor 7, ribonuclease P family, (S. cerevisiae) [Source:MGI Symbol;Acc:MGI:1921347]
Fah	291.567054	744.3634898	-1.35	0.038293033	0.9999452	1.42	Fah	fumarylacetoacetate hydrolase [Source:MGI Symbol;Acc:MGI:95482]
Supt3	89.90861418	228.6722035	-1.35	0.028272387	0.9999452	1.55	Supt3	suppressor of Ty 3 [Source:MGI Symbol;Acc:MGI:1923723]
Vps45	152.3589805	384.7266689	-1.34	0.028877996	0.9999452	1.54	Vps45	vacuolar protein sorting 45 [Source:MGI Symbol;Acc:MGI:891965]
Alkbh2	23.22597546	59.65878325	-1.34	0.000845024	0.9999452	3.07	Alkbh2	alkB homolog 2, alpha-ketoglutarate-dependent dioxygenase [Source:MGI Symbol;Acc:MGI:2141032]
AC130815.1	8.057932166	22.00927581	-1.34	0.047453942	0.9999452	1.32	AC130815.1	novel transcript
Psmg4	108.5230652	271.6070784	-1.34	0.049818157	0.9999452	1.30	Psmg4	proteasome (prosome, macropain) assembly chaperone 4 [Source:MGI Symbol;Acc:MGI:1916916]
Mzt2	93.55776618	237.594056	-1.33	0.037609411	0.9999452	1.42	Mzt2	mitotic spindle organizing protein 2 [Source:MGI Symbol;Acc:MGI:1922845]
Rpl23a-ps3	288.2162711	720.9346502	-1.33	0.045738061	0.9999452	1.34	Rpl23a-ps3	ribosomal protein L23A, pseudogene 3 [Source:MGI Symbol;Acc:MGI:3781353]
Gm17018	138.4821684	342.4198348	-1.32	0.030543164	0.9999452	1.52	Gm17018	predicted gene 17018 [Source:MGI Symbol;Acc:MGI:4820566]
Aamp	1075.635368	2671.907132	-1.31	0.030067196	0.9999452	1.52	Aamp	angio-associated migratory protein [Source:MGI Symbol;Acc:MGI:107809]
2900041M22R	15.99666741	41.81893865	-1.31	0.046226423	0.9999452	1.34	2900041M22Ri	RIKEN cDNA 2900041M22 gene [Source:MGI Symbol;Acc:MGI:1925653]
2310011J03Rik	380.4049274	941.259441	-1.31	0.025233266	0.9999452	1.60	2310011J03Rik	RIKEN cDNA 2310011J03 gene [Source:MGI Symbol;Acc:MGI:1913624]
Mrpl27	255.6232992	626.9735423	-1.31	0.034149785	0.9999452	1.47	Mrpl27	mitochondrial ribosomal protein L27 [Source:MGI Symbol;Acc:MGI:2137224]
Rpl27	2997.847559	7395.475591	-1.30	0.031377086	0.9999452	1.50	Rpl27	ribosomal protein L27 [Source:MGI Symbol;Acc:MGI:98036]
B9d2	128.1468276	313.5171544	-1.30	0.043842749	0.9999452	1.36	B9d2	B9 protein domain 2 [Source:MGI Symbol;Acc:MGI:2387643]
Fam71e1	31.20169038	73.83978957	-1.30	0.033536666	0.9999452	1.47	Fam71e1	family with sequence similarity 71, member E1 [Source:MGI Symbol;Acc:MGI:1922788]
Tmem42	40.47958267	98.69891412	-1.29	0.009662495	0.9999452	2.01	Tmem42	transmembrane protein 42 [Source:MGI Symbol;Acc:MGI:1277176]
Gm15387	40.27602311	99.88172981	-1.27	0.030008977	0.9999452	1.52	Gm15387	predicted gene 15387 [Source:MGI Symbol;Acc:MGI:3705374]
Psmb4	1091.820351	2639.544351	-1.27	0.035562327	0.9999452	1.45	Psmb4	proteasome (prosome, macropain) subunit, beta type 4 [Source:MGI Symbol;Acc:MGI:1098257]
Gm15590	6.77736999	18.26083663	-1.25	0.036705001	0.9999452	1.44	Gm15590	predicted gene 15590 [Source:MGI Symbol;Acc:MGI:3831433]
Gm5112	13.94899971	35.92198098	-1.25	0.038708545	0.9999452	1.41	Gm5112	predicted gene 5112 [Source:MGI Symbol;Acc:MGI:3779460]
4930480K23Ril	15.5853995	39.59039954	-1.23	0.042631611	0.9999452	1.37	4930480K23Ril	RIKEN cDNA 4930480K23 gene [Source:MGI Symbol;Acc:MGI:1922266]
Zfpl1	216.0078553	507.2614253	-1.23	0.01286854	0.9999452	1.89	Zfpl1	zinc finger like protein 1 [Source:MGI Symbol;Acc:MGI:1891017]
Fcf1	196.1611683	461.3123576	-1.23	0.03466302	0.9999452	1.46	Fcf1	FCF1 rRNA processing protein [Source:MGI Symbol;Acc:MGI:1920986]
BC031181	458.2856265	1072.820579	-1.23	0.036268666	0.9999452	1.44	BC031181	cDNA sequence BC031181 [Source:MGI Symbol;Acc:MGI:3039614]
Trappc2l	195.2281944	452.4127358	-1.22	0.036171133	0.9999452	1.44	Trappc2l	trafficking protein particle complex 2-like [Source:MGI Symbol;Acc:MGI:1916295]
1810010H24Ri	11.48348945	29.50149137	-1.21	0.031003186	0.9999452	1.51	1810010H24Ril	RIKEN cDNA 1810010H24 gene [Source:MGI Symbol;Acc:MGI:1916316]
Atg101	296.2035336	675.9697277	-1.20	0.046976353	0.9999452	1.33	Atg101	autophagy related 101 [Source:MGI Symbol;Acc:MGI:1915368]
Stx8	256.6820228	584.6693431	-1.19	0.027783225	0.9999452	1.56	Stx8	syntaxin 8 [Source:MGI Symbol;Acc:MGI:1890156]
1810037I17Rik	202.1388412	461.962551	-1.19	0.023131895	0.9999452	1.64	1810037I17Rik	RIKEN cDNA 1810037I17 gene [Source:MGI Symbol;Acc:MGI:1914954]
Tmem219	248.0588084	564.5057405	-1.18	0.026796093	0.9999452	1.57	Tmem219	transmembrane protein 219 [Source:MGI Symbol;Acc:MGI:1915992]
Nnmt	368.1080558	834.6853264	-1.18	0.043866712	0.9999452	1.36	Nnmt	nicotinamide N-methyltransferase [Source:MGI Symbol;Acc:MGI:1099443]
Tmem258	179.6710189	405.7429779	-1.18	0.017228765	0.9999452	1.76	Tmem258	transmembrane protein 258 [Source:MGI Symbol;Acc:MGI:1916288]
Svbp	259.647354	579.9501094	-1.17	0.043343111	0.9999452	1.36	Svbp	small vasohibin binding protein [Source:MGI Symbol;Acc:MGI:1916466]
Cenpx	189.4716924	417.7865232	-1.15	0.041263978	0.9999452	1.38	Cenpx	centromere protein X [Source:MGI Symbol;Acc:MGI:894324]
Gpx3	14882.30932	32824.99675	-1.14	0.041560117	0.9999452	1.38	Gpx3	glutathione peroxidase 3 [Source:MGI Symbol;Acc:MGI:105102]
Trnau1ap	245.3365401	539.0907862	-1.14	0.042738491	0.9999452	1.37	Trnau1ap	tRNA selenocysteine 1 associated protein 1 [Source:MGI Symbol;Acc:MGI:1919037]
Rundc3a	43.24180239	97.29002855	-1.14	0.048197698	0.9999452	1.32	Rundc3a	RUN domain containing 3A [Source:MGI Symbol;Acc:MGI:1858752]
Elp6	65.14059381	143.9393664	-1.13	0.044038648	0.9999452	1.36	Elp6	elongator acetyltransferase complex subunit 6 [Source:MGI Symbol;Acc:MGI:1919349]
Nrgn	61.89453572	134.0899114	-1.13	0.04804402	0.9999452	1.32	Nrgn	neurogranin [Source:MGI Symbol;Acc:MGI:1927184]

Zfp993	14.68855229	34.52755492	-1.13	0.047093634	0.9999452	1.33	Zfp993	zinc finger protein 993 [Source:MGI Symbol;Acc:MGI:3713585]
Gpx1	3952.430532	8541.367295	-1.11	0.041802661	0.9999452	1.38	Gpx1	glutathione peroxidase 1 [Source:MGI Symbol;Acc:MGI:104887]
Arpp19	660.760832	1423.208637	-1.11	0.036574702	0.9999452	1.44	Arpp19	cAMP-regulated phosphoprotein 19 [Source:MGI Symbol;Acc:MGI:1891691]
Gamt	171.579976	369.6509175	-1.11	0.049986416	0.9999452	1.30	Gamt	guanidinoacetate methyltransferase [Source:MGI Symbol;Acc:MGI:1098221]
Rpa2	182.8851002	391.1508847	-1.11	0.040721046	0.9999452	1.39	Rpa2	replication protein A2 [Source:MGI Symbol;Acc:MGI:1339939]
Rps4l	209.327569	448.8597239	-1.10	0.024562793	0.9999452	1.61	Rps4l	ribosomal protein S4-like [Source:MGI Symbol;Acc:MGI:1913434]
Atp9a	883.7646678	1894.002137	-1.10	0.046928978	0.9999452	1.33	Atp9a	ATPase, class II, type 9A [Source:MGI Symbol;Acc:MGI:1330826]
Lsm10	114.9095711	246.0677831	-1.09	0.043659975	0.9999452	1.36	Lsm10	U7 snRNP-specific Sm-like protein LSM10 [Source:MGI Symbol;Acc:MGI:2151045]
Rpp40	46.52484055	99.43932606	-1.09	0.024269854	0.9999452	1.61	Rpp40	ribonuclease P 40 subunit [Source:MGI Symbol;Acc:MGI:1346084]
Ap1s1	626.9933434	1325.591859	-1.08	0.02514316	0.9999452	1.60	Ap1s1	adaptor protein complex AP-1, sigma 1 [Source:MGI Symbol;Acc:MGI:1098244]
Cryzl2	128.5988041	269.788974	-1.05	0.008005646	0.9999452	2.10	Cryzl2	crystallin zeta like 2 [Source:MGI Symbol;Acc:MGI:2448516]
Gm4285	37.29552453	75.22575663	-1.05	0.016714389	0.9999452	1.78	Gm4285	predicted gene 4285 [Source:MGI Symbol;Acc:MGI:3782463]
Ubald2	321.8516236	665.4897623	-1.04	0.04668059	0.9999452	1.33	Ubald2	UBA-like domain containing 2 [Source:MGI Symbol;Acc:MGI:1914635]
2810402E24Ril	83.60492437	170.7251357	-1.04	0.015162438	0.9999452	1.82	2810402E24Rik	RIKEN cDNA 2810402E24 gene [Source:MGI Symbol;Acc:MGI:1913715]
Mfap1b	170.8034924	350.5309033	-1.04	0.029403496	0.9999452	1.53	Mfap1b	microfibrillar-associated protein 1B [Source:MGI Symbol;Acc:MGI:3694697]
Alkbh4	90.34982412	185.1327481	-1.04	0.044514151	0.9999452	1.35	Alkbh4	alkB homolog 4, lysine demethylase [Source:MGI Symbol;Acc:MGI:1919291]
Mvk	182.504801	371.3611919	-1.03	0.020399873	0.9999452	1.69	Mvk	mevalonate kinase [Source:MGI Symbol;Acc:MGI:107624]
Pwwp2b	154.8417761	316.6772199	-1.02	0.030152328	0.9999452	1.52	Pwwp2b	PWWP domain containing 2B [Source:MGI Symbol;Acc:MGI:2142008]
Il17rc	321.7972379	644.7400737	-1.00	0.047077961	0.9999452	1.33	Il17rc	interleukin 17 receptor C [Source:MGI Symbol;Acc:MGI:2159336]
Cdk5rap3	717.875121	1429.181748	-0.99	0.042590905	0.9999452	1.37	Cdk5rap3	CDK5 regulatory subunit associated protein 3 [Source:MGI Symbol;Acc:MGI:1933126]
Fndc10	147.0311573	293.4670803	-0.99	0.024208746	0.9999452	1.62	Fndc10	fibronectin type III domain containing 10 [Source:MGI Symbol;Acc:MGI:2444790]
Psma5	393.1126561	775.392165	-0.98	0.049483615	0.9999452	1.31	Psma5	proteasome (prosome, macropain) subunit, alpha type 5 [Source:MGI Symbol;Acc:MGI:1347009]
1110012L19Ril	91.13979341	180.5998014	-0.96	0.020200669	0.9999452	1.69	1110012L19Rik	RIKEN cDNA 1110012L19 gene [Source:MGI Symbol;Acc:MGI:1915868]
Cnpy2	644.1551596	1248.522212	-0.95	0.042857441	0.9999452	1.37	Cnpy2	canopy FGF signaling regulator 2 [Source:MGI Symbol;Acc:MGI:1928477]
Polr2h	104.3838284	204.1381947	-0.95	0.036827461	0.9999452	1.43	Polr2h	polymerase (RNA) II (DNA directed) polypeptide H [Source:MGI Symbol;Acc:MGI:2384309]
Npc2	3684.825585	7097.536175	-0.95	0.034281062	0.9999452	1.46	Npc2	NPC intracellular cholesterol transporter 2 [Source:MGI Symbol;Acc:MGI:1915213]
Tmem14c	631.7548462	1201.774065	-0.93	0.04890424	0.9999452	1.31	Tmem14c	transmembrane protein 14C [Source:MGI Symbol;Acc:MGI:1913404]
Nelfe	307.3525927	581.5160536	-0.92	0.040437714	0.9999452	1.39	Nelfe	negative elongation factor complex member E, Rdbp [Source:MGI Symbol;Acc:MGI:102744]
Gss	207.1116098	386.2134958	-0.90	0.046527475	0.9999452	1.33	Gss	glutathione synthetase [Source:MGI Symbol;Acc:MGI:95852]
Slc27a4	634.4984033	1185.095706	-0.90	0.042759417	0.9999452	1.37	Slc27a4	solute carrier family 27 (fatty acid transporter), member 4 [Source:MGI Symbol;Acc:MGI:1347347]
Fggy	73.62085453	139.6936246	-0.89	0.047543011	0.9999452	1.32	Fggy	FGGY carbohydrate kinase domain containing [Source:MGI Symbol;Acc:MGI:1922828]
Tsen2	100.960295	189.5530906	-0.88	0.01574399	0.9999452	1.80	Tsen2	tRNA splicing endonuclease subunit 2 [Source:MGI Symbol;Acc:MGI:2141599]
Akip1	111.4043625	202.1203801	-0.88	0.048460701	0.9999452	1.31	Akip1	A kinase (PRKA) interacting protein 1 [Source:MGI Symbol;Acc:MGI:3041226]
Rbm8a	434.8793484	797.4022588	-0.88	0.039224718	0.9999452	1.41	Rbm8a	RNA binding motif protein 8a [Source:MGI Symbol;Acc:MGI:1913129]
Spcs1	674.3176996	1210.241863	-0.84	0.047875241	0.9999452	1.32	Spcs1	signal peptidase complex subunit 1 homolog (S. cerevisiae) [Source:MGI Symbol;Acc:MGI:1916269]
Slc46a1	97.30526744	173.5159021	-0.81	0.021542444	0.9999452	1.67	Slc46a1	solute carrier family 46, member 1 [Source:MGI Symbol;Acc:MGI:1098733]
Nubpl	52.25532631	89.74797906	-0.80	0.044157604	0.9999452	1.35	Nubpl	nucleotide binding protein-like [Source:MGI Symbol;Acc:MGI:1924076]
Paqr7	243.9338342	422.2607126	-0.79	0.039520532	0.9999452	1.40	Paqr7	progestin and adipoQ receptor family member VII [Source:MGI Symbol;Acc:MGI:1919154]
Polr3h	121.0668367	206.753722	-0.75	0.020852182	0.9999452	1.68	Polr3h	polymerase (RNA) III (DNA directed) polypeptide H [Source:MGI Symbol;Acc:MGI:1926179]
Snapc2	190.8115786	316.3638177	-0.74	0.044663688	0.9999452	1.35	Snapc2	small nuclear RNA activating complex, polypeptide 2 [Source:MGI Symbol;Acc:MGI:1914861]
Ppia	3425.821606	5652.794793	-0.72	0.026692413	0.9999452	1.57	Ppia	peptidylprolyl isomerase A [Source:MGI Symbol;Acc:MGI:97749]
Zdhhc12	73.41395869	121.4439291	-0.71	0.046361456	0.9999452	1.33	Zdhhc12	zinc finger, DHHC domain containing 12 [Source:MGI Symbol;Acc:MGI:1913470]

** rows 4-75 = genes with increased expression in Mg2800; rows 77-408 = genes with decreased expression in Mg2800 synovial tissues, compared with Mg500.*
