# Supplementary material for: Magnesium Supplementation Modifies Arthritis Synovial and Splenic Transcriptomic Signatures Including Ferroptosis and Cell Senescence Biological Pathways
Source: Nutrients. 2024 Dec 9;16(23):4247. doi: 10.3390/nu16234247 (PMC11644223; doi:10.3390/nu16234247)
Supplement: Supplementary file 1 [file nutrients-16-04247-s001.zip › Supplementary Table S4. DEG in spleens from Mg2800 versus Mg500 diet mice..pdf]

Table S4. DEG in the spleens of mice on the Mg2800 versus Mg500 diets*.

gene_name	SP_Mg2800	SP_Mg500	log2FoldChange (Mg2800 vs M	pvalue	padj	-log10(pvalue)	gene_name	gene_description
**Increased expression in Mg2800 versus Mg500**								
6030460B20Rik	10.3867402	0.14105086	5.42	0.001298811	0.980354196	2.886454042	6030460B20Rik	RIKEN cDNA 6030460B20 gene [Source:MGI Symbol;Acc:MGI:1924974]
Gm15385	11.4189689	0.3057014	5.15	0.00045432	0.980354196	3.342638144	Gm15385	predicted gene 15385 [Source:MGI Symbol;Acc:MGI:3705807]
Cdh15	5.45537639	0	4.90	0.025987035	0.980354196	1.585243269	Cdh15	cadherin 15 [Source:MGI Symbol;Acc:MGI:106672]
Hs3st6	4.01461372	0	4.46	0.027632303	0.980354196	1.558582918	Hs3st6	heparan sulfate (glucosamine) 3-O-sulfotransferase 6 [Source:MGI Symbol;Acc:MGI:3580487]
Cela2a	5.27084199	12.4787376	4.44	0.011961883	0.980354196	1.92220045	Cela2a	chymotrypsin-like elastase family, member 2A [Source:MGI Symbol;Acc:MGI:95316]
Mageb3	4.77051427	0.14105086	4.30	0.016617653	0.980354196	1.779430314	Mageb3	melanoma antigen, family B, 3 [Source:MGI Symbol;Acc:MGI:105109]
Gm38122	4.76494138	0.14105086	4.30	0.039165319	0.980354196	1.407098332	Gm38122	predicted gene, 38122 [Source:MGI Symbol;Acc:MGI:5611350]
Gm17271	6.24060476	0.26609143	4.27	0.016198199	0.980354196	1.79053327	Gm17271	predicted gene, 17271 [Source:MGI Symbol;Acc:MGI:4936905]
Gm43201	3.47186779	0	4.24	0.0486301	0.980354196	1.313094837	Gm43201	predicted gene 43201 [Source:MGI Symbol;Acc:MGI:5663338]
Gm37799	6.10200679	0.27280709	4.23	0.008522906	0.980354196	2.069412301	Gm37799	predicted gene, 37799 [Source:MGI Symbol;Acc:MGI:5611027]
Gm38414	5.99537728	0.27368506	4.22	0.019837641	0.980354196	1.702509973	Gm38414	predicted gene, 38414 [Source:MGI Symbol;Acc:MGI:5621299]
3830422I06Rik	4.40111791	0.13304571	4.20	0.017394918	0.980354196	1.759577614	3830422I06Rik	RIKEN cDNA 3830422I06 gene [Source:MGI Symbol;Acc:MGI:1917958]
Gm43343	5.76196751	0.28589641	4.17	0.023847145	0.980354196	1.622563608	Gm43343	predicted gene 43343 [Source:MGI Symbol;Acc:MGI:5663480]
Rpl31-ps12	3.24680904	0	4.16	0.028923615	0.980354196	1.538747428	Rpl31-ps12	ribosomal protein L31, pseudogene 1 2 [Source:NCBI gene;Acc:665562]
Gm6450	3.99980627	0.13304571	4.04	0.026568633	0.980354196	1.57563079	Gm6450	predicted gene 6450 [Source:MGI Symbol;Acc:MGI:3648082]
Trbv4	5.23966834	0.27409658	4.03	0.017034598	0.980354196	1.768668111	Trbv4	T cell receptor beta, variable 10 [Source:MGI Symbol;Acc:MGI:98584]
Crabp1	3.81136905	0.1326342	3.97	0.034454118	0.980354196	1.462758863	Crabp1	cellular retinoic acid binding protein I [Source:MGI Symbol;Acc:MGI:88490]
Trav6-2	4.92988664	0.2652684	3.94	0.027200002	0.980354196	1.565431064	Trav6-2	T cell receptor alpha variable 6-2 [Source:MGI Symbol;Acc:MGI:3642602]
Gm572	3.60429584	0.13304571	3.91	0.032388316	0.980354196	1.489611632	Gm572	predicted gene 572 [Source:MGI Symbol;Acc:MGI:2685418]
Gm45471	3.50630532	0.13304571	3.86	0.040035871	0.980354196	1.397550719	Gm45471	predicted gene 45471 [Source:MGI Symbol;Acc:MGI:5791307]
Rpl29-ps2	4.51210122	0.27814731	3.79	0.017267524	0.980354196	1.762769932	Rpl29-ps2	ribosomal protein L29, pseudogene 2 [Source:MGI Symbol;Acc:MGI:3648624]
Gm16288	3.09956009	0.14922793	3.69	0.033878675	0.980354196	1.470073583	Gm16288	predicted gene 16288 [Source:MGI Symbol;Acc:MGI:3826608]
Gm2663	4.03694535	0.27409658	3.65	0.030778434	0.980354196	1.511753481	Gm2663	predicted gene 2663 [Source:MGI Symbol;Acc:MGI:3780832]
Tpt1-ps5	5.32234502	0.42720332	3.63	0.019840906	0.980354196	1.7024385	Tpt1-ps5	tumor protein, translationally-controlled, pseudogene 5 [Source:MGI Symbol;Acc:MGI:2664999]
Cntf	10.1951094	0.83706122	3.60	0.001311558	0.980354196	2.882212499	Cntf	ciliary neurotrophic factor [Source:MGI Symbol;Acc:MGI:88439]
Hist1h4a	6.94663028	0.6114028	3.55	0.023218246	0.980354196	1.634170592	Hist1h4a	histone cluster 1, H4a [Source:MGI Symbol;Acc:MGI:2448419]
Gm48600	7.77259184	0.663171	3.52	0.023406952	0.980354196	1.630655135	Gm48600	predicted gene, 48600 [Source:MGI Symbol;Acc:MGI:6098178]
Gm37294	2.71084142	0.13304571	3.49	0.041447228	0.980354196	1.38250451	Gm37294	predicted gene, 37294 [Source:MGI Symbol;Acc:MGI:5610522]
Gng13	6.29103455	0.57179282	3.47	0.030872008	0.980354196	1.510435122	Gng13	guanine nucleotide binding protein (G protein), gamma 13 [Source:MGI Symbol;Acc:MGI:1925616]
Syt5	15.2646859	1.42907481	3.42	8.83E-05	0.808222723	4.054039296	Syt5	synaptotagmin V [Source:MGI Symbol;Acc:MGI:1926368]
Gm45483	8.88403556	0.8277159	3.42	0.016654525	0.980354196	1.778467749	Gm45483	predicted gene 45483 [Source:MGI Symbol;Acc:MGI:5791319]
Cda	5.84994629	7.72137941	3.38	0.03204383	0.980354196	1.494255581	Cda	cytidine deaminase [Source:MGI Symbol;Acc:MGI:1919519]
Cel	5.85036684	8.82708586	3.38	0.022188173	0.980354196	1.653878457	Cel	carboxyl ester lipase [Source:MGI Symbol;Acc:MGI:88374]
Bpifa6	8.52088327	0.8362382	3.34	0.009491698	0.980354196	2.022656088	Bpifa6	BPI fold containing family A, member 6 [Source:MGI Symbol;Acc:MGI:3647736]
A830005F24Rik	10.1377896	1.00674525	3.34	9.95E-05	0.808222723	4.002176919	A830005F24Rik	RIKEN cDNA A830005F24 gene [Source:MGI Symbol;Acc:MGI:3045249]
Baiap2l2	8.317905	0.84630517	3.30	0.012914598	0.980354196	1.888919108	Baiap2l2	BAI1-associated protein 2-like 2 [Source:MGI Symbol;Acc:MGI:2652819]
Gm5514	3.06342549	0.3057014	3.25	0.049474804	0.980354196	1.305615918	Gm5514	predicted gene 5514 [Source:MGI Symbol;Acc:MGI:3645435]
Gm43868	6.39476588	0.66399403	3.23	0.026021124	0.980354196	1.584673948	Gm43868	predicted gene, 43868 [Source:MGI Symbol;Acc:MGI:5690260]
Gm17281	5.320118	0.56799814	3.23	0.034138969	0.980354196	1.466749599	Gm17281	predicted gene, 17281 [Source:MGI Symbol;Acc:MGI:4936915]
Gm44702	6.03195298	0.6930387	3.10	0.022984846	0.980354196	1.638558402	Gm44702	predicted gene 44702 [Source:MGI Symbol;Acc:MGI:5753278]
Csf2	4.63062662	0.53977649	3.09	0.044101332	0.980354196	1.355548293	Csf2	colony stimulating factor 2 (granulocyte-macrophage) [Source:MGI Symbol;Acc:MGI:1339752]
Gm15694	5.87472617	0.70104385	3.07	0.019686485	0.980354196	1.70583182	Gm15694	predicted gene 15694 [Source:MGI Symbol;Acc:MGI:3783135]
Nudt8	9.04973628	1.06990177	3.06	0.018305823	0.980354196	1.737410741	Nudt8	nudix (nucleoside diphosphate linked moiety X)-type motif 8 [Source:MGI Symbol;Acc:MGI:1913637]
AC126942.2	5.4429865	0.70525431	2.95	0.044905777	0.980354196	1.347697785	AC126942.2	novel transcript
Gm30292	6.98923955	0.94131831	2.87	0.046874431	0.980354196	1.329063991	Gm30292	predicted gene, 30292 [Source:MGI Symbol;Acc:MGI:5589451]
Gm23969	13.1252341	1.84001178	2.84	0.001055396	0.980354196	2.976584556	Gm23969	predicted gene, 23969 [Source:MGI Symbol;Acc:MGI:5453746]
Gm12919	4.91499556	0.69624107	2.80	0.01204267	0.980354196	1.919277214	Gm12919	predicted gene 12919 [Source:MGI Symbol;Acc:MGI:3652001]
Hspa1a	964.829666	145.131159	2.73	0.011726401	0.980354196	1.930835259	Hspa1a	heat shock protein 1A [Source:MGI Symbol;Acc:MGI:96244]
Gm43024	5.53322771	0.83029487	2.72	0.038620929	0.980354196	1.413177284	Gm43024	predicted gene 43024 [Source:MGI Symbol;Acc:MGI:5663161]
Gm15536	6.43099424	0.98091897	2.71	0.026834008	0.980354196	1.571314455	Gm15536	predicted gene 15536 [Source:MGI Symbol;Acc:MGI:3782984]
Gemin4	10.6299608	1.65472898	2.67	0.018486797	0.980354196	1.733138328	Gemin4	gem nuclear organelle associated protein 4 [Source:MGI Symbol;Acc:MGI:2449313]
Rpl30-ps3	12.3732034	2.00517442	2.63	0.000414648	0.980354196	3.382320425	Rpl30-ps3	ribosomal protein L30, pseudogene 3 [Source:MGI Symbol;Acc:MGI:1321401]
Cadps	5.91031055	0.96076161	2.62	0.045748317	0.980354196	1.339624878	Cadps	Ca2+-dependent secretion activator [Source:MGI Symbol;Acc:MGI:1350922]
Gm14862	3.38669241	0.55157633	2.61	0.044907801	0.980354196	1.347678211	Gm14862	predicted gene 14862 [Source:MGI Symbol;Acc:MGI:3705234]
2700016F22Rik	9.63891798	1.61470573	2.59	0.040328955	0.980354196	1.394383031	2700016F22Rik	RIKEN cDNA 2700016F22 gene [Source:MGI Symbol;Acc:MGI:1919807]
P4ha3	16.2059412	2.843155	2.52	0.015745973	0.980354196	1.802830498	P4ha3	procollagen-proline, 2-oxoglutarate 4-dioxygenase (proline 4-hydroxylase), alpha polypeptide III [Source:MGI Sym
Gm29724	8.03169042	1.40967708	2.51	0.049348167	0.980354196	1.306728974	Gm29724	predicted gene, 29724 [Source:MGI Symbol;Acc:MGI:5588883]
Hspa1b	2245.43538	406.8557	2.46	0.016328103	0.980354196	1.787064269	Hspa1b	heat shock protein 1B [Source:MGI Symbol;Acc:MGI:99517]
Gm12260	6.90109462	1.26984618	2.45	0.038773105	0.980354196	1.411469419	Gm12260	predicted gene 12260 [Source:MGI Symbol;Acc:MGI:3651326]
Gm6218	5.70541149	1.09879099	2.36	0.030734538	0.980354196	1.512373311	Gm6218	predicted gene 6218 [Source:MGI Symbol;Acc:MGI:3643171]
Gm47283	108.862522	21.3602059	2.35	0.026727469	0.980354196	1.573042166	Gm47283	predicted gene, 47283 [Source:MGI Symbol;Acc:MGI:6096131]
Gm17823	15.1236204	2.98318025	2.33	0.010288268	0.980354196	1.987657731	Gm17823	predicted gene, 17823 [Source:MGI Symbol;Acc:MGI:5010008]
Tmed6	6.38572519	1.29091531	2.31	0.036696971	0.980354196	1.435369781	Tmed6	transmembrane p24 trafficking protein 6 [Source:MGI Symbol;Acc:MGI:1913519]
Gm20540	11.3372427	2.27454843	2.31	0.031912544	0.980354196	1.496038573	Gm20540	predicted gene 20540 [Source:MGI Symbol;Acc:MGI:5142005]
Tent5b	12.3720937	2.52737739	2.29	0.026919685	0.980354196	1.569930026	Tent5b	terminal nucleotidyltransferase 5B [Source:MGI Symbol;Acc:MGI:2140500]
Gm37716	29.5607873	6.25447837	2.24	0.008477777	0.980354196	2.071718011	Gm37716	predicted gene, 37716 [Source:MGI Symbol;Acc:MGI:5610944]
Gm38037	29.2720768	6.3788618	2.19	0.01531251	0.980354196	1.814953615	Gm38037	predicted gene, 38037 [Source:MGI Symbol;Acc:MGI:5611265]
Rps12-ps10	11.7606485	2.8006053	2.06	0.03139901	0.980354196	1.503084045	Rps12-ps10	ribosomal protein S12, pseudogene 10 [Source:MGI Symbol;Acc:MGI:3649508]
Gm29560	16.0383529	3.88555737	2.04	0.024444624	0.980354196	1.611816639	Gm29560	predicted gene 29560 [Source:MGI Symbol;Acc:MGI:5580266]
Hist1h1a	19.6505808	4.75059898	2.04	0.014968217	0.980354196	1.824829929	Hist1h1a	histone cluster 1, H1a [Source:MGI Symbol;Acc:MGI:1931523]
Olfr166	16.8499109	4.14785254	2.03	0.021172856	0.980354196	1.674220556	Olfr166	olfactory receptor 166 [Source:MGI Symbol;Acc:MGI:3030000]
Capn12	10.4219427	2.60687816	2.00	0.040381836	0.980354196	1.393813939	Capn12	calpain 12 [Source:MGI Symbol;Acc:MGI:1891369]
Rnf212	47.0344999	11.9468969	1.98	0.025068114	0.980354196	1.600878339	Rnf212	ring finger protein 212 [Source:MGI Symbol;Acc:MGI:3645767]
Gm15008	17.8717169	4.68267432	1.92	0.040375929	0.980354196	1.393877472	Gm15008	predicted gene 15008 [Source:MGI Symbol;Acc:MGI:3705107]
Gm32051	53.7635598	14.3198804	1.91	0.015148377	0.980354196	1.819633895	Gm32051	predicted gene, 32051 [Source:MGI Symbol;Acc:MGI:5591210]
Gm10575	13.3021807	3.55737588	1.89	0.03839435	0.980354196	1.41573268	Gm10575	predicted gene 10575 [Source:MGI Symbol;Acc:MGI:3708765]
Syngr4	22.4689741	6.12714363	1.87	0.019913508	0.980354196	1.700852227	Syngr4	synaptogyrin 4 [Source:MGI Symbol;Acc:MGI:1928903]

Gm7390	47.1026292	13.1663313	1.84	0.001402181	0.980354196	2.853195922	Gm7390	predicted gene 7390 [Source:MGI Symbol;Acc:MGI:3645154]
Guca2b	9.02268719	2.58830384	1.78	0.023980364	0.980354196	1.620144229	Guca2b	guanylate cyclase activator 2b (retina) [Source:MGI Symbol;Acc:MGI:1270851]
Ly6g6c	10.4603515	3.07386187	1.75	0.00930277	0.980354196	2.031387716	Ly6g6c	lymphocyte antigen 6 complex, locus G6C [Source:MGI Symbol;Acc:MGI:2148930]
Nags	63.1873941	19.4040842	1.70	0.032466174	0.980354196	1.488568888	Nags	N-acetylglutamate synthase [Source:MGI Symbol;Acc:MGI:2387600]
Gm26737	33.1744211	10.4721106	1.66	0.014850058	0.980354196	1.82827185	Gm26737	predicted gene, 26737 [Source:MGI Symbol;Acc:MGI:5477231]
Gm28707	19.5571302	6.21884086	1.65	0.029077419	0.980354196	1.536444145	Gm28707	predicted gene 28707 [Source:MGI Symbol;Acc:MGI:5579413]
4933417C20Rik	8.8094059	2.77150335	1.65	0.040686845	0.980354196	1.390545986	4933417C20Rik	RIKEN cDNA 4933417C20 gene [Source:MGI Symbol;Acc:MGI:1918378]
Fos	1489.83158	480.236122	1.63	0.031345989	0.980354196	1.503818023	Fos	FBJ osteosarcoma oncogene [Source:MGI Symbol;Acc:MGI:95574]
Rnf223	11.1441574	3.59457705	1.62	0.027872154	0.980354196	1.554829467	Rnf223	ring finger 223 [Source:MGI Symbol;Acc:MGI:3588193]
Erfe	49.2204088	16.2613603	1.60	0.048573621	0.980354196	1.31359952	Erfe	erythroferrone [Source:MGI Symbol;Acc:MGI:3606476]
Gm47196	26.0032948	8.8028547	1.56	0.01231058	0.980354196	1.909721485	Gm47196	predicted gene, 47196 [Source:MGI Symbol;Acc:MGI:6095990]
Fbxo40	27.0267237	9.27596501	1.55	0.016780189	0.980354196	1.775203152	Fbxo40	F-box protein 40 [Source:MGI Symbol;Acc:MGI:2443753]
Igkv3-7	534.894013	183.613985	1.54	0.013608442	0.980354196	1.866191593	Igkv3-7	immunoglobulin kappa variable 3-7 [Source:MGI Symbol;Acc:MGI:1330852]
Gm33023	14.8550596	5.27445931	1.50	0.046223281	0.980354196	1.335139231	Gm33023	predicted gene, 33023 [Source:MGI Symbol;Acc:MGI:5592182]
Hist1h2ac	31.3521012	11.13009	1.50	0.013578938	0.980354196	1.867134195	Hist1h2ac	histone cluster 1, H2ac [Source:MGI Symbol;Acc:MGI:2448287]
Fam110c	49.8012226	17.6318041	1.49	0.003080463	0.980354196	2.511384003	Fam110c	family with sequence similarity 110, member C [Source:MGI Symbol;Acc:MGI:1918813]
Gm38604	45.964785	16.3180311	1.49	0.04396285	0.980354196	1.356914161	Gm38604	predicted gene, 38604 [Source:MGI Symbol;Acc:MGI:5621489]
Hcn2	59.0117164	20.9261727	1.49	0.000553126	0.980354196	3.257175927	Hcn2	hyperpolarization-activated, cyclic nucleotide-gated K+ 2 [Source:MGI Symbol;Acc:MGI:1298210]
Il1bos	15.4070248	5.5134128	1.48	0.04774265	0.980354196	1.321093479	Il1bos	interleukin 1 beta, opposite strand [Source:MGI Symbol;Acc:MGI:3650458]
Tex15	26.8723446	9.58353973	1.47	0.012183989	0.980354196	1.914210502	Tex15	testis expressed gene 15 [Source:MGI Symbol;Acc:MGI:1934816]
Traj1	22.9723608	8.5675504	1.42	0.006718819	0.980354196	2.172707058	Traj1	T cell receptor alpha joining 1 [Source:MGI Symbol;Acc:MGI:4439841]
Gm5837	12.001229	4.50267325	1.41	0.026087967	0.980354196	1.583559764	Gm5837	predicted gene 5837 [Source:MGI Symbol;Acc:MGI:3648028]
Gm11405	11.1564383	4.22260275	1.39	0.020325101	0.980354196	1.691967288	Gm11405	predicted gene 11405 [Source:MGI Symbol;Acc:MGI:3651226]
Igkv3-4	1477.28124	567.099137	1.38	0.041606838	0.980354196	1.380835288	Igkv3-4	immunoglobulin kappa variable 3-4 [Source:MGI Symbol;Acc:MGI:1330855]
Gm16378	16.7674226	6.45320523	1.37	0.031524786	0.980354196	1.501347853	Gm16378	predicted gene 16378 [Source:MGI Symbol;Acc:MGI:3646886]
Rpl7a-ps3	20.4576151	7.85652574	1.37	0.008478159	0.980354196	2.071698443	Rpl7a-ps3	ribosomal protein L7A, pseudogene 3 [Source:MGI Symbol;Acc:MGI:3643413]
Apol11a	71.3033382	27.6731361	1.36	0.013905406	0.980354196	1.856816326	Apol11a	apolipoprotein L 11a [Source:MGI Symbol;Acc:MGI:3649094]
Hsph1	4016.19099	1566.34458	1.36	0.008073723	0.980354196	2.092926155	Hsph1	heat shock 105kDa/110kDa protein 1 [Source:MGI Symbol;Acc:MGI:105053]
Atf3	204.158197	80.378796	1.35	0.012824122	0.980354196	1.891972359	Atf3	activating transcription factor 3 [Source:MGI Symbol;Acc:MGI:109384]
4930438A08Rik	52.7050142	20.7015524	1.34	0.032607342	0.980354196	1.486684601	4930438A08Rik	RIKEN cDNA 4930438A08 gene [Source:MGI Symbol;Acc:MGI:1921238]
Apol10c-ps	44.2539866	17.6436759	1.32	0.045355565	0.980354196	1.343369419	Apol10c-ps	apolipoprotein L 10C, pseudogene [Source:MGI Symbol;Acc:MGI:3646982]
Gm6736	11.6861723	4.70891797	1.32	0.043068268	0.980354196	1.365842593	Gm6736	predicted gene 6736 [Source:MGI Symbol;Acc:MGI:3643048]
Igkv19-93	1300.1283	523.980067	1.31	0.046743964	0.980354196	1.330274461	Igkv19-93	immunoglobulin kappa chain variable 19-93 [Source:MGI Symbol;Acc:MGI:107617]
Gm5844	10.5898149	4.25394126	1.30	0.046392037	0.980354196	1.333556558	Gm5844	predicted gene 5844 [Source:MGI Symbol;Acc:MGI:3645252]
Gm10254	94.0930002	38.346353	1.29	0.002441536	0.980354196	2.612336868	Gm10254	predicted gene 10254 [Source:MGI Symbol;Acc:MGI:3708673]
Gm9575	26.5570895	10.9111246	1.28	0.049282387	0.980354196	1.307308265	Gm9575	predicted gene 9575 [Source:MGI Symbol;Acc:MGI:3779984]
C130051F05Rik	42.284074	17.549264	1.27	0.021107731	0.980354196	1.675558449	C130051F05Rik	RIKEN cDNA C130051F05 gene [Source:MGI Symbol;Acc:MGI:2443856]
Gm12411	132.901503	55.7193172	1.25	0.016368653	0.980354196	1.785987058	Gm12411	predicted gene 12411 [Source:MGI Symbol;Acc:MGI:3649918]
Gm266	16.2979807	6.82445019	1.25	0.022649133	0.980354196	1.644948418	Gm266	predicted gene 266 [Source:MGI Symbol;Acc:MGI:2685112]
Rnf128	33.6750066	14.2959376	1.23	0.047438248	0.980354196	1.323871359	Rnf128	ring finger protein 128 [Source:MGI Symbol;Acc:MGI:1914139]
Hist1h2ao	40.8516271	17.4594698	1.22	0.034593152	0.980354196	1.461009868	Hist1h2ao	histone cluster 1, H2ao [Source:MGI Symbol;Acc:MGI:2448302]
Gm2614	44.4303897	19.2426929	1.21	0.001015761	0.980354196	2.993208466	Gm2614	predicted gene 2614 [Source:MGI Symbol;Acc:MGI:3780782]
Ttc6	28.5673476	12.4302336	1.20	0.009883269	0.980354196	2.005099384	Ttc6	tetratricopeptide repeat domain 6 [Source:MGI Symbol;Acc:MGI:2684915]
Gm35585	26.3816485	11.4260164	1.20	0.046603875	0.980354196	1.331577971	Gm35585	predicted gene, 35585 [Source:MGI Symbol;Acc:MGI:5594744]
Gm9108	37.3515384	16.3734774	1.19	0.001175168	0.980354196	2.929900043	Gm9108	predicted gene 9108 [Source:MGI Symbol;Acc:MGI:3648830]
Gm45353	23.9896866	10.5974298	1.17	0.009702648	0.980354196	2.013109724	Gm45353	predicted gene 45353 [Source:MGI Symbol;Acc:MGI:5791189]
Reep6	202.78616	90.0624152	1.17	0.009382257	0.980354196	2.027692675	Reep6	receptor accessory protein 6 [Source:MGI Symbol;Acc:MGI:1917585]
Prokr1	69.0349595	31.0335996	1.15	0.027892628	0.980354196	1.554510565	Prokr1	prokineticin receptor 1 [Source:MGI Symbol;Acc:MGI:1929676]
1110019D14Rik	55.220523	24.9221444	1.14	0.030256181	0.980354196	1.51918589	1110019D14Rik	RIKEN cDNA 1110019D14 gene [Source:MGI Symbol;Acc:MGI:1923561]
Srsf12	19.3609169	8.82919655	1.14	0.047299312	0.980354196	1.325145176	Srsf12	serine/arginine-rich splicing factor 12 [Source:MGI Symbol;Acc:MGI:2661424]
Gm36932	18.1634099	8.32704872	1.12	0.047167898	0.980354196	1.326353477	Gm36932	predicted gene, 36932 [Source:MGI Symbol;Acc:MGI:5610160]
Art4	610.232712	280.108146	1.12	0.00402839	0.980354196	2.394868491	Art4	ADP-ribosyltransferase 4 [Source:MGI Symbol;Acc:MGI:1202710]
Gm13669	13.7557951	6.30766341	1.12	0.049122473	0.980354196	1.308719777	Gm13669	predicted gene 13669 [Source:MGI Symbol;Acc:MGI:3651174]
Arhgef26	23.9151265	11.1585113	1.10	0.02546681	0.980354196	1.594025452	Arhgef26	Rho guanine nucleotide exchange factor (GEF) 26 [Source:MGI Symbol;Acc:MGI:1918053]
mt-Ts2	75.8718324	35.6512414	1.09	0.002251805	0.980354196	2.647469221	mt-Ts2	mitochondrially encoded tRNA serine 2 [Source:MGI Symbol;Acc:MGI:102474]
Gdf15	122.093371	57.3506738	1.09	0.04445268	0.980354196	1.352102051	Gdf15	growth differentiation factor 15 [Source:MGI Symbol;Acc:MGI:1346047]
A730036I17Rik	79.0314484	37.1388235	1.09	0.044648423	0.980354196	1.350193876	A730036I17Rik	RIKEN cDNA A730036I17 gene [Source:MGI Symbol;Acc:MGI:3041182]
Gm13160	37.1837714	17.4394164	1.08	0.041130333	0.980354196	1.385837774	Gm13160	predicted gene 13160 [Source:MGI Symbol;Acc:MGI:3649984]
4933431K14Rik	65.3250526	31.6111688	1.04	0.035475465	0.980354196	1.450071903	4933431K14Rik	RIKEN cDNA 4933431K14 gene [Source:MGI Symbol;Acc:MGI:1918545]
Gstm5	454.351366	221.837102	1.03	0.022834017	0.980354196	1.64141768	Gstm5	glutathione S-transferase, mu 5 [Source:MGI Symbol;Acc:MGI:1309466]
Comtd1	71.4269508	35.1123972	1.02	0.014911535	0.980354196	1.826477648	Comtd1	catechol-O-methyltransferase domain containing 1 [Source:MGI Symbol;Acc:MGI:1916406]
Gm8355	44.7063093	22.4511835	0.99	0.003579786	0.980354196	2.446142935	Gm8355	predicted pseudogene 8355 [Source:MGI Symbol;Acc:MGI:3645191]
Igkv4-57	542.488457	274.281367	0.98	0.02548254	0.980354196	1.593757285	Igkv4-57	immunoglobulin kappa variable 4-57 [Source:MGI Symbol;Acc:MGI:2685035]
1700102P08Rik	65.0788573	32.9780721	0.98	0.014997094	0.980354196	1.823992886	1700102P08Rik	RIKEN cDNA 1700102P08 gene [Source:MGI Symbol;Acc:MGI:2148248]
Noct	588.28841	298.383958	0.98	0.003201702	0.980354196	2.494619093	Noct	nocturnin [Source:MGI Symbol;Acc:MGI:109382]
Gm13237	30.482691	15.4315909	0.98	0.048228334	0.980354196	1.31669774	Gm13237	predicted gene 13237 [Source:MGI Symbol;Acc:MGI:3649924]
Plk1	1348.64705	686.121654	0.97	0.044550572	0.980354196	1.351146716	Plk1	polo like kinase 1 [Source:MGI Symbol;Acc:MGI:97621]
Gm10371	112.501843	57.2048932	0.97	0.03032287	0.980354196	1.518229696	Gm10371	predicted gene 10371 [Source:MGI Symbol;Acc:MGI:3642716]
Gm18194	120.572681	61.6694807	0.97	0.021638872	0.980354196	1.664765382	Gm18194	predicted gene, 18194 [Source:MGI Symbol;Acc:MGI:5010379]
Hsp90aa1	19482.9673	10016.8152	0.96	0.007919461	0.980354196	2.101304376	Hsp90aa1	heat shock protein 90, alpha (cytosolic), class A member 1 [Source:MGI Symbol;Acc:MGI:96250]
Ankrd55	49.218516	25.3416707	0.96	0.011316712	0.980354196	1.946279736	Ankrd55	ankyrin repeat domain 55 [Source:MGI Symbol;Acc:MGI:1924568]
Mcm10	845.797541	436.041082	0.96	0.019701043	0.980354196	1.705510781	Mcm10	minichromosome maintenance 10 replication initiation factor [Source:MGI Symbol;Acc:MGI:1917274]
Gm2541	36.9393759	19.0721687	0.95	0.04901227	0.980354196	1.309695183	Gm2541	predicted pseudogene 2541 [Source:MGI Symbol;Acc:MGI:3780709]
Cdc45	641.371656	333.687435	0.94	0.015111682	0.980354196	1.820687194	Cdc45	cell division cycle 45 [Source:MGI Symbol;Acc:MGI:1338073]
1810044D09Rik	26.9647547	14.0708966	0.93	0.038393341	0.980354196	1.415744094	1810044D09Rik	RIKEN cDNA 1810044D09 gene [Source:MGI Symbol;Acc:MGI:1917048]
Teddm2	19.5768355	10.1908359	0.93	0.038596453	0.980354196	1.413452605	Teddm2	transmembrane epididymal family member 2 [Source:MGI Symbol;Acc:MGI:1923273]
Gm9625	25.6578598	13.5128546	0.92	0.041177153	0.980354196	1.385343684	Gm9625	predicted gene 9625 [Source:MGI Symbol;Acc:MGI:3780033]
Trbv17	49.1263778	26.1178942	0.91	0.039692644	0.980354196	1.401289971	Trbv17	T cell receptor beta, variable 17 [Source:MGI Symbol;Acc:MGI:98610]
Rfx2	673.369064	360.007231	0.90	0.013870243	0.980354196	1.85791593	Rfx2	regulatory factor X, 2 (influences HLA class II expression) [Source:MGI Symbol;Acc:MGI:106583]
Trem2	32.8920798	17.6466178	0.89	0.031118855	0.980354196	1.506976391	Trem2	triggering receptor expressed on myeloid cells 2 [Source:MGI Symbol;Acc:MGI:1913150]

Rundc3a	189.988372	102.657507	0.88	0.00074703	0.980354196	3.126661957	Rundc3a	RUN domain containing 3A [Source:MGI Symbol;Acc:MGI:1858752]
Isg20	3480.48219	1891.4571	0.88	0.011743202	0.980354196	1.930213469	Isg20	interferon-stimulated protein [Source:MGI Symbol;Acc:MGI:1928895]
Gm13408	69.1323597	37.5709602	0.88	0.006011154	0.980354196	2.221042146	Gm13408	predicted gene 13408 [Source:MGI Symbol;Acc:MGI:3649613]
Mcpt4	50.6943444	27.7249214	0.87	0.040459433	0.980354196	1.392980208	Mcpt4	mast cell protease 4 [Source:MGI Symbol;Acc:MGI:96940]
Snca	5485.57788	3010.34138	0.87	0.010424284	0.980354196	1.981953765	Snca	synuclein, alpha [Source:MGI Symbol;Acc:MGI:1277151]
Rad51c	187.373122	103.176474	0.86	0.037189402	0.980354196	1.429580805	Rad51c	RAD51 paralog C [Source:MGI Symbol;Acc:MGI:2150020]
Rps27a-ps2	184.819575	102.031652	0.85	0.012728326	0.980354196	1.89522871	Rps27a-ps2	ribosomal protein S27A, pseudogene 2 [Source:MGI Symbol;Acc:MGI:3643614]
Sh3yl1	201.052435	111.085019	0.85	0.043808125	0.980354196	1.358445334	Sh3yl1	Sh3 domain YSC-like 1 [Source:MGI Symbol;Acc:MGI:1346118]
Gm6274	66.0259101	36.4545411	0.85	0.02600128	0.980354196	1.585005272	Gm6274	predicted gene 6274 [Source:MGI Symbol;Acc:MGI:3645367]
Recql4	337.049731	187.052289	0.85	0.023146055	0.980354196	1.635523019	Recql4	RecQ protein-like 4 [Source:MGI Symbol;Acc:MGI:1931028]
Aldh1a1	2047.23125	1138.79925	0.85	0.024323453	0.980354196	1.613974772	Aldh1a1	aldehyde dehydrogenase family 1, subfamily A1 [Source:MGI Symbol;Acc:MGI:1353450]
Hist2h4	56.051266	31.0347924	0.84	0.045963697	0.980354196	1.337585047	Hist2h4	histone cluster 2, H4 [Source:MGI Symbol;Acc:MGI:2140113]
Gm10243	110.152296	61.4999419	0.84	0.024613737	0.980354196	1.608822444	Gm10243	predicted gene 10243 [Source:MGI Symbol;Acc:MGI:3704266]
M1ap	120.722437	67.9046291	0.83	0.031734413	0.980354196	1.498469531	M1ap	meiosis 1 associated protein [Source:MGI Symbol;Acc:MGI:1315200]
Dhfr	700.333686	395.914686	0.82	0.022387639	0.980354196	1.649991705	Dhfr	dihydrofolate reductase [Source:MGI Symbol;Acc:MGI:94890]
Zfpm1	6001.30168	3399.48798	0.82	0.030498301	0.980354196	1.515724354	Zfpm1	zinc finger protein, multitype 1 [Source:MGI Symbol;Acc:MGI:1095400]
Gm12346	192.69641	109.174641	0.82	0.031899027	0.980354196	1.496222564	Gm12346	predicted gene 12346 [Source:MGI Symbol;Acc:MGI:3649810]
Hba-a1	274619.003	155895.73	0.82	0.025124423	0.980354196	1.599903903	Hba-a1	hemoglobin alpha, adult chain 1 [Source:MGI Symbol;Acc:MGI:96015]
Mybl2	1027.38074	583.50433	0.82	0.028771039	0.980354196	1.541044454	Mybl2	myeloblastosis oncogene-like 2 [Source:MGI Symbol;Acc:MGI:101785]
Chtf18	548.731314	312.566553	0.81	0.014887254	0.980354196	1.827185402	Chtf18	CTF18, chromosome transmission fidelity factor 18 [Source:MGI Symbol;Acc:MGI:2384887]
Gm4739	477.194551	272.893163	0.81	0.024705771	0.980354196	1.607201588	Gm4739	predicted gene 4739 [Source:MGI Symbol;Acc:MGI:3642984]
1810055G02Rik	134.425773	76.818528	0.80	0.029165592	0.980354196	1.535129204	1810055G02Rik	RIKEN cDNA 1810055G02 gene [Source:MGI Symbol;Acc:MGI:1919306]
Ccne2	645.871876	369.884275	0.80	0.045771919	0.980354196	1.339400879	Ccne2	cyclin E2 [Source:MGI Symbol;Acc:MGI:1329034]
Ttk	284.34703	162.784774	0.80	0.037190401	0.980354196	1.429569139	Ttk	Ttk protein kinase [Source:MGI Symbol;Acc:MGI:1194921]
Ube2o	6244.73721	3591.79975	0.80	0.016074212	0.980354196	1.793870308	Ube2o	ubiquitin-conjugating enzyme E2O [Source:MGI Symbol;Acc:MGI:2444266]
Nt5c3	3371.06551	1945.13156	0.79	0.044208142	0.980354196	1.354497737	Nt5c3	5'-nucleotidase, cytosolic III [Source:MGI Symbol;Acc:MGI:1927186]
Stip1	4766.77091	2754.08264	0.79	0.01131128	0.980354196	1.946488247	Stip1	stress-induced phosphoprotein 1 [Source:MGI Symbol;Acc:MGI:109130]
Gm17828	100.72395	58.4122403	0.78	0.011260645	0.980354196	1.948436733	Gm17828	predicted gene, 17828 [Source:MGI Symbol;Acc:MGI:5010013]
Ttll12	1737.97232	1016.93779	0.77	0.034605222	0.980354196	1.46085836	Ttll12	tubulin tyrosine ligase-like family, member 12 [Source:MGI Symbol;Acc:MGI:3039573]
Gm15542	281.917161	165.833504	0.77	0.015561503	0.980354196	1.807948459	Gm15542	predicted gene 15542 [Source:MGI Symbol;Acc:MGI:3782990]
Ggt7	55.3515887	32.5890479	0.76	0.031449165	0.980354196	1.502390881	Ggt7	gamma-glutamyltransferase 7 [Source:MGI Symbol;Acc:MGI:1913385]
Pif1	382.701963	225.754981	0.76	0.043827224	0.980354196	1.358256037	Pif1	PIF1 5'-to-3' DNA helicase [Source:MGI Symbol;Acc:MGI:2143057]
Gm27003	71.4919839	42.3580819	0.76	0.020129605	0.980354196	1.696164747	Gm27003	predicted gene, 27003 [Source:MGI Symbol;Acc:MGI:5504118]
Rgcc	784.436895	463.459998	0.76	0.044833355	0.980354196	1.34839876	Rgcc	regulator of cell cycle [Source:MGI Symbol;Acc:MGI:1913464]
Gm2810	52.2479613	31.0207893	0.75	0.01932355	0.980354196	1.713913085	Gm2810	predicted pseudogene 2810 [Source:MGI Symbol;Acc:MGI:3780980]
Ube2l6	8118.00037	4816.18155	0.75	0.01046659	0.980354196	1.980194788	Ube2l6	ubiquitin-conjugating enzyme E2L 6 [Source:MGI Symbol;Acc:MGI:1914500]
Pip5k1b	425.554546	252.504791	0.75	0.044608455	0.980354196	1.350582818	Pip5k1b	phosphatidylinositol-4-phosphate 5-kinase, type 1 beta [Source:MGI Symbol;Acc:MGI:107930]
Cdca7	1106.5206	657.202206	0.75	0.008809333	0.980354196	2.055056973	Cdca7	cell division cycle associated 7 [Source:MGI Symbol;Acc:MGI:1914203]
Odc1	8506.74589	5055.92977	0.75	0.048278827	0.980354196	1.31624329	Odc1	ornithine decarboxylase, structural 1 [Source:MGI Symbol;Acc:MGI:97402]
Fam213a	2782.35739	1658.29191	0.75	0.030676476	0.980354196	1.513194532	Fam213a	family with sequence similarity 213, member A [Source:MGI Symbol;Acc:MGI:1917814]
Cenpn	383.857938	230.260513	0.74	0.040574155	0.980354196	1.391750516	Cenpn	centromere protein N [Source:MGI Symbol;Acc:MGI:1919405]
Lyrm4	259.676726	156.311121	0.73	0.044215361	0.980354196	1.354426825	Lyrm4	LYR motif containing 4 [Source:MGI Symbol;Acc:MGI:2683538]
Ighg1	3024.28415	1824.95023	0.73	0.047017932	0.980354196	1.327736476	Ighg1	immunoglobulin heavy constant gamma 1 (G1m marker) [Source:MGI Symbol;Acc:MGI:96446]
Dnaja4	477.811273	288.317317	0.73	0.041004481	0.980354196	1.387168681	Dnaja4	DnaJ heat shock protein family (Hsp40) member A4 [Source:MGI Symbol;Acc:MGI:1927638]
Ddias	237.450833	143.213343	0.73	0.042908658	0.980354196	1.367455068	Ddias	DNA damage-induced apoptosis suppressor [Source:MGI Symbol;Acc:MGI:1921291]
Gm6807	178.926574	108.273851	0.72	0.014045666	0.980354196	1.852457663	Gm6807	predicted gene 6807 [Source:MGI Symbol;Acc:MGI:3649145]
Alas2	22695.2007	13756.9453	0.72	0.015760257	0.980354196	1.802436705	Alas2	aminolevulinic acid synthase 2, erythroid [Source:MGI Symbol;Acc:MGI:87990]
Gm12070	26.097858	15.8293455	0.72	0.038746723	0.980354196	1.411765022	Gm12070	predicted gene 12070 [Source:MGI Symbol;Acc:MGI:3650751]
Snrpert	134.838464	81.7653031	0.72	0.027236358	0.980354196	1.564850966	Snrpert	small nuclear ribonucleoprotein E, pseudogene [Source:MGI Symbol;Acc:MGI:3650419]
Uhrf1	2935.69927	1793.25133	0.71	0.04082502	0.980354196	1.389073594	Uhrf1	ubiquitin-like, containing PHD and RING finger domains, 1 [Source:MGI Symbol;Acc:MGI:1338889]
Clcn2	289.473138	176.592866	0.71	0.028879537	0.980354196	1.539409774	Clcn2	chloride channel, voltage-sensitive 2 [Source:MGI Symbol;Acc:MGI:105061]
Tmem86b	357.445357	218.471522	0.71	0.001765483	0.980354196	2.75313646	Tmem86b	transmembrane protein 86B [Source:MGI Symbol;Acc:MGI:1915505]
Cdr2	4520.14112	2770.34638	0.71	0.034544519	0.980354196	1.46162085	Cdr2	cerebellar degeneration-related 2 [Source:MGI Symbol;Acc:MGI:1100885]
Rps19bp1	320.055938	196.078399	0.71	0.005913097	0.980354196	2.228184997	Rps19bp1	ribosomal protein S19 binding protein 1 [Source:MGI Symbol;Acc:MGI:1913788]
Pask	217.984995	133.378008	0.71	0.018227998	0.980354196	1.739261028	Pask	PAS domain containing serine/threonine kinase [Source:MGI Symbol;Acc:MGI:2155936]
Fam210b	1522.97099	934.922753	0.70	0.032404665	0.980354196	1.489392464	Fam210b	family with sequence similarity 210, member B [Source:MGI Symbol;Acc:MGI:1914267]
Rpl19-ps1	30.0125139	18.4349477	0.70	0.046996865	0.980354196	1.327931111	Rpl19-ps1	ribosomal protein L19, pseudogene 1 [Source:MGI Symbol;Acc:MGI:3642668]
Ncaph	1024.15797	632.740846	0.69	0.040905624	0.980354196	1.388216978	Ncaph	non-SMC condensin I complex, subunit H [Source:MGI Symbol;Acc:MGI:2444777]
Wdr55	763.495657	472.510685	0.69	0.006065938	0.980354196	2.217102033	Wdr55	WD repeat domain 55 [Source:MGI Symbol;Acc:MGI:1915186]
Hagh	1987.01598	1230.53588	0.69	0.046951171	0.980354196	1.328353572	Hagh	hydroxyacyl glutathione hydrolase [Source:MGI Symbol;Acc:MGI:95745]
Trip13	206.938807	128.130556	0.69	0.027692609	0.980354196	1.557636126	Trip13	thyroid hormone receptor interactor 13 [Source:MGI Symbol;Acc:MGI:1916966]
Smim5	224.396971	139.376276	0.69	0.044810536	0.980354196	1.348619861	Smim5	small integral membrane protein 5 [Source:MGI Symbol;Acc:MGI:1913778]
Tedc2	327.928773	203.98634	0.68	0.007344974	0.980354196	2.134009737	Tedc2	tubulin epsilon and delta complex 2 [Source:MGI Symbol;Acc:MGI:1919266]
Gm14269	52.761716	32.7453951	0.68	0.034453763	0.980354196	1.462763338	Gm14269	predicted gene 14269 [Source:MGI Symbol;Acc:MGI:3650060]
Rpp38	113.654766	70.7079903	0.68	0.017166366	0.980354196	1.765321632	Rpp38	ribonuclease P/MRP 38 subunit [Source:MGI Symbol;Acc:MGI:2443607]
Gm7666	168.361176	105.510186	0.67	0.002819126	0.980354196	2.549885513	Gm7666	predicted pseudogene 7666 [Source:MGI Symbol;Acc:MGI:3648907]
Gpr83	86.9321432	54.5260989	0.67	0.047023265	0.980354196	1.327687219	Gpr83	G protein-coupled receptor 83 [Source:MGI Symbol;Acc:MGI:95712]
Gm14648	60.4479567	37.7875289	0.67	0.043546076	0.980354196	1.361050974	Gm14648	predicted gene 14648 [Source:MGI Symbol;Acc:MGI:3705508]
Slc6a20a	177.336644	110.981743	0.67	0.019416673	0.980354196	1.711825183	Slc6a20a	solute carrier family 6 (neurotransmitter transporter), member 20A [Source:MGI Symbol;Acc:MGI:2143217]
Dnajb1	2092.24738	1311.75415	0.67	0.026910293	0.980354196	1.570081574	Dnajb1	DnaJ heat shock protein family (Hsp40) member B1 [Source:MGI Symbol;Acc:MGI:1931874]
Pnpo	2106.29564	1321.24693	0.67	0.043224613	0.980354196	1.364268886	Pnpo	pyridoxine 5'-phosphate oxidase [Source:MGI Symbol;Acc:MGI:2144151]
Rpl3-ps2	30.9158153	19.3378173	0.67	0.043762798	0.980354196	1.358894919	Rpl3-ps2	ribosomal protein L3, pseudogene 2 [Source:MGI Symbol;Acc:MGI:3645345]
Fxn	425.993937	268.138604	0.67	0.045435682	0.980354196	1.342602949	Fxn	frataxin [Source:MGI Symbol;Acc:MGI:1096879]
Fkbp4	4155.47601	2618.41042	0.67	0.031997002	0.980354196	1.494890712	Fkbp4	FK506 binding protein 4 [Source:MGI Symbol;Acc:MGI:95543]
Sapcd2	122.412114	77.0380361	0.67	0.039712633	0.980354196	1.401071318	Sapcd2	suppressor APC domain containing 2 [Source:MGI Symbol;Acc:MGI:1919330]
Knstrn	696.081575	439.452659	0.66	0.033355753	0.980354196	1.476829251	Knstrn	kinetochore-localized astrin/SPAG5 binding [Source:MGI Symbol;Acc:MGI:1289298]
Abcg2	1134.88051	717.010759	0.66	0.049684298	0.980354196	1.303780842	Abcg2	ATP binding cassette subfamily G member 2 (Junior blood group) [Source:MGI Symbol;Acc:MGI:1347061]
Txnl4b	266.026865	168.218141	0.66	0.000636745	0.980354196	3.196034457	Txnl4b	thioredoxin-like 4B [Source:MGI Symbol;Acc:MGI:2443724]
Tent5c	9703.03789	6156.22132	0.66	0.038271477	0.980354196	1.417124777	Tent5c	terminal nucleotidyltransferase 5C [Source:MGI Symbol;Acc:MGI:1921895]
Pradc1	194.637013	123.724937	0.65	0.00123311	0.980354196	2.90899818	Pradc1	protease-associated domain containing 1 [Source:MGI Symbol;Acc:MGI:1920577]

Gm19680	44.3373012	28.0686816	0.65	0.037325648	0.980354196	1.427992644	Gm19680	predicted gene, 19680 [Source:MGI Symbol;Acc:MGI:5011865]
Pelo	159.530548	101.679019	0.65	0.02208347	0.980354196	1.655932684	Pelo	pelota mRNA surveillance and ribosome rescue factor [Source:MGI Symbol;Acc:MGI:2145154]
Fancf	132.62198	84.6985372	0.65	0.01735591	0.980354196	1.760552611	Fancf	Fanconi anemia, complementation group F [Source:MGI Symbol;Acc:MGI:3689889]
Tceal8	342.093824	218.581097	0.64	0.043900001	0.980354196	1.35753547	Tceal8	transcription elongation factor A (SII)-like 8 [Source:MGI Symbol;Acc:MGI:1913934]
Ier5l	164.329159	105.340139	0.64	0.009063703	0.980354196	2.042694334	Ier5l	immediate early response 5-like [Source:MGI Symbol;Acc:MGI:1919750]
Mkrn1	11104.0562	7132.69151	0.64	0.026188531	0.980354196	1.581888862	Mkrn1	makorin, ring finger protein, 1 [Source:MGI Symbol;Acc:MGI:1859353]
Mcm6	5050.50122	3250.86474	0.64	0.049515472	0.980354196	1.305259077	Mcm6	minichromosome maintenance complex component 6 [Source:MGI Symbol;Acc:MGI:1298227]
Dapk2	574.174539	369.426967	0.64	0.049014948	0.980354196	1.309671454	Dapk2	death-associated protein kinase 2 [Source:MGI Symbol;Acc:MGI:1341297]
Isca1	4808.04309	3103.31993	0.63	0.035193039	0.980354196	1.453543229	Isca1	iron-sulfur cluster assembly 1 [Source:MGI Symbol;Acc:MGI:1916296]
Fam83g	142.084572	91.5413748	0.63	0.017225035	0.980354196	1.763839887	Fam83g	family with sequence similarity 83, member G [Source:MGI Symbol;Acc:MGI:1916890]
Hspa8	45528.1227	29412.4302	0.63	0.030716065	0.980354196	1.512634422	Hspa8	heat shock protein 8 [Source:MGI Symbol;Acc:MGI:105384]
Prr11	489.265623	316.98049	0.63	0.046499994	0.980354196	1.332547103	Prr11	proline rich 11 [Source:MGI Symbol;Acc:MGI:2444496]
Nfil3	175.663482	114.019141	0.62	0.030516912	0.980354196	1.515459415	Nfil3	nuclear factor, interleukin 3, regulated [Source:MGI Symbol;Acc:MGI:109495]
Foxd2os	104.595218	68.0356536	0.62	0.005671807	0.980354196	2.246278556	Foxd2os	forkhead box D2, opposite strand [Source:MGI Symbol;Acc:MGI:2444065]
Ahi1	292.720098	191.280989	0.61	0.0204324	0.980354196	1.689680618	Ahi1	Abelson helper integration site 1 [Source:MGI Symbol;Acc:MGI:87971]
Mthfd2	1240.34919	813.065567	0.61	0.040997836	0.980354196	1.387239066	Mthfd2	methylenetetrahydrofolate dehydrogenase (NAD+ dependent), methenyltetrahydrofolate cyclohydrolase [Source
Dnaja1	4580.77463	3012.50352	0.60	0.040344646	0.980354196	1.394214091	Dnaja1	DnaJ heat shock protein family (Hsp40) member A1 [Source:MGI Symbol;Acc:MGI:1270129]
Pcyox1l	243.358643	160.091193	0.60	0.047112999	0.980354196	1.32685925	Pcyox1l	prenylcysteine oxidase 1 like [Source:MGI Symbol;Acc:MGI:3606062]
Mrpl35	470.395461	310.532396	0.60	0.020393912	0.980354196	1.690499459	Mrpl35	mitochondrial ribosomal protein L35 [Source:MGI Symbol;Acc:MGI:1913473]
Ercc6l	442.066988	292.683592	0.59	0.020075413	0.980354196	1.697335511	Ercc6l	excision repair cross-complementing rodent repair deficiency complementation group 6 like [Source:MGI Symbol;
Tipin	863.334536	572.386507	0.59	0.04930776	0.980354196	1.307084727	Tipin	timeless interacting protein [Source:MGI Symbol;Acc:MGI:1921571]
Bpgm	10134.9585	6741.47254	0.59	0.019797301	0.980354196	1.703394014	Bpgm	2,3-bisphosphoglycerate mutase [Source:MGI Symbol;Acc:MGI:1098242]
Ddx28	315.18172	209.778822	0.59	0.022055313	0.980354196	1.656486775	Ddx28	DEAD (Asp-Glu-Ala-Asp) box polypeptide 28 [Source:MGI Symbol;Acc:MGI:1919236]
Pole2	323.950174	216.210136	0.58	0.042708949	0.980354196	1.369481116	Pole2	polymerase (DNA directed), epsilon 2 (p59 subunit) [Source:MGI Symbol;Acc:MGI:1197514]
Kctd7	312.894036	209.251436	0.58	0.033648056	0.980354196	1.473040022	Kctd7	potassium channel tetramerisation domain containing 7 [Source:MGI Symbol;Acc:MGI:2442265]
5-Mar	2539.86389	1700.85333	0.58	0.033364278	0.980354196	1.476718269	5-Mar	membrane-associated ring finger (C3HC4) 5 [Source:MGI Symbol;Acc:MGI:1915207]
Cyp7b1	52.5871827	35.068816	0.58	0.048167235	0.980354196	1.317248283	Cyp7b1	cytochrome P450, family 7, subfamily b, polypeptide 1 [Source:MGI Symbol;Acc:MGI:104978]
Nasp	2309.55393	1547.81064	0.58	0.043176936	0.980354196	1.36474818	Nasp	nuclear autoantigenic sperm protein (histone-binding) [Source:MGI Symbol;Acc:MGI:1355328]
Gm29216	157.528634	105.674745	0.58	0.020566659	0.980354196	1.686836253	Gm29216	predicted gene 29216 [Source:MGI Symbol;Acc:MGI:5579922]
Gm32220	95.6098937	64.2321526	0.57	0.031122609	0.980354196	1.506924003	Gm32220	predicted gene, 32220 [Source:MGI Symbol;Acc:MGI:5591379]
Mcm4	3198.79661	2157.16176	0.57	0.039734309	0.980354196	1.400834335	Mcm4	minichromosome maintenance complex component 4 [Source:MGI Symbol;Acc:MGI:103199]
Ppil1	1013.55114	685.532938	0.56	0.028547833	0.980354196	1.544426852	Ppil1	peptidylprolyl isomerase (cyclophilin)-like 1 [Source:MGI Symbol;Acc:MGI:1916066]
Ahsa1	2450.15159	1662.29513	0.56	0.048290347	0.980354196	1.316139674	Ahsa1	AHA1, activator of heat shock protein ATPase 1 [Source:MGI Symbol;Acc:MGI:2387603]
Srm	1230.73055	835.58622	0.56	0.039372475	0.980354196	1.404807284	Srm	spermidine synthase [Source:MGI Symbol;Acc:MGI:102690]
Cish	198.93513	134.772284	0.56	0.02862391	0.980354196	1.543271042	Cish	cytokine inducible SH2-containing protein [Source:MGI Symbol;Acc:MGI:103159]
Crat	1513.32124	1027.7984	0.56	0.030465596	0.980354196	1.516190321	Crat	carnitine acetyltransferase [Source:MGI Symbol;Acc:MGI:109501]
Perm1	126.241272	85.6488618	0.56	0.037418765	0.980354196	1.42691055	Perm1	PPARGC1 and ESRR induced regulator, muscle 1 [Source:MGI Symbol;Acc:MGI:1921433]
Foxm1	850.528488	580.897386	0.55	0.048736203	0.980354196	1.312148309	Foxm1	forkhead box M1 [Source:MGI Symbol;Acc:MGI:1347487]
Slc25a13	165.806468	113.225505	0.54	0.027056686	0.980354196	1.567725398	Slc25a13	solute carrier family 25 (mitochondrial carrier, adenine nucleotide translocator), member 13 [Source:MGI Symbol
Bcorl1	519.609579	355.992818	0.54	0.045272679	0.980354196	1.344163805	Bcorl1	BCL6 co-repressor-like 1 [Source:MGI Symbol;Acc:MGI:2443910]
Gins4	705.150455	486.212754	0.54	0.023919407	0.980354196	1.621249591	Gins4	GINS complex subunit 4 (Sld5 homolog) [Source:MGI Symbol;Acc:MGI:1923847]
Cox19	323.179858	222.600796	0.54	0.01434449	0.980354196	1.843314888	Cox19	cytochrome c oxidase assembly protein 19 [Source:MGI Symbol;Acc:MGI:1915283]
Mthfd1	2148.01367	1488.06794	0.53	0.038654546	0.980354196	1.412799423	Mthfd1	methylenetetrahydrofolate dehydrogenase (NADP+ dependent), methenyltetrahydrofolate cyclohydrolase, formy
Psme3	5475.42457	3796.59387	0.53	0.023182582	0.980354196	1.634838195	Psme3	proteaseome (prosome, macropain) activator subunit 3 (PA28 gamma, Ki) [Source:MGI Symbol;Acc:MGI:1096366
Tubg1	640.423031	443.802691	0.53	0.039895633	0.980354196	1.39907464	Tubg1	tubulin, gamma 1 [Source:MGI Symbol;Acc:MGI:101834]
Fads2	337.027623	233.354606	0.53	0.012825386	0.980354196	1.891929555	Fads2	fatty acid desaturase 2 [Source:MGI Symbol;Acc:MGI:1930079]
Mrpl40	570.859361	397.015269	0.52	0.02257007	0.980354196	1.646467094	Mrpl40	mitochondrial ribosomal protein L40 [Source:MGI Symbol;Acc:MGI:1332635]
Aars	3025.10678	2106.37499	0.52	0.039651526	0.980354196	1.401740094	Aars	alanyl-tRNA synthetase [Source:MGI Symbol;Acc:MGI:2384560]
Agfg2	880.82768	613.393475	0.52	0.027946471	0.980354196	1.553673026	Agfg2	ArfGAP with FG repeats 2 [Source:MGI Symbol;Acc:MGI:2443267]
Fam220a	3671.48356	2569.13025	0.51	0.046011431	0.980354196	1.33713426	Fam220a	family with sequence similarity 220, member A [Source:MGI Symbol;Acc:MGI:1914488]
2700097O09Rik	194.887637	136.468344	0.51	0.045814962	0.980354196	1.338992669	2700097O09Rik	RIKEN cDNA 2700097O09 gene [Source:MGI Symbol;Acc:MGI:1919908]
Ptges3	3006.55796	2107.65375	0.51	0.045041294	0.980354196	1.346389141	Ptges3	prostaglandin E synthase 3 [Source:MGI Symbol;Acc:MGI:1929282]
Rbm14	1461.77436	1024.97957	0.51	0.016822903	0.980354196	1.774099059	Rbm14	RNA binding motif protein 14 [Source:MGI Symbol;Acc:MGI:1929092]
Gm11427	525.926149	368.756636	0.51	0.03478638	0.980354196	1.458590763	Gm11427	predicted gene 11427 [Source:MGI Symbol;Acc:MGI:3651774]
Mrpl47	280.865294	197.877636	0.50	0.039162107	0.980354196	1.40713395	Mrpl47	mitochondrial ribosomal protein L47 [Source:MGI Symbol;Acc:MGI:1921850]
Med7	314.242227	221.43417	0.50	0.010414177	0.980354196	1.982375045	Med7	mediator complex subunit 7 [Source:MGI Symbol;Acc:MGI:1913463]
Chordc1	1960.77299	1386.45924	0.50	0.048649561	0.980354196	1.312921074	Chordc1	cysteine and histidine-rich domain (CHORD)-containing, zinc-binding protein 1 [Source:MGI Symbol;Acc:MGI:1914
Vcp-rs	1740.03729	1233.27382	0.50	0.004726407	0.980354196	2.325468883	Vcp-rs	valosin containing protein, related sequence [Source:MGI Symbol;Acc:MGI:894298]
Xrcc2	222.017183	157.460049	0.49	0.028747811	0.980354196	1.541395219	Xrcc2	X-ray repair complementing defective repair in Chinese hamster cells 2 [Source:MGI Symbol;Acc:MGI:1927345]
Rph3al	151.507702	107.876309	0.49	0.003553222	0.980354196	2.449377658	Rph3al	rabphilin 3A-like (without C2 domains) [Source:MGI Symbol;Acc:MGI:1923492]
Rbbp8	948.982658	675.678988	0.49	0.018213805	0.980354196	1.739599317	Rbbp8	retinoblastoma binding protein 8, endonuclease [Source:MGI Symbol;Acc:MGI:2442995]
Haus7	334.332923	238.17235	0.49	0.021946798	0.980354196	1.658628834	Haus7	HAUS augmin-like complex, subunit 7 [Source:MGI Symbol;Acc:MGI:1920988]
mt-Atp6	276.071289	197.070469	0.49	0.031355654	0.980354196	1.503684137	mt-Atp6	mitochondrially encoded ATP synthase 6 [Source:MGI Symbol;Acc:MGI:99927]
mt-Co2	892.835805	636.678318	0.49	0.026647946	0.980354196	1.57433626	mt-Co2	mitochondrially encoded cytochrome c oxidase II [Source:MGI Symbol;Acc:MGI:102503]
Gm11847	493.155049	352.320149	0.48	0.028763304	0.980354196	1.541161229	Gm11847	predicted gene 11847 [Source:MGI Symbol;Acc:MGI:3651787]
Prob1	113.403982	81.3438356	0.48	0.048462826	0.980354196	1.314591265	Prob1	proline rich basic protein 1 [Source:MGI Symbol;Acc:MGI:2686460]
Fntb	522.999183	373.987045	0.48	0.024677365	0.980354196	1.607701215	Fntb	farnesyltransferase, CAAX box, beta [Source:MGI Symbol;Acc:MGI:1861305]
Ddx31	394.362814	282.163291	0.48	0.011198553	0.980354196	1.95083809	Ddx31	DEAD/H (Asp-Glu-Ala-Asp/His) box polypeptide 31 [Source:MGI Symbol;Acc:MGI:2682639]
Mocos	137.249724	98.1293215	0.48	0.048414204	0.980354196	1.315027204	Mocos	molybdenum cofactor sulfurase [Source:MGI Symbol;Acc:MGI:1915841]
Smyd2	211.90466	151.615884	0.48	0.028611599	0.980354196	1.54345787	Smyd2	SET and MYND domain containing 2 [Source:MGI Symbol;Acc:MGI:1915889]
Snx3	2988.41005	2149.87345	0.47	0.03900718	0.980354196	1.408855446	Snx3	sorting nexin 3 [Source:MGI Symbol;Acc:MGI:1860188]
Gm4737	247.052962	177.468161	0.47	0.021289187	0.980354196	1.671840923	Gm4737	predicted gene 4737 [Source:MGI Symbol;Acc:MGI:3643647]
Noa1	823.98098	593.547619	0.47	0.041120475	0.980354196	1.385941877	Noa1	nitric oxide associated 1 [Source:MGI Symbol;Acc:MGI:1914306]
Impdh2-ps	1016.4436	733.314809	0.47	0.035441119	0.980354196	1.450492574	Impdh2-ps	inosine monophosphate dehydrogenase 2, pseudogene [Source:MGI Symbol;Acc:MGI:3705743]
Sephs2	769.54137	555.636807	0.47	0.040430668	0.980354196	1.393289083	Sephs2	selenophosphate synthetase 2 [Source:MGI Symbol;Acc:MGI:108388]
Myc	1610.05301	1163.03815	0.47	0.00954207	0.980354196	2.020357402	Myc	myelocytomatosis oncogene [Source:MGI Symbol;Acc:MGI:97250]
Xrcc6	1122.34474	812.358115	0.47	0.026323366	0.980354196	1.579658578	Xrcc6	X-ray repair complementing defective repair in Chinese hamster cells 6 [Source:MGI Symbol;Acc:MGI:95606]
Dcaf12	4417.74101	3205.66908	0.46	0.035769885	0.980354196	1.446482456	Dcaf12	DDB1 and CUL4 associated factor 12 [Source:MGI Symbol;Acc:MGI:1916220]
Ppid	1714.84433	1244.34274	0.46	0.001330973	0.980354196	2.875830754	Ppid	peptidylprolyl isomerase D (cyclophilin D) [Source:MGI Symbol;Acc:MGI:1914988]
Vegfb	551.75157	401.251324	0.46	0.009095703	0.980354196	2.041163729	Vegfb	vascular endothelial growth factor B [Source:MGI Symbol;Acc:MGI:106199]

Apeh	2536.07412	1851.5686	0.45	0.04533313	0.980354196	1.343584294	Apeh	acylpeptide hydrolase [Source:MGI Symbol;Acc:MGI:88041]
Med26	384.48092	280.741728	0.45	0.000369853	0.980354196	3.431970854	Med26	mediator complex subunit 26 [Source:MGI Symbol;Acc:MGI:1917875]
Fcf1	704.235049	514.368625	0.45	0.048840968	0.980354196	1.311215737	Fcf1	FCF1 rRNA processing protein [Source:MGI Symbol;Acc:MGI:1920986]
Gars	2606.41828	1908.48737	0.45	0.034495912	0.980354196	1.462232369	Gars	glycyl-tRNA synthetase [Source:MGI Symbol;Acc:MGI:2449057]
Bbip1	475.673593	348.050592	0.45	0.027670291	0.980354196	1.557986273	Bbip1	BBSome interacting protein 1 [Source:MGI Symbol;Acc:MGI:1913610]
Tmem237	105.780762	77.3395805	0.45	0.043207538	0.980354196	1.364440479	Tmem237	transmembrane protein 237 [Source:MGI Symbol;Acc:MGI:2138365]
Ncbp1	2114.1595	1553.98572	0.44	0.022850491	0.980354196	1.641104464	Ncbp1	nuclear cap binding protein subunit 1 [Source:MGI Symbol;Acc:MGI:1891840]
Mthfd1l	1367.3716	1006.1323	0.44	0.017176367	0.980354196	1.765068689	Mthfd1l	methylenetetrahydrofolate dehydrogenase (NADP+ dependent) 1-like [Source:MGI Symbol;Acc:MGI:1924836]
Nelfe	805.429602	593.983602	0.44	0.044399338	0.980354196	1.352623505	Nelfe	negative elongation factor complex member E, Rdbp [Source:MGI Symbol;Acc:MGI:102744]
Gabarapl2	1406.51747	1037.46988	0.44	0.020019443	0.980354196	1.69854801	Gabarapl2	gamma-aminobutyric acid (GABA) A receptor-associated protein-like 2 [Source:MGI Symbol;Acc:MGI:1890602]
Hspa14	1422.3557	1049.86027	0.44	0.016934205	0.980354196	1.771235187	Hspa14	heat shock protein 14 [Source:MGI Symbol;Acc:MGI:1354164]
Vcpkmt	245.170385	181.14794	0.44	0.04418358	0.980354196	1.354739098	Vcpkmt	valosin containing protein lysine (K) methyltransferase [Source:MGI Symbol;Acc:MGI:2684917]
Elp5	673.508294	498.421253	0.43	0.030574217	0.980354196	1.514644656	Elp5	elongator acetyltransferase complex subunit 5 [Source:MGI Symbol;Acc:MGI:1859017]
Umps	933.746952	691.14701	0.43	0.046049022	0.980354196	1.336779589	Umps	uridine monophosphate synthetase [Source:MGI Symbol;Acc:MGI:1298388]
Babam2	804.524346	596.650082	0.43	0.049550983	0.980354196	1.304947725	Babam2	BRISC and BRCA1 A complex member 2 [Source:MGI Symbol;Acc:MGI:1333875]
Tmem70	543.541169	402.998561	0.43	0.013245888	0.980354196	1.877918921	Tmem70	transmembrane protein 70 [Source:MGI Symbol;Acc:MGI:1915068]
Lrrc14	644.757914	478.789346	0.43	0.02058214	0.980354196	1.686509472	Lrrc14	leucine rich repeat containing 14 [Source:MGI Symbol;Acc:MGI:2445060]
Nmnat3	217.0664	161.182714	0.43	0.04930268	0.980354196	1.307129473	Nmnat3	nicotinamide nucleotide adenylyltransferase 3 [Source:MGI Symbol;Acc:MGI:1921330]
Txnl4a	395.472126	295.26664	0.42	0.042308556	0.980354196	1.373571797	Txnl4a	thioredoxin-like 4A [Source:MGI Symbol;Acc:MGI:1351613]
Prpf3	905.013785	676.203827	0.42	0.00908777	0.980354196	2.041542673	Prpf3	pre-mRNA processing factor 3 [Source:MGI Symbol;Acc:MGI:1918017]
Pdzd11	396.139894	296.137632	0.42	0.035833005	0.980354196	1.44571677	Pdzd11	PDZ domain containing 11 [Source:MGI Symbol;Acc:MGI:1919871]
Ppp1r8	1137.7776	851.916792	0.42	0.037348258	0.980354196	1.42772965	Ppp1r8	protein phosphatase 1, regulatory subunit 8 [Source:MGI Symbol;Acc:MGI:2140494]
Ppp2r1b	1697.57548	1271.70783	0.42	0.035498404	0.980354196	1.449791172	Ppp2r1b	protein phosphatase 2, regulatory subunit A, beta [Source:MGI Symbol;Acc:MGI:1920949]
Cptp	259.471398	194.346609	0.41	0.030595051	0.980354196	1.514348819	Cptp	ceramide-1-phosphate transfer protein [Source:MGI Symbol;Acc:MGI:1933107]
Cinp	639.144808	479.735795	0.41	0.048085571	0.980354196	1.317985222	Cinp	cyclin-dependent kinase 2 interacting protein [Source:MGI Symbol;Acc:MGI:1914486]
Msh6	1699.31635	1276.40302	0.41	0.034688789	0.980354196	1.459810861	Msh6	mutS homolog 6 [Source:MGI Symbol;Acc:MGI:1343961]
Twnk	458.204127	344.377154	0.41	0.021631627	0.980354196	1.664910814	Twnk	twinkle mtDNA helicase [Source:MGI Symbol;Acc:MGI:2137410]
Trim27	2272.94557	1712.8869	0.41	0.020828023	0.980354196	1.681351951	Trim27	tripartite motif-containing 27 [Source:MGI Symbol;Acc:MGI:97904]
Eif2b1	678.837389	511.960034	0.41	0.031664313	0.980354196	1.49942993	Eif2b1	eukaryotic translation initiation factor 2B, subunit 1 (alpha) [Source:MGI Symbol;Acc:MGI:2384802]
Ccndbp1	2325.61682	1756.05592	0.40	0.030903774	0.980354196	1.509988481	Ccndbp1	cyclin D-type binding-protein 1 [Source:MGI Symbol;Acc:MGI:109595]
Tmem33	1149.40416	868.010463	0.40	0.045553683	0.980354196	1.341476505	Tmem33	transmembrane protein 33 [Source:MGI Symbol;Acc:MGI:1915128]
1700017B05Rik	1582.9288	1196.86806	0.40	0.032850624	0.980354196	1.483456377	1700017B05Rik	RIKEN cDNA 1700017B05 gene [Source:MGI Symbol;Acc:MGI:1921461]
Nudt5	768.972566	581.512002	0.40	0.019700515	0.980354196	1.705522421	Nudt5	nudix (nucleoside diphosphate linked moiety X)-type motif 5 [Source:MGI Symbol;Acc:MGI:1858232]
Armc5	923.453254	698.935672	0.40	0.001619647	0.980354196	2.790579629	Armc5	armadillo repeat containing 5 [Source:MGI Symbol;Acc:MGI:2384586]
Thap7	577.377922	438.339041	0.40	0.034643488	0.980354196	1.460378389	Thap7	THAP domain containing 7 [Source:MGI Symbol;Acc:MGI:1916259]
Pinx1	241.231424	183.21258	0.40	0.047568054	0.980354196	1.322684615	Pinx1	PIN2/TERF1 interacting, telomerase inhibitor 1 [Source:MGI Symbol;Acc:MGI:1919650]
Prmt5	1134.42931	861.798211	0.40	0.021037718	0.980354196	1.677001371	Prmt5	protein arginine N-methyltransferase 5 [Source:MGI Symbol;Acc:MGI:1351645]
Ppm1d	832.070625	632.071714	0.40	0.019142031	0.980354196	1.718011985	Ppm1d	protein phosphatase 1D magnesium-dependent, delta isoform [Source:MGI Symbol;Acc:MGI:1858214]
Brix1	836.540892	636.424811	0.39	0.045859415	0.980354196	1.33857149	Brix1	BRX1, biogenesis of ribosomes [Source:MGI Symbol;Acc:MGI:1915082]
Ubash3a	429.484575	327.05773	0.39	0.019365705	0.980354196	1.712966688	Ubash3a	ubiquitin associated and SH3 domain containing, A [Source:MGI Symbol;Acc:MGI:1926074]
Alg3	301.409303	229.283911	0.39	0.036532549	0.980354196	1.437320025	Alg3	asparagine-linked glycosylation 3 (alpha-1,3-mannosyltransferase) [Source:MGI Symbol;Acc:MGI:1098592]
Ydjc	163.102798	124.313013	0.39	0.025264095	0.980354196	1.597496254	Ydjc	YdjC homolog (bacterial) [Source:MGI Symbol;Acc:MGI:1916351]
Rae1	1433.39121	1092.68392	0.39	0.020763925	0.980354196	1.682690548	Rae1	ribonucleic acid export 1 [Source:MGI Symbol;Acc:MGI:1913929]
Gmps	1474.34548	1128.64265	0.39	0.041303553	0.980354196	1.384012588	Gmps	guanine monophosphate synthetase [Source:MGI Symbol;Acc:MGI:2448526]
Top1	3407.93565	2614.93658	0.38	0.044458082	0.980354196	1.352049277	Top1	topoisomerase (DNA) I [Source:MGI Symbol;Acc:MGI:98788]
Prcc	1451.64945	1116.04764	0.38	0.03409932	0.980354196	1.467254282	Prcc	papillary renal cell carcinoma (translocation-associated) [Source:MGI Symbol;Acc:MGI:2137738]
Gpbp1	2635.003	2027.64037	0.38	0.033531085	0.980354196	1.474552393	Gpbp1	GC-rich promoter binding protein 1 [Source:MGI Symbol;Acc:MGI:1920524]
Vkorc1l1	964.419859	745.256468	0.37	0.042269212	0.980354196	1.373975848	Vkorc1l1	vitamin K epoxide reductase complex, subunit 1-like 1 [Source:MGI Symbol;Acc:MGI:1916818]
Bora	434.263411	335.906511	0.37	0.039399752	0.980354196	1.404506512	Bora	bora, aurora kinase A activator [Source:MGI Symbol;Acc:MGI:1924994]
Sgf29	446.037911	345.467683	0.37	0.045134228	0.980354196	1.345493982	Sgf29	SAGA complex associated factor 29 [Source:MGI Symbol;Acc:MGI:1922815]
Kiz	393.062615	304.524293	0.37	0.013640868	0.980354196	1.865157994	Kiz	kizuna centrosomal protein [Source:MGI Symbol;Acc:MGI:2684960]
Ssbp2	333.742375	258.410032	0.37	0.020673229	0.980354196	1.684591685	Ssbp2	single-stranded DNA binding protein 2 [Source:MGI Symbol;Acc:MGI:1914220]
Lonp1	2060.85987	1600.97521	0.36	0.022976879	0.980354196	1.638708963	Lonp1	lon peptidase 1, mitochondrial [Source:MGI Symbol;Acc:MGI:1921392]
Cpt2	505.59215	392.334661	0.36	0.0135863	0.980354196	1.8668988	Cpt2	carnitine palmitoyltransferase 2 [Source:MGI Symbol;Acc:MGI:109176]
Mettl16	935.626814	728.777936	0.36	0.0095742	0.980354196	2.018897505	Mettl16	methyltransferase like 16 [Source:MGI Symbol;Acc:MGI:1914743]
Ints13	1251.41226	977.156672	0.36	0.032223146	0.980354196	1.491832061	Ints13	integrator complex subunit 13 [Source:MGI Symbol;Acc:MGI:1918427]
Wdr46	897.569493	701.622423	0.35	0.045240657	0.980354196	1.344471097	Wdr46	WD repeat domain 46 [Source:MGI Symbol;Acc:MGI:1931871]
Hs1bp3	334.667199	262.466804	0.35	0.006572042	0.980354196	2.18229967	Hs1bp3	HCLS1 binding protein 3 [Source:MGI Symbol;Acc:MGI:1913224]
Heatr3	773.935573	607.084462	0.35	0.019035467	0.980354196	1.720436464	Heatr3	HEAT repeat containing 3 [Source:MGI Symbol;Acc:MGI:2444491]
Mycbp	217.986049	171.285669	0.35	0.047008271	0.980354196	1.327825722	Mycbp	MYC binding protein [Source:MGI Symbol;Acc:MGI:1891750]
Asb3	408.846143	321.588949	0.35	0.044733566	0.980354196	1.34936648	Asb3	ankyrin repeat and SOCS box-containing 3 [Source:MGI Symbol;Acc:MGI:1929749]
Cdc5l	1968.89223	1550.98949	0.34	0.027066197	0.980354196	1.567572762	Cdc5l	cell division cycle 5-like (S. pombe) [Source:MGI Symbol;Acc:MGI:1918952]
Nolc1	2168.14891	1709.38942	0.34	0.033030896	0.980354196	1.481079645	Nolc1	nucleolar and coiled-body phosphoprotein 1 [Source:MGI Symbol;Acc:MGI:1918019]
Mcu	490.427828	386.892645	0.34	0.029584	0.980354196	1.528943106	Mcu	mitochondrial calcium uniporter [Source:MGI Symbol;Acc:MGI:3026965]
Helq	421.980332	333.347152	0.34	0.034164174	0.980354196	1.466429075	Helq	helicase, POLQ-like [Source:MGI Symbol;Acc:MGI:2176740]
Nsmce2	313.215363	247.293558	0.34	0.023139623	0.980354196	1.635643721	Nsmce2	NSE2/MMS21 homolog, SMC5-SMC6 complex SUMO ligase [Source:MGI Symbol;Acc:MGI:1915751]
Ptpn12	1029.43252	814.444157	0.34	0.020242332	0.980354196	1.693739456	Ptpn12	protein tyrosine phosphatase, non-receptor type 12 [Source:MGI Symbol;Acc:MGI:104673]
Gm13456	468.242231	371.042731	0.34	0.039929755	0.980354196	1.398703355	Gm13456	predicted gene 13456 [Source:MGI Symbol;Acc:MGI:3651389]
Uchl3	473.062368	374.656054	0.34	0.046958091	0.980354196	1.328289567	Uchl3	ubiquitin carboxyl-terminal esterase L3 (ubiquitin thiolesterase) [Source:MGI Symbol;Acc:MGI:1355274]
Gm6136	3787.82494	3003.66643	0.33	0.035042157	0.980354196	1.455409169	Gm6136	predicted pseudogene 6136 [Source:MGI Symbol;Acc:MGI:3646897]
Chst10	477.071445	378.26683	0.33	0.03754775	0.980354196	1.425416082	Chst10	carbohydrate sulfotransferase 10 [Source:MGI Symbol;Acc:MGI:2138283]
Tfg	985.114626	781.758145	0.33	0.007035918	0.980354196	2.152679231	Tfg	Trk-fused gene [Source:MGI Symbol;Acc:MGI:1338041]
Nelfa	862.352205	684.673109	0.33	0.010909701	0.980354196	1.962187152	Nelfa	negative elongation factor complex member A, Whsc2 [Source:MGI Symbol;Acc:MGI:1346098]
Psmd6	1506.87396	1196.46592	0.33	0.040680849	0.980354196	1.390609992	Psmd6	proteasome (prosome, macropain) 26S subunit, non-ATPase, 6 [Source:MGI Symbol;Acc:MGI:1913663]
Zdhhc6	940.275666	747.435698	0.33	0.025150925	0.980354196	1.599446038	Zdhhc6	zinc finger, DHHC domain containing 6 [Source:MGI Symbol;Acc:MGI:1914230]
Desi1	891.398403	710.334592	0.33	0.045928286	0.980354196	1.337919762	Desi1	desumoylating isopeptidase 1 [Source:MGI Symbol;Acc:MGI:106313]
Nufip1	427.108161	341.50257	0.32	0.034456452	0.980354196	1.462729444	Nufip1	nuclear fragile X mental retardation protein interacting protein 1 [Source:MGI Symbol;Acc:MGI:1351474]
Pak4	456.405676	365.844437	0.32	0.025949562	0.980354196	1.585869968	Pak4	p21 (RAC1) activated kinase 4 [Source:MGI Symbol;Acc:MGI:1917834]
Nup54	739.324363	593.664151	0.32	0.032229948	0.980354196	1.491740395	Nup54	nucleoporin 54 [Source:MGI Symbol;Acc:MGI:1920460]
Cdkn2aipnl	1500.42798	1206.43146	0.31	0.044826843	0.980354196	1.348461846	Cdkn2aipnl	CDKN2A interacting protein N-terminal like [Source:MGI Symbol;Acc:MGI:1261797]

Wbp11	2373.0651	1910.18573	0.31	0.021551771	0.980354196	1.666517036	Wbp11	WW domain binding protein 11 [Source:MGI Symbol;Acc:MGI:1891823]
Gtpbp4	1882.13179	1516.1677	0.31	0.028020736	0.980354196	1.552520462	Gtpbp4	GTP binding protein 4 [Source:MGI Symbol;Acc:MGI:1916487]
Stn1	324.265424	261.379121	0.31	0.030337399	0.980354196	1.518021657	Stn1	STN1, CST complex subunit [Source:MGI Symbol;Acc:MGI:1915581]
Rpf1	732.181394	591.446219	0.31	0.02222463	0.980354196	1.653165461	Rpf1	ribosome production factor 1 homolog [Source:MGI Symbol;Acc:MGI:1917535]
Gnl3	1246.05932	1010.98794	0.30	0.037047407	0.980354196	1.431242184	Gnl3	guanine nucleotide binding protein-like 3 (nucleolar) [Source:MGI Symbol;Acc:MGI:1353651]
Sfpq	8532.85152	6939.72902	0.30	0.047117921	0.980354196	1.32681388	Sfpq	splicing factor proline/glutamine rich (polypyrimidine tract binding protein associated) [Source:MGI Symbol;Acc:M
Rfx1	1398.87795	1138.42876	0.30	0.022494139	0.980354196	1.647930626	Rfx1	regulatory factor X, 1 (influences HLA class II expression) [Source:MGI Symbol;Acc:MGI:105982]
Rbm10	2029.62215	1650.90288	0.30	0.030571622	0.980354196	1.514681519	Rbm10	RNA binding motif protein 10 [Source:MGI Symbol;Acc:MGI:2384310]
Fam133b	574.816865	467.848561	0.30	0.041105825	0.980354196	1.386096631	Fam133b	family with sequence similarity 133, member B [Source:MGI Symbol;Acc:MGI:1915402]
Tra2b	3099.3772	2527.25456	0.29	0.034741282	0.980354196	1.45915416	Tra2b	transformer 2 beta [Source:MGI Symbol;Acc:MGI:106016]
Nup93	1066.72217	869.863884	0.29	0.019930082	0.980354196	1.700490914	Nup93	nucleoporin 93 [Source:MGI Symbol;Acc:MGI:1919055]
Rprd1b	1341.67594	1094.91667	0.29	0.045177464	0.980354196	1.345078151	Rprd1b	regulation of nuclear pre-mRNA domain containing 1B [Source:MGI Symbol;Acc:MGI:1917720]
Taf5l	1096.38099	895.109283	0.29	0.030180663	0.980354196	1.520271224	Taf5l	TATA-box binding protein associated factor 5 like [Source:MGI Symbol;Acc:MGI:1919039]
Slc25a46	964.74466	791.263203	0.29	0.005784715	0.980354196	2.237718033	Slc25a46	solute carrier family 25, member 46 [Source:MGI Symbol;Acc:MGI:1914703]
Vps53	881.917551	723.4182	0.28	0.023112885	0.980354196	1.636145842	Vps53	VPS53 GARP complex subunit [Source:MGI Symbol;Acc:MGI:1915549]
Hspa4	5307.23148	4358.87674	0.28	0.024909415	0.980354196	1.603636472	Hspa4	heat shock protein 4 [Source:MGI Symbol;Acc:MGI:1342292]
Cenpo	391.139238	321.041848	0.28	0.044314745	0.980354196	1.353451745	Cenpo	centromere protein O [Source:MGI Symbol;Acc:MGI:1923800]
Pold3	1308.7182	1074.9657	0.28	0.041650358	0.980354196	1.380381261	Pold3	polymerase (DNA-directed), delta 3, accessory subunit [Source:MGI Symbol;Acc:MGI:1915217]
Nop14	1024.10343	841.198537	0.28	0.029079684	0.980354196	1.536410317	Nop14	NOP14 nucleolar protein [Source:MGI Symbol;Acc:MGI:1922666]
Tra2a	2803.24955	2313.95605	0.28	0.038473742	0.980354196	1.414835572	Tra2a	transformer 2 alpha [Source:MGI Symbol;Acc:MGI:1933972]
Rrp12	765.098991	631.887022	0.28	0.018240446	0.980354196	1.738964547	Rrp12	ribosomal RNA processing 12 homolog (S. cerevisiae) [Source:MGI Symbol;Acc:MGI:2147437]
Rrp1	3931.70223	3246.69631	0.28	0.032623373	0.980354196	1.486471138	Rrp1	ribosomal RNA processing 1 homolog (S. cerevisiae) [Source:MGI Symbol;Acc:MGI:1203500]
Fbxl19	644.507364	531.871285	0.28	0.042630247	0.980354196	1.370282151	Fbxl19	F-box and leucine-rich repeat protein 19 [Source:MGI Symbol;Acc:MGI:3039600]
Trmt10c	543.301369	448.475317	0.28	0.038558351	0.980354196	1.413881548	Trmt10c	tRNA methyltransferase 10C [Source:MGI Symbol;Acc:MGI:1196261]
Sqle	410.596467	339.180991	0.27	0.041482249	0.980354196	1.382137706	Sqle	squalene epoxidase [Source:MGI Symbol;Acc:MGI:109296]
Prpf19	2491.13533	2060.60183	0.27	0.02826381	0.980354196	1.548769295	Prpf19	pre-mRNA processing factor 19 [Source:MGI Symbol;Acc:MGI:106247]
Eaf1	1558.55488	1290.79422	0.27	0.03916621	0.980354196	1.407088452	Eaf1	ELL associated factor 1 [Source:MGI Symbol;Acc:MGI:1921677]
Zfp251	580.590669	483.052321	0.27	0.040790649	0.980354196	1.389439385	Zfp251	zinc finger protein 251 [Source:MGI Symbol;Acc:MGI:1918841]
Pwp2	950.613798	793.939639	0.26	0.044104255	0.980354196	1.35551951	Pwp2	PWP2 periodic tryptophan protein homolog (yeast) [Source:MGI Symbol;Acc:MGI:1341200]
Terf2ip	632.144516	528.914932	0.26	0.037400861	0.980354196	1.4271184	Terf2ip	telomeric repeat binding factor 2, interacting protein [Source:MGI Symbol;Acc:MGI:1929871]
Cbx1	1429.4034	1197.06509	0.26	0.045864213	0.980354196	1.338526054	Cbx1	chromobox 1 [Source:MGI Symbol;Acc:MGI:105369]
Polr1b	857.031591	719.333328	0.25	0.028517436	0.980354196	1.544889524	Polr1b	polymerase (RNA) I polypeptide B [Source:MGI Symbol;Acc:MGI:108014]
Slc25a12	1120.33928	941.228887	0.25	0.039381252	0.980354196	1.404710481	Slc25a12	solute carrier family 25 (mitochondrial carrier, Aralar), member 12 [Source:MGI Symbol;Acc:MGI:1926080]
Rab35	1872.30462	1573.22358	0.25	0.042112894	0.980354196	1.375584913	Rab35	RAB35, member RAS oncogene family [Source:MGI Symbol;Acc:MGI:1924657]
Pelp1	1007.9913	847.823558	0.25	0.048238358	0.980354196	1.316607484	Pelp1	proline, glutamic acid and leucine rich protein 1 [Source:MGI Symbol;Acc:MGI:1922523]
Cstf3	744.620273	626.347546	0.25	0.021088853	0.980354196	1.67594704	Cstf3	cleavage stimulation factor, 3' pre-RNA, subunit 3 [Source:MGI Symbol;Acc:MGI:1351825]
Rbm18	593.7308	499.763062	0.25	0.047826677	0.980354196	1.320329793	Rbm18	RNA binding motif protein 18 [Source:MGI Symbol;Acc:MGI:1915139]
Gnl2	1193.44994	1007.72416	0.24	0.038053571	0.980354196	1.419604582	Gnl2	guanine nucleotide binding protein-like 2 (nucleolar) [Source:MGI Symbol;Acc:MGI:2385207]
Npepps	1592.50726	1345.12798	0.24	0.048710788	0.980354196	1.312374845	Npepps	aminopeptidase puromycin sensitive [Source:MGI Symbol;Acc:MGI:1101358]
Wrnip1	1035.15977	874.437367	0.24	0.03311223	0.980354196	1.48001157	Wrnip1	Werner helicase interacting protein 1 [Source:MGI Symbol;Acc:MGI:1926153]
Fto	1133.10119	958.411159	0.24	0.037600358	0.980354196	1.42480802	Fto	fat mass and obesity associated [Source:MGI Symbol;Acc:MGI:1347093]
Nol11	805.491615	683.43785	0.24	0.034368851	0.980354196	1.463834987	Nol11	nucleolar protein 11 [Source:MGI Symbol;Acc:MGI:1916229]
Inpp5b	1397.66011	1187.16543	0.23	0.009235469	0.980354196	2.034541045	Inpp5b	inositol polyphosphate-5-phosphatase B [Source:MGI Symbol;Acc:MGI:103257]
Eif3c	5871.22312	4995.35044	0.23	0.048696715	0.980354196	1.312500335	Eif3c	eukaryotic translation initiation factor 3, subunit C [Source:MGI Symbol;Acc:MGI:1926966]
Pnpt1	819.760682	697.238505	0.23	0.029226282	0.980354196	1.53422643	Pnpt1	polyribonucleotide nucleotidyltransferase 1 [Source:MGI Symbol;Acc:MGI:1918951]
Yipf5	594.780635	508.287377	0.22	0.022469186	0.980354196	1.648412661	Yipf5	Yip1 domain family, member 5 [Source:MGI Symbol;Acc:MGI:1914430]
Aida	1336.44209	1146.36114	0.22	0.033559024	0.980354196	1.474190678	Aida	axin interactor, dorsalization associated [Source:MGI Symbol;Acc:MGI:1919737]
Cstf1	914.970365	785.330415	0.22	0.042211554	0.980354196	1.374568659	Cstf1	cleavage stimulation factor, 3' pre-RNA, subunit 1 [Source:MGI Symbol;Acc:MGI:1914587]
Pgam1	1464.12332	1261.23925	0.21	0.036247867	0.980354196	1.440717544	Pgam1	phosphoglycerate mutase 1 [Source:MGI Symbol;Acc:MGI:97552]
Mta3	1526.12767	1317.72106	0.21	0.033918231	0.980354196	1.469566806	Mta3	metastasis associated 3 [Source:MGI Symbol;Acc:MGI:2151172]
Smarcb1	1641.39006	1421.33136	0.21	0.027836852	0.980354196	1.55537988	Smarcb1	SWI/SNF related, matrix associated, actin dependent regulator of chromatin, subfamily b, member 1 [Source:MGI
Fam78a	2230.20549	1932.31429	0.21	0.038391786	0.980354196	1.415761684	Fam78a	family with sequence similarity 78, member A [Source:MGI Symbol;Acc:MGI:2443569]
Tial1	1740.03774	1520.85431	0.19	0.04939607	0.980354196	1.306307603	Tial1	Tia1 cytotoxic granule-associated RNA binding protein-like 1 [Source:MGI Symbol;Acc:MGI:107913]
Anapc2	1943.74376	1710.61731	0.18	0.033311148	0.980354196	1.4774104	Anapc2	anaphase promoting complex subunit 2 [Source:MGI Symbol;Acc:MGI:2139135]
Mybbp1a	4115.01408	3631.26269	0.18	0.04405971	0.980354196	1.355958366	Mybbp1a	MYB binding protein (P160) 1a [Source:MGI Symbol;Acc:MGI:106181]
**Decreased expression in Mg2800 versus Mg500**								
Gm35657	0	10.0689043	-5.79	0.001829515	0.980354196	2.737664025	Gm35657	predicted gene, 35657 [Source:MGI Symbol;Acc:MGI:5594816]
Gm25654	0	9.00621041	-5.63	0.000151966	0.879365235	3.818253568	Gm25654	predicted gene, 25654 [Source:MGI Symbol;Acc:MGI:5455431]
Slc35f3	0	6.45984423	-5.15	0.006932969	0.980354196	2.159080742	Slc35f3	solute carrier family 35, member F3 [Source:MGI Symbol;Acc:MGI:2444426]
Gm48732	0	5.97795234	-5.04	0.002399309	0.980354196	2.619913817	Gm48732	predicted gene, 48732 [Source:MGI Symbol;Acc:MGI:6098394]
Prss39	0	5.70796478	-4.97	0.049501872	0.980354196	1.305378377	Prss39	protease, serine 39 [Source:MGI Symbol;Acc:MGI:1270856]
Gm43962	0.12357885	7.11023157	-4.88	0.00418456	0.980354196	2.378350201	Gm43962	predicted gene, 43962 [Source:MGI Symbol;Acc:MGI:5690354]
D730045B01Rik	0.2471577	8.98912116	-4.80	6.41E-05	0.808222723	4.19314197	D730045B01Rik	RIKEN cDNA D730045B01 gene [Source:MGI Symbol;Acc:MGI:1925117]
AC158985.3	0.51799448	14.5461561	-4.77	0.00065185	0.980354196	3.18585233	AC158985.3	novel transcript
Gm47870	0	4.95451406	-4.77	0.013876531	0.980354196	1.85771909	Gm47870	predicted gene, 47870 [Source:MGI Symbol;Acc:MGI:6097090]
Gm26461	0	4.92336843	-4.76	0.00351731	0.980354196	2.453789353	Gm26461	predicted gene, 26461 [Source:MGI Symbol;Acc:MGI:5456238]
Gm28306	0	4.73863265	-4.70	0.014953823	0.980354196	1.825247764	Gm28306	predicted gene, 28306 [Source:MGI Symbol;Acc:MGI:5579012]
Gm10874	0.1791467	6.25304044	-4.69	0.000909286	0.980354196	3.041299496	Gm10874	predicted gene 10874 [Source:MGI Symbol;Acc:MGI:3704261]
Gm35147	0	4.68464441	-4.68	0.004853906	0.980354196	2.313908638	Gm35147	predicted gene, 35147 [Source:MGI Symbol;Acc:MGI:5594306]
9330111N05Rik	0	4.38175334	-4.59	0.001664016	0.980354196	2.778842502	9330111N05Rik	RIKEN cDNA 9330111N05 gene [Source:MGI Symbol;Acc:MGI:2443112]
Gm5432	0	4.34288642	-4.58	0.024250162	0.980354196	1.615285356	Gm5432	predicted gene 5432 [Source:MGI Symbol;Acc:MGI:3649057]
Gm38327	0.27823568	7.12444465	-4.46	0.001237616	0.980354196	2.907414085	Gm38327	predicted gene, 38327 [Source:MGI Symbol;Acc:MGI:5611555]
Lim2	0	3.98754675	-4.46	0.030374249	0.980354196	1.517494451	Lim2	lens intrinsic membrane protein 2 [Source:MGI Symbol;Acc:MGI:104698]
9430037G07Rik	0	3.93979309	-4.44	0.013645758	0.980354196	1.865002335	9430037G07Rik	RIKEN cDNA 9430037G07 gene [Source:MGI Symbol;Acc:MGI:2444544]
Gm12766	0.22589852	6.96476541	-4.43	0.000526076	0.980354196	3.278951511	Gm12766	predicted gene 12766 [Source:MGI Symbol;Acc:MGI:3652050]
Gm47321	0.27823568	6.90796671	-4.42	0.00644502	0.980354196	2.190775731	Gm47321	predicted gene, 47321 [Source:MGI Symbol;Acc:MGI:6096205]
Gm16573	0.11294926	5.15940633	-4.41	0.011406934	0.980354196	1.942831071	Gm16573	predicted gene 16573 [Source:MGI Symbol;Acc:MGI:4414993]
Mir343	0.33884778	8.69210025	-4.37	0.001835624	0.980354196	2.736216273	Mir343	microRNA 343 [Source:MGI Symbol;Acc:MGI:3718510]
A830035A12Rik	0.11294926	4.95557674	-4.36	0.01363015	0.980354196	1.865499365	A830035A12Rik	RIKEN cDNA A830035A12 gene [Source:MGI Symbol;Acc:MGI:3045382]
Gm8895	0.11294926	4.91284344	-4.34	0.016094219	0.980354196	1.793330093	Gm8895	predicted gene 8895 [Source:MGI Symbol;Acc:MGI:3643891]

Gm42901	0	3.68387972	-4.34	0.020721168	0.980354196	1.683585768	Gm42901	predicted gene 42901 [Source:MGI Symbol;Acc:MGI:5663038]
Gm29114	0	3.39250587	-4.22	0.047365683	0.980354196	1.324536196	Gm29114	predicted gene 29114 [Source:MGI Symbol;Acc:MGI:5579820]
Gm15849	0	3.39175504	-4.22	0.042482679	0.980354196	1.371788104	Gm15849	predicted gene 15849 [Source:MGI Symbol;Acc:MGI:3802034]
Gm45754	0.29209596	5.84943296	-4.18	0.004658304	0.980354196	2.331772173	Gm45754	predicted gene 45754 [Source:MGI Symbol;Acc:MGI:5804869]
Fhl5	0.2471577	5.80695657	-4.17	0.002528398	0.980354196	2.597154562	Fhl5	four and a half LIM domains 5 [Source:MGI Symbol;Acc:MGI:1913192]
Gm24524	0	3.27763677	-4.17	0.019903804	0.980354196	1.701063914	Gm24524	predicted gene, 24524 [Source:MGI Symbol;Acc:MGI:5454301]
Kctd4	0	3.26936937	-4.17	0.044576022	0.980354196	1.350898691	Kctd4	potassium channel tetramerisation domain containing 4 [Source:MGI Symbol;Acc:MGI:1914766]
C1ql4	0	3.2242046	-4.14	0.029824083	0.980354196	1.525432901	C1ql4	complement component 1, q subcomponent-like 4 [Source:MGI Symbol;Acc:MGI:3579909]
Gm47258	0.51799448	9.30723418	-4.13	0.008126071	0.980354196	2.090119387	Gm47258	predicted gene, 47258 [Source:MGI Symbol;Acc:MGI:6096089]
Gm15948	0.33884778	7.20147757	-4.11	0.039169745	0.980354196	1.407049256	Gm15948	predicted gene 15948 [Source:MGI Symbol;Acc:MGI:3801731]
Gldc	0.22589852	5.51331499	-4.10	0.020155653	0.980354196	1.695603127	Gldc	glycine decarboxylase [Source:MGI Symbol;Acc:MGI:1341155]
Tmcc3os	0.45179704	8.53940051	-4.07	0.00994162	0.980354196	2.002542841	Tmcc3os	transmembrane and coiled coil domains 3, opposite strand [Source:MGI Symbol;Acc:MGI:3801736]
A830009L08Rik	0.13911784	3.82186342	-3.98	0.038192114	0.980354196	1.418026302	A830009L08Rik	RIKEN cDNA A830009L08 gene [Source:MGI Symbol;Acc:MGI:2443762]
Gm12367	0.12357885	3.79328975	-3.97	0.038278823	0.980354196	1.417041424	Gm12367	predicted gene 12367 [Source:MGI Symbol;Acc:MGI:3649554]
Gm15470	0	2.84166859	-3.97	0.038296801	0.980354196	1.416837502	Gm15470	predicted gene 15470 [Source:MGI Symbol;Acc:MGI:3641891]
Gm7045	1.02773279	15.6677481	-3.96	0.000457401	0.980354196	3.33970289	Gm7045	predicted gene 7045 [Source:MGI Symbol;Acc:MGI:3779655]
AA672651	0	2.77184388	-3.93	0.037124575	0.980354196	1.430338509	AA672651	expressed sequence AA672651 [Source:MGI Symbol;Acc:MGI:2142438]
Gm23553	0.11294926	3.65746929	-3.92	0.042606718	0.980354196	1.370521918	Gm23553	predicted gene, 23553 [Source:MGI Symbol;Acc:MGI:5453330]
Otof	0.3582934	4.51074422	-3.81	0.032287518	0.980354196	1.490965339	Otof	otoferlin [Source:MGI Symbol;Acc:MGI:1891247]
Ccdc36	0.5647463	8.54993656	-3.77	0.002437212	0.980354196	2.613106692	Ccdc36	coiled-coil domain containing 36 [Source:MGI Symbol;Acc:MGI:3612242]
Fmr1nb	0.27823568	4.41004792	-3.77	0.046922965	0.980354196	1.328614553	Fmr1nb	Fmr1 neighbor [Source:MGI Symbol;Acc:MGI:2672032]
Gm49249	0.1791467	3.22640157	-3.74	0.045204257	0.980354196	1.344820665	Gm49249	predicted gene, 49249 [Source:MGI Symbol;Acc:MGI:6118719]
Gsdmc4	0.27823568	4.27631423	-3.73	0.041882434	0.980354196	1.377968087	Gsdmc4	gasdermin C4 [Source:MGI Symbol;Acc:MGI:1921798]
Gm45041	0.36010696	5.51500801	-3.69	0.026470035	0.980354196	1.577245484	Gm45041	predicted gene 45041 [Source:MGI Symbol;Acc:MGI:5753617]
Gm5608	0.13911784	3.05644128	-3.66	0.033288672	0.980354196	1.47770353	Gm5608	predicted gene 5608 [Source:MGI Symbol;Acc:MGI:3779504]
Agbl1	0.49741124	5.37910627	-3.63	0.015344023	0.980354196	1.814060759	Agbl1	ATP/GTP binding protein-like 1 [Source:MGI Symbol;Acc:MGI:3646469]
Gm37052	0.45179704	6.22252232	-3.61	0.019594381	0.980354196	1.707868452	Gm37052	predicted gene, 37052 [Source:MGI Symbol;Acc:MGI:5610280]
9430064I24Rik	0.811904	10.3915225	-3.61	0.022320375	0.980354196	1.651298513	9430064I24Rik	RIKEN cDNA 9430064I24 gene [Source:MGI Symbol;Acc:MGI:3704302]
Gm11934	0.30487875	3.89325092	-3.60	0.016417502	0.980354196	1.784692922	Gm11934	predicted gene 11934 [Source:MGI Symbol;Acc:MGI:3649212]
Pax6	0.22589852	3.88078263	-3.59	0.026654029	0.980354196	1.574237134	Pax6	paired box 6 [Source:MGI Symbol;Acc:MGI:97490]
Gm12925	0.36010696	5.05359243	-3.57	0.009143133	0.980354196	2.038904963	Gm12925	predicted gene 12925 [Source:MGI Symbol;Acc:MGI:3651536]
Nanos3	0.12357885	2.8591653	-3.56	0.03385083	0.980354196	1.470430678	Nanos3	nanos C2HC-type zinc finger 3 [Source:MGI Symbol;Acc:MGI:2675387]
Gm41071	0.53032155	6.32763622	-3.55	0.009783463	0.980354196	2.009507393	Gm41071	predicted gene, 41071 [Source:MGI Symbol;Acc:MGI:5623956]
Trim72	0.47305622	6.04586815	-3.52	0.007195731	0.980354196	2.14292508	Trim72	tripartite motif-containing 72 [Source:MGI Symbol;Acc:MGI:3612190]
Gm31683	0.34947737	4.68380322	-3.48	0.028016938	0.980354196	1.552579331	Gm31683	predicted gene, 31683 [Source:MGI Symbol;Acc:MGI:5590842]
Gm40645	0.45179704	5.59505903	-3.45	0.014151613	0.980354196	1.849194056	Gm40645	predicted gene, 40645 [Source:MGI Symbol;Acc:MGI:5623530]
4921529L05Rik	0.5098544	5.75273889	-3.39	0.007546967	0.980354196	2.122227549	4921529L05Rik	RIKEN cDNA 4921529L05 gene [Source:MGI Symbol;Acc:MGI:2443531]
Gm26407	0.70144756	7.22822744	-3.38	0.036243956	0.980354196	1.440764405	Gm26407	predicted gene, 26407 [Source:MGI Symbol;Acc:MGI:5456184]
8430419K02Rik	0.59091488	6.55293531	-3.37	0.015337388	0.980354196	1.814248596	8430419K02Rik	RIKEN cDNA 8430419K02 gene [Source:MGI Symbol;Acc:MGI:1921768]
Raet1d	1.20208618	12.3715439	-3.34	0.002515668	0.980354196	2.599346675	Raet1d	retinoic acid early transcript delta [Source:MGI Symbol;Acc:MGI:1861032]
Gm45607	0.69714118	6.84283366	-3.30	0.007355729	0.980354196	2.133374279	Gm45607	predicted gene 45607 [Source:MGI Symbol;Acc:MGI:5791443]
Mas1	0.57537589	5.98320516	-3.28	0.04358309	0.980354196	1.360681982	Mas1	MAS1 oncogene [Source:MGI Symbol;Acc:MGI:96918]
Gm15443	0.39118494	4.10379409	-3.26	0.017314994	0.980354196	1.761577655	Gm15443	predicted gene 15443 [Source:MGI Symbol;Acc:MGI:3642525]
Gm15512	0.73003272	7.46744146	-3.26	0.005091526	0.980354196	2.293152034	Gm15512	predicted gene 15512 [Source:MGI Symbol;Acc:MGI:3782960]
B930036N10Rik	0.81567578	7.51454593	-3.26	0.044607691	0.980354196	1.350590256	B930036N10Rik	RIKEN cDNA B930036N10 gene [Source:MGI Symbol;Acc:MGI:3702496]
Gm15342	1.26619386	11.9466839	-3.21	0.008818633	0.980354196	2.054598731	Gm15342	predicted gene 15342 [Source:MGI Symbol;Acc:MGI:3705188]
Slc22a12	0.85361157	7.52813181	-3.07	0.016279629	0.980354196	1.788355497	Slc22a12	solute carrier family 22 (organic anion/cation transporter), member 12 [Source:MGI Symbol;Acc:MGI:1195269]
Gm15547	1.15414673	10.2604889	-3.06	0.006756002	0.980354196	2.170310231	Gm15547	predicted gene 15547 [Source:MGI Symbol;Acc:MGI:3782996]
Gm37755	1.90513951	16.0673033	-3.05	0.01061788	0.980354196	1.973962187	Gm37755	predicted gene, 37755 [Source:MGI Symbol;Acc:MGI:5610983]
Gm47963	1.52428632	12.7695584	-3.03	0.012011914	0.980354196	1.920387786	Gm47963	predicted gene, 47963 [Source:MGI Symbol;Acc:MGI:6097241]
Gm36584	0.59663507	5.12713111	-3.00	0.024708501	0.980354196	1.607153601	Gm36584	predicted gene, 36584 [Source:MGI Symbol;Acc:MGI:5595743]
Trav7d-3	0.743893	6.12241522	-2.99	0.030540274	0.980354196	1.515127071	Trav7d-3	T cell receptor alpha variable 7D-3 [Source:MGI Symbol;Acc:MGI:3649861]
Snord65	0.7570121	6.1878096	-2.97	0.017039043	0.980354196	1.768554801	Snord65	small nucleolar RNA, C/D box 65 [Source:MGI Symbol;Acc:MGI:3819547]
Gm25821	0.82744299	6.74815951	-2.96	0.044005771	0.980354196	1.356490366	Gm25821	predicted gene, 25821 [Source:MGI Symbol;Acc:MGI:5455598]
Gm48714	1.16138137	9.12870182	-2.90	0.022331505	0.980354196	1.651082007	Gm48714	predicted gene, 48714 [Source:MGI Symbol;Acc:MGI:6098361]
Gm45437	1.49209238	11.3276121	-2.90	0.042802074	0.980354196	1.368535186	Gm45437	predicted gene 45437 [Source:MGI Symbol;Acc:MGI:5791273]
Cpa6	0.6103605	4.21660237	-2.87	0.044469915	0.980354196	1.351933701	Cpa6	carboxypeptidase A6 [Source:MGI Symbol;Acc:MGI:3045348]
Gm26811	1.74657605	13.0521835	-2.86	0.048228471	0.980354196	1.316696506	Gm26811	predicted gene, 26811 [Source:MGI Symbol;Acc:MGI:5477305]
Gm43961	1.80853191	12.9781733	-2.84	0.036080738	0.980354196	1.442724588	Gm43961	predicted gene, 43961 [Source:MGI Symbol;Acc:MGI:5690353]
Gm37818	1.53453781	11.1092803	-2.81	0.038644835	0.980354196	1.412908543	Gm37818	predicted gene, 37818 [Source:MGI Symbol;Acc:MGI:5611046]
Gm8655	0.96656083	7.06950473	-2.81	0.030115336	0.980354196	1.521212287	Gm8655	predicted gene 8655 [Source:MGI Symbol;Acc:MGI:3647209]
6720464F23Rik	1.27433063	9.27002117	-2.80	0.040982035	0.980354196	1.38740648	6720464F23Rik	RIKEN cDNA 6720464F23 gene [Source:MGI Symbol;Acc:MGI:2444677]
Mir7240	1.23416691	8.93441585	-2.79	0.009040986	0.980354196	2.043784203	Mir7240	microRNA 7240 [Source:MGI Symbol;Acc:MGI:5530869]
AC183097.2	1.46834037	10.6651183	-2.79	0.023070703	0.980354196	1.636939172	AC183097.2	tubby like protein 4 (Tulp4) pseudogene
Gm16350	1.23222483	8.3830415	-2.76	0.011332714	0.980354196	1.945666071	Gm16350	predicted gene 16350 [Source:MGI Symbol;Acc:MGI:3840133]
Gng4	1.59918327	11.2906426	-2.76	0.004721427	0.980354196	2.325926721	Gng4	guanine nucleotide binding protein (G protein), gamma 4 [Source:MGI Symbol;Acc:MGI:102703]
Rpl39l	0.53925366	3.6435753	-2.73	0.044262607	0.980354196	1.35396301	Rpl39l	ribosomal protein L39-like [Source:MGI Symbol;Acc:MGI:1915422]
Gm46218	2.29127084	15.2524599	-2.69	0.007197352	0.980354196	2.142827257	Gm46218	predicted gene, 46218 [Source:MGI Symbol;Acc:MGI:5825855]
Ighv2-4	10.4967077	67.5109322	-2.68	0.0111183	0.980354196	1.953961612	Ighv2-4	immunoglobulin heavy variable V2-4 [Source:MGI Symbol;Acc:MGI:3643263]
Gm43445	2.4734946	15.7403122	-2.65	0.010072467	0.980354196	1.996864147	Gm43445	predicted gene 43445 [Source:MGI Symbol;Acc:MGI:5663582]
Gm8979	2.61650524	15.2705193	-2.52	0.00538343	0.980354196	2.26894093	Gm8979	predicted gene 8979 [Source:MGI Symbol;Acc:MGI:3703149]
Ascl4	2.26080343	13.076947	-2.51	0.004829525	0.980354196	2.316095581	Ascl4	achaete-scute family bHLH transcription factor 4 [Source:MGI Symbol;Acc:MGI:1914591]
Abcc2	1.0095413	5.95982053	-2.51	0.034007686	0.980354196	1.468422918	Abcc2	ATP-binding cassette, sub-family C (CFTR/MRP), member 2 [Source:MGI Symbol;Acc:MGI:1352447]
Trav8n-2	1.18964303	6.56849484	-2.50	0.03417982	0.980354196	1.466230229	Trav8n-2	T cell receptor alpha variable 8n-2 [Source:MGI Symbol;Acc:MGI:3641705]
Rangrf	1.30440585	7.46574849	-2.49	0.025078833	0.980354196	1.600692677	Rangrf	RAN guanine nucleotide release factor [Source:MGI Symbol;Acc:MGI:1889073]
Gm16044	2.26673323	12.7237941	-2.48	0.023851291	0.980354196	1.622488109	Gm16044	predicted gene 16044 [Source:MGI Symbol;Acc:MGI:3801992]
Gm10392	1.95849225	10.6400553	-2.47	0.016569475	0.980354196	1.780691252	Gm10392	predicted gene 10392 [Source:MGI Symbol;Acc:MGI:3704377]
4930447K03Rik	1.62018088	8.6283792	-2.41	0.030299834	0.980354196	1.518559751	4930447K03Rik	RIKEN cDNA 4930447K03 gene [Source:MGI Symbol;Acc:MGI:1921231]
Gm47391	1.64676466	8.67868117	-2.33	0.032028487	0.980354196	1.494463577	Gm47391	predicted gene, 47391 [Source:MGI Symbol;Acc:MGI:6096314]
Atxn7l1os2	3.27171862	16.444616	-2.31	0.026357767	0.980354196	1.579091385	Atxn7l1os2	ataxin 7-like 1, opposite strand 2 [Source:MGI Symbol;Acc:MGI:1923086]

Gm49125	7.03269098	35.2934019	-2.30	0.005311905	0.980354196	2.274749701	Gm49125	predicted gene, 49125 [Source:MGI Symbol;Acc:MGI:6118530]
Gm20535	1.52488328	7.98717143	-2.30	0.016230883	0.980354196	1.789657853	Gm20535	predicted gene 20535 [Source:MGI Symbol;Acc:MGI:5142000]
Slitrk5	2.16859743	10.7476523	-2.26	0.032000218	0.980354196	1.494847063	Slitrk5	SLIT and NTRK-like family, member 5 [Source:MGI Symbol;Acc:MGI:2679448]
Gm45540	6.53793054	31.4095057	-2.26	0.024688606	0.980354196	1.607503431	Gm45540	predicted gene 45540 [Source:MGI Symbol;Acc:MGI:5791376]
Pcdhgb1	3.51650563	16.7216987	-2.21	0.013762669	0.980354196	1.861297335	Pcdhgb1	protocadherin gamma subfamily B, 1 [Source:MGI Symbol;Acc:MGI:1935169]
Gm17971	4.38592634	20.6427301	-2.20	0.003554635	0.980354196	2.449204987	Gm17971	predicted gene, 17971 [Source:MGI Symbol;Acc:MGI:5010156]
Gm29243	1.91812376	8.84318448	-2.20	0.036891405	0.980354196	1.433074804	Gm29243	predicted gene 29243 [Source:MGI Symbol;Acc:MGI:5579949]
Gm10432	2.70489504	11.7010013	-2.14	0.015836331	0.980354196	1.80034543	Gm10432	predicted gene 10432 [Source:MGI Symbol;Acc:MGI:3642011]
Dao	2.44452803	10.7982977	-2.11	0.04875229	0.980354196	1.31200498	Dao	D-amino acid oxidase [Source:MGI Symbol;Acc:MGI:94859]
Gm44986	6.00726657	25.7880272	-2.08	0.00452026	0.980354196	2.344836584	Gm44986	predicted gene 44986 [Source:MGI Symbol;Acc:MGI:5753562]
Tmprss9	1.45347733	6.42964677	-2.08	0.039501795	0.980354196	1.403383169	Tmprss9	transmembrane protease, serine 9 [Source:MGI Symbol;Acc:MGI:3612246]
Tmem202	2.62658925	10.9590436	-2.07	0.032243162	0.980354196	1.491562375	Tmem202	transmembrane protein 202 [Source:MGI Symbol;Acc:MGI:1921143]
Gm36931	2.69772505	11.3820048	-2.06	0.040159997	0.980354196	1.396206328	Gm36931	predicted gene, 36931 [Source:MGI Symbol;Acc:MGI:5610159]
Gm28800	4.71829314	19.4235205	-2.04	0.011546906	0.980354196	1.93753437	Gm28800	predicted gene 28800 [Source:MGI Symbol;Acc:MGI:5579506]
Gm44164	8.27080524	33.6777492	-2.03	0.015716663	0.980354196	1.803639659	Gm44164	predicted gene, 44164 [Source:MGI Symbol;Acc:MGI:5690556]
6430590A07Rik	5.93448056	24.2949548	-2.02	0.03777797	0.980354196	1.422761383	6430590A07Rik	RIKEN cDNA 6430590A07 gene [Source:MGI Symbol;Acc:MGI:3648839]
Gm20421	2.47298292	10.2410993	-2.01	0.042077724	0.980354196	1.375947759	Gm20421	predicted gene 20421 [Source:MGI Symbol;Acc:MGI:5141886]
Gm42735	3.98064767	15.9682993	-2.00	0.046700984	0.980354196	1.330673969	Gm42735	predicted gene 42735 [Source:MGI Symbol;Acc:MGI:5662872]
Ano5	7.07399687	28.5349198	-1.99	0.002693184	0.980354196	2.569733974	Ano5	anoctamin 5 [Source:MGI Symbol;Acc:MGI:3576659]
CT025601.1	3.37092408	13.3166672	-1.95	0.026068655	0.980354196	1.583881375	CT025601.1	transmembrane protein 167 (Tmem167) pseudogene
Gm34983	4.51504635	17.013619	-1.93	0.042086027	0.980354196	1.375862071	Gm34983	predicted gene, 34983 [Source:MGI Symbol;Acc:MGI:5594142]
Gm42664	13.6996043	52.382917	-1.93	0.032461276	0.980354196	1.488634413	Gm42664	predicted gene 42664 [Source:MGI Symbol;Acc:MGI:5662801]
B230334C09Rik	2.91511367	10.8792348	-1.92	0.016274941	0.980354196	1.788480577	B230334C09Rik	RIKEN cDNA B230334C09 gene [Source:MGI Symbol;Acc:MGI:2442224]
Gm6685	4.84630679	18.7151599	-1.91	0.008171234	0.980354196	2.087712352	Gm6685	predicted pseudogene 6685 [Source:MGI Symbol;Acc:MGI:3704119]
Shank2	3.46858724	12.8847065	-1.91	0.041034819	0.980354196	1.386847478	Shank2	SH3 and multiple ankyrin repeat domains 2 [Source:MGI Symbol;Acc:MGI:2671987]
Gm11536	2.64991956	9.89791736	-1.90	0.046792637	0.980354196	1.329822479	Gm11536	predicted gene 11536 [Source:MGI Symbol;Acc:MGI:3650116]
Gm13270	3.78577895	13.9686002	-1.88	0.038897572	0.980354196	1.410077507	Gm13270	predicted gene 13270 [Source:MGI Symbol;Acc:MGI:3651704]
Col19a1	5.43872399	19.5323319	-1.83	0.046226958	0.980354196	1.335104685	Col19a1	collagen, type XIX, alpha 1 [Source:MGI Symbol;Acc:MGI:1095415]
Gm38077	15.4101595	55.0491524	-1.83	0.007644021	0.980354196	2.116678129	Gm38077	predicted gene, 38077 [Source:MGI Symbol;Acc:MGI:5611305]
Sntg1	6.81221252	24.010487	-1.81	0.00575243	0.980354196	2.240148657	Sntg1	syntrophin, gamma 1 [Source:MGI Symbol;Acc:MGI:1918346]
Gm10180	2.98681561	10.344842	-1.80	0.037557406	0.980354196	1.425304411	Gm10180	predicted gene 10180 [Source:MGI Symbol;Acc:MGI:3701608]
C630043F03Rik	5.93783731	20.7495998	-1.79	0.046817915	0.980354196	1.329587931	C630043F03Rik	RIKEN cDNA C630043F03 gene [Source:MGI Symbol;Acc:MGI:1915535]
Gm16310	3.10386956	10.7814413	-1.76	0.040073749	0.980354196	1.397140026	Gm16310	predicted gene 16310 [Source:MGI Symbol;Acc:MGI:3826522]
Aldh1l2	10.7478111	36.903803	-1.76	0.003276355	0.980354196	2.484609048	Aldh1l2	aldehyde dehydrogenase 1 family, member L2 [Source:MGI Symbol;Acc:MGI:2444680]
Gm5970	3.53400284	11.9825512	-1.75	0.049344172	0.980354196	1.306764134	Gm5970	predicted gene 5970 [Source:MGI Symbol;Acc:MGI:3646761]
Gm49395	2.64372796	9.06750895	-1.75	0.01367992	0.980354196	1.863916442	Gm49395	predicted gene, 49395 [Source:MGI Symbol;Acc:MGI:6121627]
Gm5469	2.09177683	7.01263486	-1.74	0.031958064	0.980354196	1.495419538	Gm5469	predicted gene 5469 [Source:MGI Symbol;Acc:MGI:3645122]
Lama1	5.76389772	19.2774497	-1.72	0.023065279	0.980354196	1.637041288	Lama1	laminin, alpha 1 [Source:MGI Symbol;Acc:MGI:99892]
Gm44860	9.22537146	29.0150703	-1.65	0.041250034	0.980354196	1.384575689	Gm44860	predicted gene 44860 [Source:MGI Symbol;Acc:MGI:5753436]
Fdx1l	3.63264848	11.5160141	-1.65	0.037193762	0.980354196	1.429529892	Fdx1l	ferredoxin 1-like [Source:MGI Symbol;Acc:MGI:1915415]
Prss29	4.33864776	13.574803	-1.64	0.021126162	0.980354196	1.675179394	Prss29	protease, serine 29 [Source:MGI Symbol;Acc:MGI:2149952]
Clca2	8.09034708	25.1695457	-1.62	0.002663166	0.980354196	2.574601762	Clca2	chloride channel accessory 2 [Source:MGI Symbol;Acc:MGI:2139758]
Tenm2	7.93941381	23.7486277	-1.61	0.0022773	0.980354196	2.642579754	Tenm2	teneurin transmembrane protein 2 [Source:MGI Symbol;Acc:MGI:1345184]
Gm18537	3.63870912	11.0254794	-1.58	0.042612914	0.980354196	1.370458766	Gm18537	predicted gene, 18537 [Source:MGI Symbol;Acc:MGI:5010722]
Nlrp1c-ps	14.879555	44.5136731	-1.57	0.042747291	0.980354196	1.369091402	Nlrp1c-ps	NLR family, pyrin domain containing 1C, pseudogene [Source:MGI Symbol;Acc:MGI:3582962]
Gm47467	4.21680919	12.5561268	-1.56	0.038587331	0.980354196	1.41355526	Gm47467	predicted gene, 47467 [Source:MGI Symbol;Acc:MGI:6096433]
Gm16070	4.45650939	12.9883794	-1.56	0.037012944	0.980354196	1.43164637	Gm16070	predicted gene 16070 [Source:MGI Symbol;Acc:MGI:3801761]
Gm44141	6.54122291	19.4371384	-1.54	0.027214985	0.980354196	1.565191901	Gm44141	predicted gene, 44141 [Source:MGI Symbol;Acc:MGI:5690533]
Tmigd3	7.19625144	20.7195837	-1.53	0.031935842	0.980354196	1.495721629	Tmigd3	transmembrane and immunoglobulin domain containing 3 [Source:MGI Symbol;Acc:MGI:5604098]
Gm15932	6.59542848	18.9332006	-1.51	0.008853905	0.980354196	2.052865142	Gm15932	predicted gene 15932 [Source:MGI Symbol;Acc:MGI:3802106]
Ccl11	5.99713884	17.2926566	-1.50	0.031832644	0.980354196	1.497127288	Ccl11	chemokine (C-C motif) ligand 11 [Source:MGI Symbol;Acc:MGI:103576]
Paqr3	13.7321728	39.0068773	-1.50	0.009882087	0.980354196	2.005151327	Paqr3	progestin and adipoQ receptor family member III [Source:MGI Symbol;Acc:MGI:2679683]
Mir1898	7.37118986	21.1457451	-1.49	0.037756953	0.980354196	1.423003061	Mir1898	microRNA 1898 [Source:MGI Symbol;Acc:MGI:3811421]
Obscn	48.090775	135.234643	-1.49	0.002572593	0.980354196	2.589628916	Obscn	obscurin, cytoskeletal calmodulin and titin-interacting RhoGEF [Source:MGI Symbol;Acc:MGI:2681862]
Ccdc162	7.77717358	21.7107579	-1.47	0.045808849	0.980354196	1.33905062	Ccdc162	coiled-coil domain containing 162 [Source:MGI Symbol;Acc:MGI:1923223]
Gm16024	7.72435116	21.1309458	-1.45	0.035508667	0.980354196	1.449665631	Gm16024	predicted gene 16024 [Source:MGI Symbol;Acc:MGI:3802145]
Gm44321	22.107258	59.4640998	-1.42	0.047219922	0.980354196	1.325874735	Gm44321	predicted gene, 44321 [Source:MGI Symbol;Acc:MGI:5690713]
Mir8091	8.0104789	21.5675725	-1.42	0.032096073	0.980354196	1.493548101	Mir8091	microRNA 8091 [Source:MGI Symbol;Acc:MGI:5530957]
Calml4	13.8663839	37.2760138	-1.41	0.008712385	0.980354196	2.059862941	Calml4	calmodulin-like 4 [Source:MGI Symbol;Acc:MGI:1922850]
Gm44423	48.7326376	129.951027	-1.41	0.018847662	0.980354196	1.724742515	Gm44423	predicted gene, 44423 [Source:MGI Symbol;Acc:MGI:5690815]
Tmtc2	23.4816153	62.2413559	-1.40	0.00401828	0.980354196	2.395959804	Tmtc2	transmembrane and tetratricopeptide repeat containing 2 [Source:MGI Symbol;Acc:MGI:1914057]
Ighv3-4	13.2544942	34.7104952	-1.39	0.020024459	0.980354196	1.698439208	Ighv3-4	immunoglobulin heavy variable V3-4 [Source:MGI Symbol;Acc:MGI:3644034]
Sycp2	9.64797914	25.0863626	-1.38	0.025631927	0.980354196	1.591218742	Sycp2	synaptonemal complex protein 2 [Source:MGI Symbol;Acc:MGI:1933281]
Gapdh	142.783878	369.876141	-1.37	0.004168481	0.980354196	2.380022174	Gapdh	glyceraldehyde-3-phosphate dehydrogenase [Source:MGI Symbol;Acc:MGI:95640]
4930439D14Rik	32.1381673	83.2312987	-1.37	0.015040784	0.980354196	1.822729526	4930439D14Rik	RIKEN cDNA 4930439D14 gene [Source:MGI Symbol;Acc:MGI:1925417]
Lrrc4	13.5186682	35.2448974	-1.37	0.040394051	0.980354196	1.393682591	Lrrc4	leucine rich repeat containing 4 [Source:MGI Symbol;Acc:MGI:2182081]
Gm45235	14.7353985	37.7398634	-1.36	0.021550272	0.980354196	1.666547244	Gm45235	predicted gene 45235 [Source:MGI Symbol;Acc:MGI:5753811]
Asgr2	6.11698657	15.628689	-1.36	0.012435831	0.980354196	1.905325189	Asgr2	asialoglycoprotein receptor 2 [Source:MGI Symbol;Acc:MGI:88082]
Gm9967	11.8618376	30.4317243	-1.34	0.016697253	0.980354196	1.777354972	Gm9967	predicted gene 9967 [Source:MGI Symbol;Acc:MGI:3704300]
Acvr2b	14.3852708	36.4047482	-1.32	0.027369445	0.980354196	1.562734009	Acvr2b	activin receptor IIB [Source:MGI Symbol;Acc:MGI:87912]
Nnat	35.7368558	89.189458	-1.31	0.012878703	0.980354196	1.890127872	Nnat	neuronatin [Source:MGI Symbol;Acc:MGI:104716]
Casq2	14.4238632	36.0698464	-1.30	0.013968207	0.980354196	1.854859338	Casq2	calsequestrin 2 [Source:MGI Symbol;Acc:MGI:1309469]
Sned1	318.576648	784.410989	-1.30	0.023985337	0.980354196	1.620054175	Sned1	sushi, nidogen and EGF-like domains 1 [Source:MGI Symbol;Acc:MGI:3045960]
Insrr	14.1877877	35.2921619	-1.30	0.011682278	0.980354196	1.932472463	Insrr	insulin receptor-related receptor [Source:MGI Symbol;Acc:MGI:1346037]
Neto1	9.60586909	23.8380308	-1.30	0.022938178	0.980354196	1.639441081	Neto1	neuropilin (NRP) and tolloid (TLL)-like 1 [Source:MGI Symbol;Acc:MGI:2180216]
Cyp2t4	10.0139304	25.0084748	-1.29	0.00797366	0.980354196	2.098342287	Cyp2t4	cytochrome P450, family 2, subfamily t, polypeptide 4 [Source:MGI Symbol;Acc:MGI:2686296]
Vmn2r84	47.1098237	115.478755	-1.29	0.015609707	0.980354196	1.806605249	Vmn2r84	vomeronasal 2, receptor 84 [Source:MGI Symbol;Acc:MGI:3643367]
Il20rb	17.8950182	43.9084061	-1.28	0.01339581	0.980354196	1.873031021	Il20rb	interleukin 20 receptor beta [Source:MGI Symbol;Acc:MGI:2143266]
Sec14l5	13.3530389	32.3475562	-1.28	0.034854471	0.980354196	1.457741504	Sec14l5	SEC14-like lipid binding 5 [Source:MGI Symbol;Acc:MGI:3616084]
4931431B13Rik	44.2191724	108.119309	-1.28	0.001803075	0.980354196	2.743986208	4931431B13Rik	RIKEN cDNA 4931431B13 gene [Source:MGI Symbol;Acc:MGI:1918223]

Gm28187	17.0403272	41.3978024	-1.27	0.033554958	0.980354196	1.4742433	Gm28187	predicted gene 28187 [Source:MGI Symbol;Acc:MGI:5578893]
Ighj3	40.0774736	97.245867	-1.27	0.026148725	0.980354196	1.582549482	Ighj3	immunoglobulin heavy joining 3 [Source:MGI Symbol;Acc:MGI:4439767]
Gm26581	31.4441216	76.2181603	-1.27	0.026646563	0.980354196	1.5743588	Gm26581	predicted gene, 26581 [Source:MGI Symbol;Acc:MGI:5477075]
CT025659.2	6.18276305	14.8805234	-1.26	0.032320883	0.980354196	1.490516783	CT025659.2	novel transcript
Lipn	17.2258621	41.632567	-1.26	0.023672716	0.980354196	1.625751912	Lipn	lipase, family member N [Source:MGI Symbol;Acc:MGI:1917416]
Gm38394	47.6236113	113.902905	-1.26	0.021767565	0.980354196	1.66219015	Gm38394	predicted gene, 38394 [Source:MGI Symbol;Acc:MGI:5614057]
BC005561	71.420731	170.66998	-1.25	0.002127308	0.980354196	2.672169627	BC005561	cDNA sequence BC005561 [Source:MGI Symbol;Acc:MGI:3040669]
Gm16015	44.2822253	105.249276	-1.24	0.007504905	0.980354196	2.124654801	Gm16015	predicted gene 16015 [Source:MGI Symbol;Acc:MGI:3801811]
Gm44850	23.8293998	56.5660748	-1.24	0.045881933	0.980354196	1.338358294	Gm44850	predicted gene 44850 [Source:MGI Symbol;Acc:MGI:5753426]
Ighv1-61	96.334031	227.501545	-1.24	0.006806645	0.980354196	2.167066899	Ighv1-61	immunoglobulin heavy variable 1-61 [Source:MGI Symbol;Acc:MGI:4439824]
Gm6627	16.1147506	37.9239271	-1.23	0.006626824	0.980354196	2.178694564	Gm6627	predicted gene 6627 [Source:MGI Symbol;Acc:MGI:3644486]
Kcnh1	35.6286658	83.7591105	-1.23	0.01133163	0.980354196	1.945707614	Kcnh1	potassium voltage-gated channel, subfamily H (eag-related), member 1 [Source:MGI Symbol;Acc:MGI:1341721]
Sv2b	26.4923564	61.8371614	-1.21	0.014246154	0.980354196	1.846302365	Sv2b	synaptic vesicle glycoprotein 2 b [Source:MGI Symbol;Acc:MGI:1927338]
Ppfia2	20.3898736	47.1878458	-1.21	0.008500115	0.980354196	2.070575199	Ppfia2	protein tyrosine phosphatase, receptor type, f polypeptide (PTPRF), interacting protein (liprin), alpha 2 [Source:M
Gm45884	81.4160398	187.162828	-1.20	0.029473365	0.980354196	1.530570278	Gm45884	predicted gene 45884 [Source:MGI Symbol;Acc:MGI:5804999]
Gm45220	24.4830305	56.0796814	-1.19	0.025263396	0.980354196	1.59750827	Gm45220	predicted gene 45220 [Source:MGI Symbol;Acc:MGI:5753796]
Slc2a13	25.5040807	58.1319691	-1.18	0.002790563	0.980354196	2.554308168	Slc2a13	solute carrier family 2 (facilitated glucose transporter), member 13 [Source:MGI Symbol;Acc:MGI:2146030]
Zfp871	383.077576	867.067264	-1.18	0.034210632	0.980354196	1.465838903	Zfp871	zinc finger protein 871 [Source:MGI Symbol;Acc:MGI:1921793]
Vsig2	19.8745122	45.1018468	-1.18	0.043918659	0.980354196	1.357350929	Vsig2	V-set and immunoglobulin domain containing 2 [Source:MGI Symbol;Acc:MGI:1928009]
Angptl7	31.2856778	69.3828375	-1.14	0.044346767	0.980354196	1.353138036	Angptl7	angiopoietin-like 7 [Source:MGI Symbol;Acc:MGI:3605801]
Atp7a	124.764617	275.686907	-1.14	0.024111983	0.980354196	1.617767071	Atp7a	ATPase, Cu++ transporting, alpha polypeptide [Source:MGI Symbol;Acc:MGI:99400]
Gm21762	22.1310774	48.2656969	-1.13	0.002378199	0.980354196	2.623751808	Gm21762	predicted gene, 21762 [Source:MGI Symbol;Acc:MGI:5433926]
Klhl29	115.875365	251.031616	-1.11	0.011983503	0.980354196	1.921416211	Klhl29	kelch-like 29 [Source:MGI Symbol;Acc:MGI:2683857]
Wdr95	10.8961355	23.6778234	-1.11	0.026139292	0.980354196	1.58270618	Wdr95	WD40 repeat domain 95 [Source:MGI Symbol;Acc:MGI:1923042]
Kcna3	121.896972	263.10013	-1.11	0.026290347	0.980354196	1.580203682	Kcna3	potassium voltage-gated channel, shaker-related subfamily, member 3 [Source:MGI Symbol;Acc:MGI:96660]
Gm20633	9.08145666	19.654159	-1.11	0.030293652	0.980354196	1.518648368	Gm20633	predicted gene 20633 [Source:MGI Symbol;Acc:MGI:5313080]
Glt8d2	14.9827448	32.2237831	-1.10	0.029773865	0.980354196	1.526164785	Glt8d2	glycosyltransferase 8 domain containing 2 [Source:MGI Symbol;Acc:MGI:1922032]
Fam184a	8.02212861	17.3094847	-1.10	0.04873699	0.980354196	1.312141296	Fam184a	family with sequence similarity 184, member A [Source:MGI Symbol;Acc:MGI:1923156]
Gprin3	67.1823906	143.884869	-1.10	0.021424578	0.980354196	1.669087724	Gprin3	GPRIN family member 3 [Source:MGI Symbol;Acc:MGI:1924785]
Zfp69	50.0473502	107.240593	-1.09	0.021348716	0.980354196	1.67062824	Zfp69	zinc finger protein 69 [Source:MGI Symbol;Acc:MGI:107794]
Ighv1-12	108.108742	227.03751	-1.07	0.006134929	0.980354196	2.212190459	Ighv1-12	immunoglobulin heavy variable V1-12 [Source:MGI Symbol;Acc:MGI:3646284]
9530082P21Rik	28.5577665	59.7866979	-1.06	0.003392134	0.980354196	2.469527	9530082P21Rik	RIKEN cDNA 9530082P21 gene [Source:MGI Symbol;Acc:MGI:3603340]
Prex2	171.115702	356.07616	-1.06	0.003414477	0.980354196	2.466675808	Prex2	phosphatidylinositol-3,4,5-trisphosphate-dependent Rac exchange factor 2 [Source:MGI Symbol;Acc:MGI:192338
Gm38190	49.4961763	102.367815	-1.04	0.022760885	0.980354196	1.642810855	Gm38190	predicted gene, 38190 [Source:MGI Symbol;Acc:MGI:5611418]
Gm15674	157.620105	323.563096	-1.04	0.021725468	0.980354196	1.663030859	Gm15674	predicted gene 15674 [Source:MGI Symbol;Acc:MGI:3783116]
Heatr9	31.7404268	65.2547239	-1.03	0.003927026	0.980354196	2.405936223	Heatr9	HEAT repeat containing 9 [Source:MGI Symbol;Acc:MGI:3650286]
Arsi	30.3198538	62.0893697	-1.03	0.031823391	0.980354196	1.497253545	Arsi	arylsulfatase i [Source:MGI Symbol;Acc:MGI:2670959]
Ryr2	28.8806008	59.332351	-1.03	0.03650769	0.980354196	1.437615646	Ryr2	ryanodine receptor 2, cardiac [Source:MGI Symbol;Acc:MGI:99685]
Fer1l6	13.0087194	26.2592346	-1.02	0.01288836	0.980354196	1.889802342	Fer1l6	fer-1-like 6 (C. elegans) [Source:MGI Symbol;Acc:MGI:3645398]
Stox2	51.8627836	105.318871	-1.02	0.019850541	0.980354196	1.702227653	Stox2	storkhead box 2 [Source:MGI Symbol;Acc:MGI:1918319]
Dhh	13.0856872	26.652112	-1.01	0.008428826	0.980354196	2.074232911	Dhh	desert hedgehog [Source:MGI Symbol;Acc:MGI:94891]
Igkv1-135	1181.49312	2382.35357	-1.01	0.005169937	0.980354196	2.286514749	Igkv1-135	immunoglobulin kappa variable 1-135 [Source:MGI Symbol;Acc:MGI:3819952]
Cdh4	61.5541162	123.870466	-1.01	0.003358499	0.980354196	2.473854777	Cdh4	cadherin 4 [Source:MGI Symbol;Acc:MGI:99218]
Lcor	144.444812	290.202965	-1.01	0.028112916	0.980354196	1.551094105	Lcor	ligand dependent nuclear receptor corepressor [Source:MGI Symbol;Acc:MGI:2443930]
Gdf6	289.823952	570.011037	-0.97	0.001009364	0.980354196	2.995952189	Gdf6	growth differentiation factor 6 [Source:MGI Symbol;Acc:MGI:95689]
Igkv12-41	88.503538	173.710164	-0.97	0.009657477	0.980354196	2.015136317	Igkv12-41	immunoglobulin kappa chain variable 12-41 [Source:MGI Symbol;Acc:MGI:4439772]
4732440D04Rik	71.026789	139.901264	-0.97	0.000799365	0.980354196	3.097254871	4732440D04Rik	RIKEN cDNA 4732440D04 gene [Source:MGI Symbol;Acc:MGI:3604103]
Nkpd1	35.967837	70.4697251	-0.96	0.015166023	0.980354196	1.81912829	Nkpd1	NTPase, KAP family P-loop domain containing 1 [Source:MGI Symbol;Acc:MGI:1916797]
Mest	29.2248275	57.0405744	-0.96	0.048232695	0.980354196	1.316658471	Mest	mesoderm specific transcript [Source:MGI Symbol;Acc:MGI:96968]
Ptar1	48.2146219	93.3937836	-0.96	0.012501727	0.980354196	1.903029989	Ptar1	protein prenyltransferase alpha subunit repeat containing 1 [Source:MGI Symbol;Acc:MGI:1921875]
Shisa6	39.9439041	77.4549676	-0.95	0.000838382	0.980354196	3.076558055	Shisa6	shisa family member 6 [Source:MGI Symbol;Acc:MGI:2685725]
Gm26642	24.3606481	47.4174137	-0.95	0.022501264	0.980354196	1.647793085	Gm26642	predicted gene, 26642 [Source:MGI Symbol;Acc:MGI:5477136]
Npff	20.0530113	38.9718878	-0.95	0.01427253	0.980354196	1.845499035	Npff	neuropeptide FF-amide peptide precursor [Source:MGI Symbol;Acc:MGI:1891708]
Cnr1	44.4462227	85.8005131	-0.95	0.004918672	0.980354196	2.308152137	Cnr1	cannabinoid receptor 1 (brain) [Source:MGI Symbol;Acc:MGI:104615]
Zfp971	81.6908908	156.049608	-0.93	0.028296841	0.980354196	1.548262045	Zfp971	zinc finger protein 971 [Source:MGI Symbol;Acc:MGI:1261426]
Gm45110	36.0437504	68.7658522	-0.93	0.043700793	0.980354196	1.359510682	Gm45110	predicted gene 45110 [Source:MGI Symbol;Acc:MGI:5753686]
Smpdl3b	21.1441101	40.5188487	-0.93	0.010849212	0.980354196	1.964601804	Smpdl3b	sphingomyelin phosphodiesterase, acid-like 3B [Source:MGI Symbol;Acc:MGI:1916022]
Sema3d	313.832601	596.151509	-0.93	0.003755623	0.980354196	2.42531801	Sema3d	sema domain, immunoglobulin domain (Ig), short basic domain, secreted, (semaphorin) 3D [Source:MGI Symbol;A
Frem1	83.7173443	159.077967	-0.92	0.02605415	0.980354196	1.584123091	Frem1	Fras1 related extracellular matrix protein 1 [Source:MGI Symbol;Acc:MGI:2670972]
Soga1	247.283272	466.5285	-0.91	0.003629746	0.980354196	2.440123765	Soga1	suppressor of glucose, autophagy associated 1 [Source:MGI Symbol;Acc:MGI:2444575]
Kif5a	32.9343627	62.409002	-0.91	0.011854532	0.980354196	1.926115587	Kif5a	kinesin family member 5A [Source:MGI Symbol;Acc:MGI:109564]
Layn	34.09724	63.83306	-0.91	0.042025405	0.980354196	1.376488093	Layn	layilin [Source:MGI Symbol;Acc:MGI:2685357]
Gm43328	62.9221939	117.788618	-0.90	0.035921855	0.980354196	1.444641245	Gm43328	predicted gene 43328 [Source:MGI Symbol;Acc:MGI:5663465]
Ighv5-9-1	115.655809	215.480927	-0.90	0.000111737	0.808222723	3.951802993	Ighv5-9-1	immunoglobulin heavy variable 5-9-1 [Source:MGI Symbol;Acc:MGI:4439810]
Gpr161	29.4712223	55.1821212	-0.89	0.019412515	0.980354196	1.711918196	Gpr161	G protein-coupled receptor 161 [Source:MGI Symbol;Acc:MGI:2685054]
Igkv6-20	97.6251316	180.527164	-0.89	0.001299253	0.980354196	2.886306272	Igkv6-20	immunoglobulin kappa variable 6-20 [Source:MGI Symbol;Acc:MGI:1330836]
Klri1	100.76644	186.100821	-0.88	0.003503691	0.980354196	2.455474202	Klri1	killer cell lectin-like receptor family I member 1 [Source:MGI Symbol;Acc:MGI:3530275]
Adgrl3	86.6284005	158.827736	-0.87	0.021661914	0.980354196	1.664303173	Adgrl3	adhesion G protein-coupled receptor L3 [Source:MGI Symbol;Acc:MGI:2441950]
Gm19461	32.5850863	59.9347642	-0.87	0.030449135	0.980354196	1.51642504	Gm19461	predicted gene, 19461 [Source:MGI Symbol;Acc:MGI:5011646]
Cxadr	56.688441	103.713665	-0.87	0.003994043	0.980354196	2.398587264	Cxadr	coxsackie virus and adenovirus receptor [Source:MGI Symbol;Acc:MGI:1201679]
Stard9	268.166254	488.252104	-0.86	0.021471455	0.980354196	1.668138525	Stard9	START domain containing 9 [Source:MGI Symbol;Acc:MGI:3045258]
Ighv5-4	549.32663	341.803229	-0.86	0.02129133	0.980354196	1.671797209	Ighv5-4	immunoglobulin heavy variable 5-4 [Source:MGI Symbol;Acc:MGI:4439895]
Rbfox1	18.2719285	33.3868752	-0.86	0.017612603	0.980354196	1.754176454	Rbfox1	RNA binding protein, fox-1 homolog (C. elegans) 1 [Source:MGI Symbol;Acc:MGI:1926224]
Cemip	184.021016	334.836438	-0.86	0.00135478	0.980354196	2.868131223	Cemip	cell migration inducing protein, hyaluronan binding [Source:MGI Symbol;Acc:MGI:2443629]
Arhgap32	159.190772	288.640127	-0.86	0.02054038	0.980354196	1.687391526	Arhgap32	Rho GTPase activating protein 32 [Source:MGI Symbol;Acc:MGI:2450166]
Gm12276	27.705997	50.2071683	-0.85	0.032247061	0.980354196	1.491509861	Gm12276	predicted gene 12276 [Source:MGI Symbol;Acc:MGI:3649700]
Pdcd1lg2	65.9588423	118.725078	-0.85	0.002507833	0.980354196	2.600701387	Pdcd1lg2	programmed cell death 1 ligand 2 [Source:MGI Symbol;Acc:MGI:1930125]
Dgki	39.8659863	71.3896376	-0.84	0.046360972	0.980354196	1.333847467	Dgki	diacylglycerol kinase, iota [Source:MGI Symbol;Acc:MGI:2443430]
Zfp423	75.0524579	134.968914	-0.84	0.031427579	0.980354196	1.502689073	Zfp423	zinc finger protein 423 [Source:MGI Symbol;Acc:MGI:1891217]
Chic1	57.5824118	103.344216	-0.84	0.016934181	0.980354196	1.771235803	Chic1	cysteine-rich hydrophobic domain 1 [Source:MGI Symbol;Acc:MGI:1344694]

Lama2	158.551067	281.541524	-0.83	0.005444436	0.980354196	2.264047103	Lama2	laminin, alpha 2 [Source:MGI Symbol;Acc:MGI:99912]
Gm16185	60.4824097	107.38776	-0.83	0.037868382	0.980354196	1.421723251	Gm16185	predicted gene 16185 [Source:MGI Symbol;Acc:MGI:3802169]
Zfp354c	80.1673488	141.448018	-0.81	0.033316483	0.980354196	1.47734085	Zfp354c	zinc finger protein 354C [Source:MGI Symbol;Acc:MGI:1353621]
Epha3	36.4311148	64.2364685	-0.81	0.035761531	0.980354196	1.446583897	Epha3	Eph receptor A3 [Source:MGI Symbol;Acc:MGI:99612]
Rnf113a1	50.792982	88.9094881	-0.81	0.027801002	0.980354196	1.555939551	Rnf113a1	ring finger protein 113A1 [Source:MGI Symbol;Acc:MGI:1917192]
Tmem200a	28.1874872	49.2839138	-0.80	0.021927304	0.980354196	1.659014762	Tmem200a	transmembrane protein 200A [Source:MGI Symbol;Acc:MGI:1924470]
Ccm2l	101.821533	178.290402	-0.80	0.00467453	0.980354196	2.330262049	Ccm2l	cerebral cavernous malformation 2-like [Source:MGI Symbol;Acc:MGI:2385159]
Pcdh18	255.238037	444.408518	-0.80	0.012075346	0.980354196	1.918100416	Pcdh18	protocadherin 18 [Source:MGI Symbol;Acc:MGI:1920423]
Efr3b	101.606624	176.345861	-0.80	0.048563798	0.980354196	1.313687356	Efr3b	EFR3 homolog B [Source:MGI Symbol;Acc:MGI:2444851]
Ptprg	170.224818	295.94056	-0.80	0.032283784	0.980354196	1.491015567	Ptprg	protein tyrosine phosphatase, receptor type, G [Source:MGI Symbol;Acc:MGI:97814]
Hmcn1	407.189823	703.368144	-0.79	0.037089911	0.980354196	1.430744209	Hmcn1	hemicentin 1 [Source:MGI Symbol;Acc:MGI:2685047]
Zfp13	23.1558805	39.995822	-0.79	0.028014479	0.980354196	1.55261745	Zfp13	zinc finger protein 13 [Source:MGI Symbol;Acc:MGI:99159]
Gm6483	21.3839632	37.2957899	-0.79	0.043077513	0.980354196	1.365749378	Gm6483	predicted gene 6483 [Source:MGI Symbol;Acc:MGI:3644574]
Skint3	67.8624726	117.313836	-0.78	0.01854634	0.980354196	1.731741783	Skint3	selection and upkeep of intraepithelial T cells 3 [Source:MGI Symbol;Acc:MGI:3045331]
Igkv6-17	498.18679	857.771657	-0.78	0.011997022	0.980354196	1.920926545	Igkv6-17	immunoglobulin kappa variable 6-17 [Source:MGI Symbol;Acc:MGI:1330833]
Slc35d3	38.8665932	67.0368155	-0.78	0.045412151	0.980354196	1.342827927	Slc35d3	solute carrier family 35, member D3 [Source:MGI Symbol;Acc:MGI:1923407]
Sdk2	186.294452	320.331033	-0.78	0.027664442	0.980354196	1.558078085	Sdk2	sidekick cell adhesion molecule 2 [Source:MGI Symbol;Acc:MGI:2443847]
Dntt	37.3977185	64.2706485	-0.78	0.010747235	0.980354196	1.968703255	Dntt	deoxynucleotidyltransferase, terminal [Source:MGI Symbol;Acc:MGI:98659]
Ppp1r3f	37.2130386	63.7832666	-0.78	0.035291982	0.980354196	1.452323951	Ppp1r3f	protein phosphatase 1, regulatory subunit 3F [Source:MGI Symbol;Acc:MGI:1859617]
Gucy1a1	510.120963	874.004331	-0.78	0.013826009	0.980354196	1.859303165	Gucy1a1	guanylate cyclase 1, soluble, alpha 1 [Source:MGI Symbol;Acc:MGI:1926562]
Card10	132.028342	226.498812	-0.78	0.038713058	0.980354196	1.412142522	Card10	caspase recruitment domain family, member 10 [Source:MGI Symbol;Acc:MGI:2146012]
Ophn1	55.1481391	94.2215893	-0.77	0.034115702	0.980354196	1.467045688	Ophn1	oligophrenin 1 [Source:MGI Symbol;Acc:MGI:2151070]
Terb1	50.9918931	87.6730726	-0.77	0.00779919	0.980354196	2.107950499	Terb1	telomere repeat binding bouquet formation protein 1 [Source:MGI Symbol;Acc:MGI:2443187]
Arhgap20	84.7401839	144.238205	-0.76	0.038753137	0.980354196	1.411693136	Arhgap20	Rho GTPase activating protein 20 [Source:MGI Symbol;Acc:MGI:2445175]
Ighv7-1	243.905066	413.036133	-0.76	0.021152331	0.980354196	1.674641766	Ighv7-1	immunoglobulin heavy variable 7-1 [Source:MGI Symbol;Acc:MGI:4439622]
Ptprb	1111.09138	1881.29169	-0.76	0.015875935	0.980354196	1.799260688	Ptprb	protein tyrosine phosphatase, receptor type, B [Source:MGI Symbol;Acc:MGI:97809]
AC130815.2	53.1582096	90.3084998	-0.76	0.030010106	0.980354196	1.522732471	AC130815.2	novel transcript
Cadps2	47.3840698	80.1203248	-0.75	0.017284378	0.980354196	1.762346244	Cadps2	Ca2+-dependent activator protein for secretion 2 [Source:MGI Symbol;Acc:MGI:2443963]
Hykk	30.8772994	52.3913542	-0.75	0.035548873	0.980354196	1.449174163	Hykk	hydroxylysine kinase 1 [Source:MGI Symbol;Acc:MGI:2443139]
Tnf	71.9322675	121.738523	-0.75	0.040308039	0.980354196	1.39460833	Tnf	tumor necrosis factor [Source:MGI Symbol;Acc:MGI:104798]
Gm5431	75.0512142	126.53033	-0.75	0.005845344	0.980354196	2.233189925	Gm5431	predicted gene 5431 [Source:MGI Symbol;Acc:MGI:3645205]
Nipal1	30.674207	51.3662966	-0.75	0.011178782	0.980354196	1.951605513	Nipal1	NIPA-like domain containing 1 [Source:MGI Symbol;Acc:MGI:1917951]
Pdgfd	49.9222747	84.2816377	-0.75	0.013360865	0.980354196	1.874165424	Pdgfd	platelet-derived growth factor, D polypeptide [Source:MGI Symbol;Acc:MGI:1919035]
Mamld1	120.406492	202.463597	-0.75	0.001818101	0.980354196	2.740381994	Mamld1	mastermind-like domain containing 1 [Source:MGI Symbol;Acc:MGI:3045303]
Triqk	22.9744507	38.2445502	-0.74	0.045377986	0.980354196	1.343154783	Triqk	triple QxxK/R motif containing [Source:MGI Symbol;Acc:MGI:3650048]
Igkv1-122	110.020463	183.893383	-0.74	0.01367338	0.980354196	1.864124116	Igkv1-122	immunoglobulin kappa chain variable 1-122 [Source:MGI Symbol;Acc:MGI:4439722]
Fras1	122.357402	204.480718	-0.74	0.005397833	0.980354196	2.267780556	Fras1	Fraser extracellular matrix complex subunit 1 [Source:MGI Symbol;Acc:MGI:2385368]
Ccdc9b	125.547656	209.263309	-0.74	0.022108156	0.980354196	1.65544748	Ccdc9b	coiled-coil domain containing 9B [Source:MGI Symbol;Acc:MGI:2685199]
Sema3a	290.555245	483.525866	-0.73	0.020277074	0.980354196	1.692994714	Sema3a	sema domain, immunoglobulin domain (Ig), short basic domain, secreted, (semaphorin) 3A [Source:MGI Symbol;A
Zbtb20	920.885867	1532.81908	-0.73	0.047260448	0.980354196	1.325502166	Zbtb20	zinc finger and BTB domain containing 20 [Source:MGI Symbol;Acc:MGI:1929213]
Naip6	209.588581	348.525531	-0.73	0.040355877	0.980354196	1.39409321	Naip6	NLR family, apoptosis inhibitory protein 6 [Source:MGI Symbol;Acc:MGI:1298222]
Thrb	59.2325997	98.4118529	-0.73	0.03754172	0.980354196	1.425485834	Thrb	thyroid hormone receptor beta [Source:MGI Symbol;Acc:MGI:98743]
Crispld1	138.800359	230.068134	-0.73	0.007394002	0.980354196	2.131120436	Crispld1	cysteine-rich secretory protein LCCL domain containing 1 [Source:MGI Symbol;Acc:MGI:1934666]
Rab43	213.429629	352.694715	-0.72	0.018779456	0.980354196	1.726316992	Rab43	RAB43, member RAS oncogene family [Source:MGI Symbol;Acc:MGI:1917084]
Cd226	171.722699	283.778296	-0.72	0.001462503	0.980354196	2.834903234	Cd226	CD226 antigen [Source:MGI Symbol;Acc:MGI:3039602]
Dock4	253.502954	417.756038	-0.72	0.018920244	0.980354196	1.723073267	Dock4	dedicator of cytokinesis 4 [Source:MGI Symbol;Acc:MGI:1918006]
Zc3h12c	168.875137	278.417128	-0.72	0.049499908	0.980354196	1.305395608	Zc3h12c	zinc finger CCCH type containing 12C [Source:MGI Symbol;Acc:MGI:3026959]
Ston2	198.706335	326.824023	-0.72	0.004636553	0.980354196	2.333804771	Ston2	stonin 2 [Source:MGI Symbol;Acc:MGI:1918272]
Tet1	93.7744119	154.304396	-0.72	0.041202085	0.980354196	1.385080806	Tet1	tet methylcytosine dioxygenase 1 [Source:MGI Symbol;Acc:MGI:1098693]
Gm19605	59.8650704	98.4475136	-0.71	0.034124609	0.980354196	1.466932316	Gm19605	predicted gene, 19605 [Source:MGI Symbol;Acc:MGI:5011790]
Hunk	32.0808161	52.922189	-0.71	0.047379624	0.980354196	1.32440839	Hunk	hormonally upregulated Neu-associated kinase [Source:MGI Symbol;Acc:MGI:1347352]
Sec31b	89.9794964	147.715744	-0.71	0.032615624	0.980354196	1.486574308	Sec31b	Sec31 homolog B (S. cerevisiae) [Source:MGI Symbol;Acc:MGI:2685187]
Dmxl2	222.347767	363.883083	-0.71	0.009866297	0.980354196	2.005845815	Dmxl2	Dmx-like 2 [Source:MGI Symbol;Acc:MGI:2444630]
Cdk14	340.026708	556.121365	-0.71	0.002470522	0.980354196	2.607211274	Cdk14	cyclin-dependent kinase 14 [Source:MGI Symbol;Acc:MGI:894318]
Kcnj5	44.241678	71.8388111	-0.71	0.013918947	0.980354196	1.856393619	Kcnj5	potassium inwardly-rectifying channel, subfamily J, member 5 [Source:MGI Symbol;Acc:MGI:104755]
Pvrig	36.8896656	60.3847533	-0.71	0.038960329	0.980354196	1.409377384	Pvrig	poliovirus receptor related immunoglobulin domain containing [Source:MGI Symbol;Acc:MGI:5596028]
Pcdh17	211.091156	344.496001	-0.71	0.017643498	0.980354196	1.753415307	Pcdh17	protocadherin 17 [Source:MGI Symbol;Acc:MGI:2684924]
Glis3	89.7104065	146.662065	-0.71	0.038850833	0.980354196	1.410599665	Glis3	GLIS family zinc finger 3 [Source:MGI Symbol;Acc:MGI:2444289]
Pik3c2a	417.334246	677.881346	-0.70	0.036623821	0.980354196	1.436236347	Pik3c2a	phosphatidylinositol-4-phosphate 3-kinase catalytic subunit type 2 alpha [Source:MGI Symbol;Acc:MGI:1203729]
Hs6st2	607.308468	985.926018	-0.70	0.004693098	0.980354196	2.328540377	Hs6st2	heparan sulfate 6-O-sulfotransferase 2 [Source:MGI Symbol;Acc:MGI:1354959]
Arc	62.951797	102.376254	-0.70	0.015662809	0.980354196	1.805130348	Arc	activity regulated cytoskeletal-associated protein [Source:MGI Symbol;Acc:MGI:88067]
Tmem150b	56.2116764	91.6763027	-0.70	0.012216248	0.980354196	1.913062159	Tmem150b	transmembrane protein 150B [Source:MGI Symbol;Acc:MGI:2679718]
Siglec1	914.718341	1481.6594	-0.70	0.046399022	0.980354196	1.333491173	Siglec1	sialic acid binding Ig-like lectin 1, sialoadhesin [Source:MGI Symbol;Acc:MGI:99668]
Wdfy3	687.068986	1106.84372	-0.69	0.02904076	0.980354196	1.536992022	Wdfy3	WD repeat and FYVE domain containing 3 [Source:MGI Symbol;Acc:MGI:1096875]
Col17a1	34.5910553	56.124924	-0.69	0.049266795	0.980354196	1.307445689	Col17a1	collagen, type XVII, alpha 1 [Source:MGI Symbol;Acc:MGI:88450]
Rtl5	138.915994	224.055528	-0.69	0.015376414	0.980354196	1.813144936	Rtl5	retrotransposon Gag like 5 [Source:MGI Symbol;Acc:MGI:3045324]
Adgra2	317.834726	511.187439	-0.68	0.00928332	0.980354196	2.032296679	Adgra2	adhesion G protein-coupled receptor A2 [Source:MGI Symbol;Acc:MGI:1925810]
Dsel	103.593016	166.822711	-0.68	0.00163313	0.980354196	2.786979243	Dsel	dermatan sulfate epimerase-like [Source:MGI Symbol;Acc:MGI:2442948]
Hrh2	117.695458	188.903199	-0.68	0.006747029	0.980354196	2.170887423	Hrh2	histamine receptor H2 [Source:MGI Symbol;Acc:MGI:108482]
Psd3	320.308456	512.652937	-0.68	0.016066094	0.980354196	1.794089696	Psd3	pleckstrin and Sec7 domain containing 3 [Source:MGI Symbol;Acc:MGI:1918215]
Zfp799	112.499799	180.37362	-0.68	0.043409611	0.980354196	1.362414106	Zfp799	zinc finger protein 799 [Source:MGI Symbol;Acc:MGI:2443934]
Filip1	56.8766183	90.9149902	-0.67	0.016158567	0.980354196	1.791597157	Filip1	filamin A interacting protein 1 [Source:MGI Symbol;Acc:MGI:1917848]
Zfp939	34.0538875	54.5037144	-0.67	0.046675705	0.980354196	1.330909114	Zfp939	zinc finger protein 939 [Source:MGI Symbol;Acc:MGI:3036240]
Gm15448	69.3895948	110.99245	-0.67	0.033097355	0.980354196	1.480206712	Gm15448	predicted gene 15448 [Source:MGI Symbol;Acc:MGI:3705216]
Slc17a5	169.080217	269.74588	-0.67	0.006349383	0.980354196	2.197268475	Slc17a5	solute carrier family 17 (anion/sugar transporter), member 5 [Source:MGI Symbol;Acc:MGI:1924105]
Timp3	663.752121	1056.79608	-0.67	0.041395718	0.980354196	1.38304458	Timp3	tissue inhibitor of metalloproteinase 3 [Source:MGI Symbol;Acc:MGI:98754]
Ighv7-3	444.845447	708.111836	-0.67	0.033660215	0.980354196	1.472883114	Ighv7-3	immunoglobulin heavy variable 7-3 [Source:MGI Symbol;Acc:MGI:4439766]
Gvin1	210.536439	334.477954	-0.67	0.019837748	0.980354196	1.702507631	Gvin1	GTPase, very large interferon inducible 1 [Source:MGI Symbol;Acc:MGI:1921808]
Fmn1	99.1501214	157.430965	-0.66	0.038583056	0.980354196	1.413603377	Fmn1	formin 1 [Source:MGI Symbol;Acc:MGI:101815]
Cmtm4	409.379793	647.91155	-0.66	0.031761385	0.980354196	1.498100568	Cmtm4	CKLF-like MARVEL transmembrane domain containing 4 [Source:MGI Symbol;Acc:MGI:2142888]

P2ry10b	189.260181	299.218659	-0.66	0.027677676	0.980354196	1.557870379	P2ry10b	purinergic receptor P2Y, G-protein coupled 10B [Source:MGI Symbol;Acc:MGI:2441814]
Cds1	72.6440637	115.017809	-0.66	0.015637921	0.980354196	1.805820985	Cds1	CDP-diacylglycerol synthase 1 [Source:MGI Symbol;Acc:MGI:1921846]
Prox1	144.19214	227.854352	-0.66	0.029832787	0.980354196	1.525306173	Prox1	prospero homeobox 1 [Source:MGI Symbol;Acc:MGI:97772]
Gm27042	27.5554172	43.6983784	-0.66	0.047514263	0.980354196	1.323176003	Gm27042	predicted gene, 27042 [Source:MGI Symbol;Acc:MGI:5504157]
Pitpnm3	113.188066	178.284466	-0.65	0.025085035	0.980354196	1.600585289	Pitpnm3	PITPNM family member 3 [Source:MGI Symbol;Acc:MGI:2685726]
Lilrb4a	320.559422	502.915987	-0.65	0.031968837	0.980354196	1.495273163	Lilrb4a	leukocyte immunoglobulin-like receptor, subfamily B, member 4A [Source:MGI Symbol;Acc:MGI:102701]
Pdgfc	64.9546551	102.197222	-0.65	0.019947675	0.980354196	1.700107716	Pdgfc	platelet-derived growth factor, C polypeptide [Source:MGI Symbol;Acc:MGI:1859631]
Fam124a	79.1587244	124.359314	-0.65	0.005653584	0.980354196	2.247676151	Fam124a	family with sequence similarity 124, member A [Source:MGI Symbol;Acc:MGI:3645930]
Ighv1-53	807.517644	1264.82546	-0.65	0.006853704	0.980354196	2.164074656	Ighv1-53	immunoglobulin heavy variable 1-53 [Source:MGI Symbol;Acc:MGI:3576502]
Npnt	1596.77959	2499.29994	-0.65	0.010975304	0.980354196	1.959583442	Npnt	nephronectin [Source:MGI Symbol;Acc:MGI:2148811]
Maml3	178.697141	279.890733	-0.65	0.035976746	0.980354196	1.44397812	Maml3	mastermind like transcriptional coactivator 3 [Source:MGI Symbol;Acc:MGI:2389461]
Cacna1e	923.505596	1444.01482	-0.64	0.041373264	0.980354196	1.383280216	Cacna1e	calcium channel, voltage-dependent, R type, alpha 1E subunit [Source:MGI Symbol;Acc:MGI:106217]
Fan1	125.095177	195.230292	-0.64	0.035979627	0.980354196	1.443943343	Fan1	FANCD2/FANCI-associated nuclease 1 [Source:MGI Symbol;Acc:MGI:3045266]
Itga2	176.964677	275.469353	-0.64	0.00500387	0.980354196	2.300693982	Itga2	integrin alpha 2 [Source:MGI Symbol;Acc:MGI:96600]
Fut10	48.6687238	76.0027076	-0.63	0.013329465	0.980354196	1.875187281	Fut10	fucosyltransferase 10 [Source:MGI Symbol;Acc:MGI:2384748]
4921531C22Rik	43.0050414	66.7333554	-0.63	0.02300154	0.980354196	1.638243086	4921531C22Rik	RIKEN cDNA 4921531C22 gene [Source:MGI Symbol;Acc:MGI:1913980]
Robo2	211.707759	328.630916	-0.63	0.046428779	0.980354196	1.333212737	Robo2	roundabout guidance receptor 2 [Source:MGI Symbol;Acc:MGI:1890110]
Ccbe1	200.761173	311.446856	-0.63	0.049350403	0.980354196	1.306709296	Ccbe1	collagen and calcium binding EGF domains 1 [Source:MGI Symbol;Acc:MGI:2445053]
Grk3	1203.39144	1864.14902	-0.63	0.0263704	0.980354196	1.578883283	Grk3	G protein-coupled receptor kinase 3 [Source:MGI Symbol;Acc:MGI:87941]
Ppfibp1	501.823227	776.463367	-0.63	0.02353066	0.980354196	1.628365891	Ppfibp1	PTPRF interacting protein, binding protein 1 (liprin beta 1) [Source:MGI Symbol;Acc:MGI:1914783]
Clic5	843.253041	1303.37725	-0.63	0.023118453	0.980354196	1.636041231	Clic5	chloride intracellular channel 5 [Source:MGI Symbol;Acc:MGI:1917912]
Fhod3	101.207937	156.587625	-0.63	0.027328161	0.980354196	1.563389592	Fhod3	formin homology 2 domain containing 3 [Source:MGI Symbol;Acc:MGI:1925847]
Ldlrad4	128.268213	198.123469	-0.63	0.034884421	0.980354196	1.457368481	Ldlrad4	low density lipoprotein receptor class A domain containing 4 [Source:MGI Symbol;Acc:MGI:1277150]
Adam23	681.094301	1050.79033	-0.62	0.04477679	0.980354196	1.348947044	Adam23	a disintegrin and metallopeptidase domain 23 [Source:MGI Symbol;Acc:MGI:1345162]
Itpripl2	704.137503	1085.07036	-0.62	0.006012022	0.980354196	2.220979439	Itpripl2	inositol 1,4,5-triphosphate receptor interacting protein-like 2 [Source:MGI Symbol;Acc:MGI:2442416]
C130074G19Rik	201.19648	310.288491	-0.62	0.024128168	0.980354196	1.617475652	C130074G19Rik	RIKEN cDNA C130074G19 gene [Source:MGI Symbol;Acc:MGI:2444831]
Smug1	199.152431	306.16635	-0.62	0.01482215	0.980354196	1.829088796	Smug1	single-strand selective monofunctional uracil DNA glycosylase [Source:MGI Symbol;Acc:MGI:1918976]
Sirt5	84.6630214	130.26542	-0.62	0.015704758	0.980354196	1.803968751	Sirt5	sirtuin 5 [Source:MGI Symbol;Acc:MGI:1915596]
Rmdn2	47.1174328	72.3527889	-0.62	0.030209174	0.980354196	1.519861149	Rmdn2	regulator of microtubule dynamics 2 [Source:MGI Symbol;Acc:MGI:2147043]
Shisa2	135.14663	207.580005	-0.62	0.049612446	0.980354196	1.304409361	Shisa2	shisa family member 2 [Source:MGI Symbol;Acc:MGI:2444716]
Plxna1	671.938939	1027.53733	-0.61	0.040463214	0.980354196	1.392939624	Plxna1	plexin A1 [Source:MGI Symbol;Acc:MGI:107685]
E230029C05Rik	125.886698	192.800845	-0.61	0.005422867	0.980354196	2.265771047	E230029C05Rik	RIKEN cDNA E230029C05 gene [Source:MGI Symbol;Acc:MGI:2442580]
Clec9a	422.038095	645.868217	-0.61	0.005785423	0.980354196	2.237664882	Clec9a	C-type lectin domain family 9, member a [Source:MGI Symbol;Acc:MGI:2444608]
Diaph2	334.236956	511.078281	-0.61	0.013639765	0.980354196	1.865193112	Diaph2	diaphanous related formin 2 [Source:MGI Symbol;Acc:MGI:1858500]
Myo9a	1111.52526	1697.32237	-0.61	0.027078645	0.980354196	1.567373071	Myo9a	myosin IXa [Source:MGI Symbol;Acc:MGI:107735]
Srgap3	261.00647	398.924399	-0.61	0.010784084	0.980354196	1.967216738	Srgap3	SLIT-ROBO Rho GTPase activating protein 3 [Source:MGI Symbol;Acc:MGI:2152938]
Rab39	116.083265	177.353697	-0.61	0.04550535	0.980354196	1.341937541	Rab39	RAB39, member RAS oncogene family [Source:MGI Symbol;Acc:MGI:2442855]
Aldh1l1	83.504702	127.543831	-0.60	0.044893618	0.980354196	1.347815393	Aldh1l1	aldehyde dehydrogenase 1 family, member L1 [Source:MGI Symbol;Acc:MGI:1340024]
3-Sep	67.0442083	101.96448	-0.60	0.046675462	0.980354196	1.330911375	3-Sep	septin 3 [Source:MGI Symbol;Acc:MGI:1345148]
Nxpe5	78.1872521	118.356632	-0.60	0.044579619	0.980354196	1.350863648	Nxpe5	neurexophilin and PC-esterase domain family, member 5 [Source:MGI Symbol;Acc:MGI:3584036]
Plekhg1	226.894014	344.683733	-0.60	0.029080492	0.980354196	1.53639825	Plekhg1	pleckstrin homology domain containing, family G (with RhoGef domain) member 1 [Source:MGI Symbol;Acc:MGI:
Rnf180	52.9477352	80.6450123	-0.60	0.047593818	0.980354196	1.322449454	Rnf180	ring finger protein 180 [Source:MGI Symbol;Acc:MGI:1919066]
Vav3	314.914246	477.895675	-0.60	0.001375471	0.980354196	2.861548562	Vav3	vav 3 oncogene [Source:MGI Symbol;Acc:MGI:1888518]
Hpgds	384.253799	582.654923	-0.60	0.004757274	0.980354196	2.322641834	Hpgds	hematopoietic prostaglandin D synthase [Source:MGI Symbol;Acc:MGI:1859384]
Plxnb1	387.606901	586.436874	-0.60	0.040947257	0.980354196	1.387775186	Plxnb1	plexin B1 [Source:MGI Symbol;Acc:MGI:2154238]
Stab2	2411.27053	3644.39812	-0.60	0.022699614	0.980354196	1.643981528	Stab2	stabilin 2 [Source:MGI Symbol;Acc:MGI:2178743]
Mb21d2	51.9829193	78.9891127	-0.60	0.008951974	0.980354196	2.048081188	Mb21d2	Mab-21 domain containing 2 [Source:MGI Symbol;Acc:MGI:1917028]
Zswim7	60.3801303	91.5208689	-0.59	0.023029664	0.980354196	1.637712398	Zswim7	zinc finger SWIM-type containing 7 [Source:MGI Symbol;Acc:MGI:1916997]
Rin2	278.051599	419.505528	-0.59	0.013284804	0.980354196	1.876644849	Rin2	Ras and Rab interactor 2 [Source:MGI Symbol;Acc:MGI:1921280]
Dpy19l4	378.166855	569.875406	-0.59	0.016678633	0.980354196	1.777839548	Dpy19l4	dpy-19-like 4 (C. elegans) [Source:MGI Symbol;Acc:MGI:2685869]
Adra1b	36.2378196	55.0837009	-0.59	0.03620149	0.980354196	1.441273554	Adra1b	adrenergic receptor, alpha 1b [Source:MGI Symbol;Acc:MGI:104774]
Mss51	64.7404832	97.6964059	-0.59	0.045041203	0.980354196	1.346390018	Mss51	MSS51 mitochondrial translational activator [Source:MGI Symbol;Acc:MGI:1922093]
Btbd3	189.084237	284.740278	-0.59	0.01322543	0.980354196	1.878590199	Btbd3	BTB (POZ) domain containing 3 [Source:MGI Symbol;Acc:MGI:2385155]
F5	213.011719	319.245406	-0.59	0.049078556	0.980354196	1.309108224	F5	coagulation factor V [Source:MGI Symbol;Acc:MGI:88382]
Lrrn1	230.832327	346.154615	-0.58	0.015580307	0.980354196	1.807423989	Lrrn1	leucine rich repeat protein 1, neuronal [Source:MGI Symbol;Acc:MGI:106038]
Ddx60	251.515617	376.762017	-0.58	0.04721402	0.980354196	1.32592902	Ddx60	DEAD (Asp-Glu-Ala-Asp) box polypeptide 60 [Source:MGI Symbol;Acc:MGI:2384570]
Zfp772	47.7416696	71.5914169	-0.58	0.039240383	0.980354196	1.406266762	Zfp772	zinc finger protein 772 [Source:MGI Symbol;Acc:MGI:2385265]
Gpr141b	182.903761	273.289137	-0.58	0.04947501	0.980354196	1.305614109	Gpr141b	G protein-coupled receptor 141B [Source:MGI Symbol;Acc:MGI:2441809]
Trio	711.168388	1061.42426	-0.58	0.046224507	0.980354196	1.335127712	Trio	triple functional domain (PTPRF interacting) [Source:MGI Symbol;Acc:MGI:1927230]
Fmnl2	463.145313	690.439861	-0.58	0.022488966	0.980354196	1.648030512	Fmnl2	formin-like 2 [Source:MGI Symbol;Acc:MGI:1918659]
Id4	207.211922	309.335443	-0.57	0.012206377	0.980354196	1.913413221	Id4	inhibitor of DNA binding 4 [Source:MGI Symbol;Acc:MGI:99414]
Olfml1	99.409385	148.398085	-0.57	0.048840951	0.980354196	1.311215888	Olfml1	olfactomedin-like 1 [Source:MGI Symbol;Acc:MGI:2679264]
Sema6d	909.144806	1352.12355	-0.57	0.017336306	0.980354196	1.761043436	Sema6d	sema domain, transmembrane domain (TM), and cytoplasmic domain, (semaphorin) 6D [Source:MGI Symbol;Acc
Gprc5b	132.953407	197.865142	-0.57	0.046777736	0.980354196	1.329960801	Gprc5b	G protein-coupled receptor, family C, group 5, member B [Source:MGI Symbol;Acc:MGI:1927596]
Jam2	215.504873	320.260148	-0.57	0.030521367	0.980354196	1.515396019	Jam2	junction adhesion molecule 2 [Source:MGI Symbol;Acc:MGI:1933820]
Shank3	650.098463	964.056528	-0.57	0.023264824	0.980354196	1.633300229	Shank3	SH3 and multiple ankyrin repeat domains 3 [Source:MGI Symbol;Acc:MGI:1930016]
Gprc5c	154.538379	229.450913	-0.57	0.025919606	0.980354196	1.586371604	Gprc5c	G protein-coupled receptor, family C, group 5, member C [Source:MGI Symbol;Acc:MGI:1917605]
Tubb2b	178.321383	263.928169	-0.57	0.006659661	0.980354196	2.176547877	Tubb2b	tubulin, beta 2B class IIB [Source:MGI Symbol;Acc:MGI:1920960]
Gm28192	64.7267196	95.8805625	-0.56	0.028458051	0.980354196	1.545794847	Gm28192	predicted gene 28192 [Source:MGI Symbol;Acc:MGI:5578898]
Mmrn2	424.833967	628.5303	-0.56	0.020777276	0.980354196	1.682411391	Mmrn2	multimerin 2 [Source:MGI Symbol;Acc:MGI:2385618]
Amotl1	446.375375	660.561895	-0.56	0.039655625	0.980354196	1.401695201	Amotl1	angiomotin-like 1 [Source:MGI Symbol;Acc:MGI:1922973]
Zfp945	261.777225	387.396736	-0.56	0.015118482	0.980354196	1.820491813	Zfp945	zinc finger protein 945 [Source:MGI Symbol;Acc:MGI:2445132]
Klrc1	128.649058	190.852152	-0.56	0.006433941	0.980354196	2.191522926	Klrc1	killer cell lectin-like receptor subfamily C, member 1 [Source:MGI Symbol;Acc:MGI:1336161]
Tmem215	145.104152	214.664112	-0.56	0.025056353	0.980354196	1.601082141	Tmem215	transmembrane protein 215 [Source:MGI Symbol;Acc:MGI:2444167]
Zeb2	1536.94679	2271.30508	-0.56	0.030875444	0.980354196	1.510386788	Zeb2	zinc finger E-box binding homeobox 2 [Source:MGI Symbol;Acc:MGI:1344407]
Pdzd4	109.825207	162.749305	-0.56	0.036164624	0.980354196	1.441716046	Pdzd4	PDZ domain containing 4 [Source:MGI Symbol;Acc:MGI:2443483]
Glis2	170.643946	252.698606	-0.56	0.041445318	0.980354196	1.382524524	Glis2	GLIS family zinc finger 2 [Source:MGI Symbol;Acc:MGI:1932535]
Shroom3	392.797517	580.046399	-0.56	0.030520315	0.980354196	1.515410988	Shroom3	shroom family member 3 [Source:MGI Symbol;Acc:MGI:1351655]
Tanc2	148.40687	219.249701	-0.56	0.037541748	0.980354196	1.42548551	Tanc2	tetratricopeptide repeat, ankyrin repeat and coiled-coil containing 2 [Source:MGI Symbol;Acc:MGI:2444121]
Dclre1c	337.607348	498.339269	-0.56	0.03742438	0.980354196	1.426845386	Dclre1c	DNA cross-link repair 1C [Source:MGI Symbol;Acc:MGI:2441769]

Adgre4	1168.85455	1723.29647	-0.56	0.007915008	0.980354196	2.101548642	Adgre4	adhesion G protein-coupled receptor E4 [Source:MGI Symbol;Acc:MGI:1196464]
Fndc7	100.595376	148.310763	-0.56	0.011102726	0.980354196	1.954570378	Fndc7	fibronectin type III domain containing 7 [Source:MGI Symbol;Acc:MGI:2443535]
Mrc2	200.759959	296.219533	-0.56	0.017328	0.980354196	1.761251561	Mrc2	mannose receptor, C type 2 [Source:MGI Symbol;Acc:MGI:107818]
Lrp1	4256.2897	6259.26122	-0.56	0.037065595	0.980354196	1.431029024	Lrp1	low density lipoprotein receptor-related protein 1 [Source:MGI Symbol;Acc:MGI:96828]
Arhgap29	620.68414	912.463758	-0.56	0.035919075	0.980354196	1.444674856	Arhgap29	Rho GTPase activating protein 29 [Source:MGI Symbol;Acc:MGI:2443818]
Otud3	99.57445	146.813736	-0.55	0.038884967	0.980354196	1.410218265	Otud3	OTU domain containing 3 [Source:MGI Symbol;Acc:MGI:1920412]
Exoc3l4	209.792113	308.445185	-0.55	0.019149482	0.980354196	1.717842969	Exoc3l4	exocyst complex component 3-like 4 [Source:MGI Symbol;Acc:MGI:1921363]
Gm37494	555.816185	815.692125	-0.55	0.024527518	0.980354196	1.610346397	Gm37494	predicted gene, 37494 [Source:MGI Symbol;Acc:MGI:5610722]
Pex1	177.758199	261.296616	-0.55	0.02498609	0.980354196	1.6023017	Pex1	peroxisomal biogenesis factor 1 [Source:MGI Symbol;Acc:MGI:1918632]
5330417C22Rik	100.259379	147.308752	-0.55	0.033925918	0.980354196	1.469468392	5330417C22Rik	RIKEN cDNA 5330417C22 gene [Source:MGI Symbol;Acc:MGI:1923930]
Siglech	427.430144	626.364264	-0.55	0.049769494	0.980354196	1.303036775	Siglech	sialic acid binding Ig-like lectin H [Source:MGI Symbol;Acc:MGI:2443256]
Col8a2	240.45506	352.896785	-0.55	0.016818438	0.980354196	1.774214341	Col8a2	collagen, type VIII, alpha 2 [Source:MGI Symbol;Acc:MGI:88464]
Kazn	331.115922	483.943623	-0.55	0.003562357	0.980354196	2.44826256	Kazn	kazrin, periplakin interacting protein [Source:MGI Symbol;Acc:MGI:1918779]
Syt15	116.517995	169.623369	-0.54	0.020255037	0.980354196	1.693466959	Syt15	synaptotagmin XV [Source:MGI Symbol;Acc:MGI:2442166]
Sorbs2	670.929253	976.194373	-0.54	0.037483634	0.980354196	1.426158311	Sorbs2	sorbin and SH3 domain containing 2 [Source:MGI Symbol;Acc:MGI:1924574]
Nhlrc3	414.997443	603.999935	-0.54	0.028837373	0.980354196	1.540044305	Nhlrc3	NHL repeat containing 3 [Source:MGI Symbol;Acc:MGI:2444520]
Spns3	284.937765	413.87956	-0.54	0.004635992	0.980354196	2.333857322	Spns3	spinster homolog 3 [Source:MGI Symbol;Acc:MGI:1924827]
Met	307.273809	445.7508	-0.54	0.037127686	0.980354196	1.430302118	Met	met proto-oncogene [Source:MGI Symbol;Acc:MGI:96969]
Pard3b	102.620319	148.927172	-0.53	0.036000204	0.980354196	1.443695038	Pard3b	par-3 family cell polarity regulator beta [Source:MGI Symbol;Acc:MGI:1919301]
Pde5a	195.079989	281.470058	-0.53	0.002576433	0.980354196	2.588981147	Pde5a	phosphodiesterase 5A, cGMP-specific [Source:MGI Symbol;Acc:MGI:2651499]
Slc38a6	170.55893	246.788255	-0.53	0.031003946	0.980354196	1.508583028	Slc38a6	solute carrier family 38, member 6 [Source:MGI Symbol;Acc:MGI:3648156]
Vangl2	152.236173	220.398207	-0.53	0.027308591	0.980354196	1.563700707	Vangl2	VANGL planar cell polarity 2 [Source:MGI Symbol;Acc:MGI:2135272]
Cdc42bpb	642.5292	928.987812	-0.53	0.048735392	0.980354196	1.312155536	Cdc42bpb	CDC42 binding protein kinase beta [Source:MGI Symbol;Acc:MGI:2136459]
Slc6a6	2007.51739	2901.50644	-0.53	0.034448904	0.980354196	1.462824591	Slc6a6	solute carrier family 6 (neurotransmitter transporter, taurine), member 6 [Source:MGI Symbol;Acc:MGI:98488]
Lnpep	3608.55818	5211.77235	-0.53	0.026257619	0.980354196	1.580744658	Lnpep	leucyl/cystinyl aminopeptidase [Source:MGI Symbol;Acc:MGI:2387123]
Srr	124.177975	179.460127	-0.53	0.041788114	0.980354196	1.378947229	Srr	serine racemase [Source:MGI Symbol;Acc:MGI:1351636]
Il18rap	384.447601	553.866694	-0.53	0.018287771	0.980354196	1.737839225	Il18rap	interleukin 18 receptor accessory protein [Source:MGI Symbol;Acc:MGI:1338888]
Tmem38a	52.8351615	75.9932796	-0.53	0.028736097	0.980354196	1.541572219	Tmem38a	transmembrane protein 38A [Source:MGI Symbol;Acc:MGI:1921416]
Tmem170b	583.98736	841.652912	-0.53	0.044507192	0.980354196	1.351569805	Tmem170b	transmembrane protein 170B [Source:MGI Symbol;Acc:MGI:3647046]
Sgcb	213.002004	307.392875	-0.53	0.014635937	0.980354196	1.834579469	Sgcb	sarcoglycan, beta (dystrophin-associated glycoprotein) [Source:MGI Symbol;Acc:MGI:1346523]
Tns3	3027.57501	4356.74825	-0.52	0.038305945	0.980354196	1.416733819	Tns3	tensin 3 [Source:MGI Symbol;Acc:MGI:2443012]
B230307C23Rik	94.0174013	135.080625	-0.52	0.007163212	0.980354196	2.144892195	B230307C23Rik	RIKEN cDNA B230307C23 gene [Source:MGI Symbol;Acc:MGI:3643396]
Dcbld1	74.003965	106.448528	-0.52	0.008609793	0.980354196	2.06500729	Dcbld1	discoidin, CUB and LCCL domain containing 1 [Source:MGI Symbol;Acc:MGI:1913936]
Amigo3	140.065519	201.536297	-0.52	0.032076203	0.980354196	1.493817047	Amigo3	adhesion molecule with Ig like domain 3 [Source:MGI Symbol;Acc:MGI:2444854]
Abca1	1678.88601	2411.75094	-0.52	0.046649796	0.980354196	1.331150251	Abca1	ATP-binding cassette, sub-family A (ABC1), member 1 [Source:MGI Symbol;Acc:MGI:99607]
Tmem26	499.644219	717.6535	-0.52	0.0079044	0.980354196	2.102131091	Tmem26	transmembrane protein 26 [Source:MGI Symbol;Acc:MGI:2143537]
Efnb2	296.335772	425.746554	-0.52	0.027319806	0.980354196	1.563522389	Efnb2	ephrin B2 [Source:MGI Symbol;Acc:MGI:105097]
Klrk1	482.617583	691.57619	-0.52	0.002620235	0.980354196	2.581659757	Klrk1	killer cell lectin-like receptor subfamily K, member 1 [Source:MGI Symbol;Acc:MGI:1196250]
Ccdc80	705.265114	1008.84699	-0.52	0.008402874	0.980354196	2.075572149	Ccdc80	coiled-coil domain containing 80 [Source:MGI Symbol;Acc:MGI:1915146]
I830077J02Rik	679.499251	971.097281	-0.51	0.024252217	0.980354196	1.615248555	I830077J02Rik	RIKEN cDNA I830077J02 gene [Source:MGI Symbol;Acc:MGI:3588284]
Plcb4	221.167396	316.058309	-0.51	0.017954507	0.980354196	1.745826515	Plcb4	phospholipase C, beta 4 [Source:MGI Symbol;Acc:MGI:107464]
Synpo2	912.808273	1303.24452	-0.51	0.032412558	0.980354196	1.489286693	Synpo2	synaptopodin 2 [Source:MGI Symbol;Acc:MGI:2153070]
Mycl	378.212554	540.040684	-0.51	0.001941385	0.980354196	2.71188833	Mycl	v-myc avian myelocytomatosis viral oncogene lung carcinoma derived [Source:MGI Symbol;Acc:MGI:96799]
Cirbp	1214.83142	1731.75248	-0.51	0.007770937	0.980354196	2.109526612	Cirbp	cold inducible RNA binding protein [Source:MGI Symbol;Acc:MGI:893588]
Cd209b	275.624397	392.103694	-0.51	0.039367754	0.980354196	1.404859362	Cd209b	CD209b antigen [Source:MGI Symbol;Acc:MGI:1916415]
Gm26541	93.2622129	132.909871	-0.51	0.04224184	0.980354196	1.374257173	Gm26541	predicted gene, 26541 [Source:MGI Symbol;Acc:MGI:5477035]
Plekha6	1150.16448	1632.13667	-0.50	0.048442288	0.980354196	1.314775353	Plekha6	pleckstrin homology domain containing, family A member 6 [Source:MGI Symbol;Acc:MGI:2388662]
Sspn	213.619212	303.31061	-0.50	0.039885263	0.980354196	1.39918754	Sspn	sarcospan [Source:MGI Symbol;Acc:MGI:1353511]
Pde3a	395.546787	559.854715	-0.50	0.034454008	0.980354196	1.46276025	Pde3a	phosphodiesterase 3A, cGMP inhibited [Source:MGI Symbol;Acc:MGI:1860764]
Rab11fip3	287.013088	406.338326	-0.50	0.009007482	0.980354196	2.045396597	Rab11fip3	RAB11 family interacting protein 3 (class II) [Source:MGI Symbol;Acc:MGI:2444431]
Rab11fip5	415.173487	586.799585	-0.50	0.025324629	0.980354196	1.596456908	Rab11fip5	RAB11 family interacting protein 5 (class I) [Source:MGI Symbol;Acc:MGI:1098586]
Flt3	788.072135	1113.93436	-0.50	0.011232233	0.980354196	1.949533896	Flt3	FMS-like tyrosine kinase 3 [Source:MGI Symbol;Acc:MGI:95559]
Parva	466.991331	657.651835	-0.49	0.016697651	0.980354196	1.77734462	Parva	parvin, alpha [Source:MGI Symbol;Acc:MGI:1931144]
Serhl	134.913535	189.647102	-0.49	0.034301917	0.980354196	1.464681608	Serhl	serine hydrolase-like [Source:MGI Symbol;Acc:MGI:1890404]
Gpsm1	251.720798	354.344309	-0.49	0.015088953	0.980354196	1.821340894	Gpsm1	G-protein signalling modulator 1 (AGS3-like, C. elegans) [Source:MGI Symbol;Acc:MGI:1915089]
Bmf	582.432244	818.731824	-0.49	0.010962119	0.980354196	1.960105488	Bmf	BCL2 modifying factor [Source:MGI Symbol;Acc:MGI:2176433]
Fgfr3	305.65508	429.49823	-0.49	0.04404755	0.980354196	1.356078243	Fgfr3	fibroblast growth factor receptor 3 [Source:MGI Symbol;Acc:MGI:95524]
Itga9	957.67214	1344.06542	-0.49	0.033336218	0.980354196	1.477083673	Itga9	integrin alpha 9 [Source:MGI Symbol;Acc:MGI:104756]
Gm30211	311.934521	436.849439	-0.48	0.048195105	0.980354196	1.316997069	Gm30211	predicted gene, 30211 [Source:MGI Symbol;Acc:MGI:5589370]
Mtss1l	658.904214	922.14711	-0.48	0.038890356	0.980354196	1.410158081	Mtss1l	metastasis suppressor 1-like [Source:MGI Symbol;Acc:MGI:3039591]
Tspan9	506.354736	708.325123	-0.48	0.011953255	0.980354196	1.922513816	Tspan9	tetraspanin 9 [Source:MGI Symbol;Acc:MGI:1924558]
Fzd1	762.130983	1065.59473	-0.48	0.047483917	0.980354196	1.323453463	Fzd1	frizzled class receptor 1 [Source:MGI Symbol;Acc:MGI:1196625]
Pdgfrb	1022.00009	1427.96932	-0.48	0.044189738	0.980354196	1.354678573	Pdgfrb	platelet derived growth factor receptor, beta polypeptide [Source:MGI Symbol;Acc:MGI:97531]
Cplx2	204.04123	285.109488	-0.48	0.033075826	0.980354196	1.480489301	Cplx2	complexin 2 [Source:MGI Symbol;Acc:MGI:104726]
Rhbdd1	328.827147	458.455729	-0.48	0.019483445	0.980354196	1.71033425	Rhbdd1	rhomboid domain containing 1 [Source:MGI Symbol;Acc:MGI:1924117]
Trpm2	1617.7511	2250.62683	-0.48	0.031478931	0.980354196	1.501980024	Trpm2	transient receptor potential cation channel, subfamily M, member 2 [Source:MGI Symbol;Acc:MGI:1351901]
Relch	692.027838	963.027096	-0.48	0.032675587	0.980354196	1.485776602	Relch	RAB11 binding and LisH domain, coiled-coil and HEAT repeat containing [Source:MGI Symbol;Acc:MGI:1922832]
Cavin2	906.89444	1260.70679	-0.47	0.013594037	0.980354196	1.866651552	Cavin2	caveolae associated 2 [Source:MGI Symbol;Acc:MGI:99513]
Lacc1	264.182731	367.558572	-0.47	0.024718148	0.980354196	1.606984072	Lacc1	laccase domain containing 1 [Source:MGI Symbol;Acc:MGI:2445077]
Fam214a	771.582354	1070.38513	-0.47	0.026967712	0.980354196	1.569155898	Fam214a	family with sequence similarity 214, member A [Source:MGI Symbol;Acc:MGI:2387648]
Fgf1	334.581159	463.272309	-0.47	0.02188401	0.980354196	1.659873095	Fgf1	fibroblast growth factor 1 [Source:MGI Symbol;Acc:MGI:95515]
Npl	132.891074	184.352204	-0.47	0.023414166	0.980354196	1.630521307	Npl	N-acetylneuraminate pyruvate lyase [Source:MGI Symbol;Acc:MGI:1921341]
Mfsd4b4	101.917925	141.418321	-0.47	0.027724461	0.980354196	1.557136888	Mfsd4b4	major facilitator superfamily domain containing 4B4 [Source:MGI Symbol;Acc:MGI:3035041]
Mthfr	468.355329	648.004052	-0.47	0.040308568	0.980354196	1.39460263	Mthfr	methylenetetrahydrofolate reductase [Source:MGI Symbol;Acc:MGI:106639]
Lair1	639.967057	884.394546	-0.47	0.003509799	0.980354196	2.454717754	Lair1	leukocyte-associated Ig-like receptor 1 [Source:MGI Symbol;Acc:MGI:105492]
Hip1	883.526013	1220.00857	-0.47	0.028297446	0.980354196	1.54825276	Hip1	huntingtin interacting protein 1 [Source:MGI Symbol;Acc:MGI:1099804]
Ccdc148	109.457548	150.444469	-0.46	0.042011831	0.980354196	1.37662839	Ccdc148	coiled-coil domain containing 148 [Source:MGI Symbol;Acc:MGI:3039583]
Aldh1b1	158.24594	217.445458	-0.46	0.003060734	0.980354196	2.514174412	Aldh1b1	aldehyde dehydrogenase 1 family, member B1 [Source:MGI Symbol;Acc:MGI:1919785]
Il18r1	374.179705	515.224458	-0.46	0.044354886	0.980354196	1.353058533	Il18r1	interleukin 18 receptor 1 [Source:MGI Symbol;Acc:MGI:105383]
Mmrn1	161.451935	221.893884	-0.46	0.030622648	0.980354196	1.513957258	Mmrn1	multimerin 1 [Source:MGI Symbol;Acc:MGI:1918195]

Arl13b	195.715796	269.613464	-0.46	0.011448942	0.980354196	1.941234645	Arl13b	ADP-ribosylation factor-like 13B [Source:MGI Symbol;Acc:MGI:1915396]
2510009E07Rik	287.647549	395.170034	-0.46	0.025733337	0.980354196	1.589503893	2510009E07Rik	RIKEN cDNA 2510009E07 gene [Source:MGI Symbol;Acc:MGI:1919440]
Rims3	519.322459	711.538108	-0.45	0.032679342	0.980354196	1.485726697	Rims3	regulating synaptic membrane exocytosis 3 [Source:MGI Symbol;Acc:MGI:2443331]
Dnmt3a	1636.51308	2241.03548	-0.45	0.030783102	0.980354196	1.511687619	Dnmt3a	DNA methyltransferase 3A [Source:MGI Symbol;Acc:MGI:1261827]
Plpp3	716.392988	980.872356	-0.45	0.046982234	0.980354196	1.328066336	Plpp3	phospholipid phosphatase 3 [Source:MGI Symbol;Acc:MGI:1915166]
Ccr2	834.188346	1139.79981	-0.45	0.030105986	0.980354196	1.521347145	Ccr2	chemokine (C-C motif) receptor 2 [Source:MGI Symbol;Acc:MGI:106185]
Gpr35	394.578514	539.146753	-0.45	0.010492199	0.980354196	1.979133481	Gpr35	G protein-coupled receptor 35 [Source:MGI Symbol;Acc:MGI:1929509]
Mfap3l	69.379781	94.3383896	-0.45	0.023794125	0.980354196	1.623530261	Mfap3l	microfibrillar-associated protein 3-like [Source:MGI Symbol;Acc:MGI:1918556]
Ptpre	613.808687	838.268639	-0.45	0.011949125	0.980354196	1.922663896	Ptpre	protein tyrosine phosphatase, receptor type, E [Source:MGI Symbol;Acc:MGI:97813]
Socs2	195.554437	267.649477	-0.45	0.011585604	0.980354196	1.93608132	Socs2	suppressor of cytokine signaling 2 [Source:MGI Symbol;Acc:MGI:1201787]
Eps8	314.47224	429.257042	-0.45	0.037011	0.980354196	1.431669181	Eps8	epidermal growth factor receptor pathway substrate 8 [Source:MGI Symbol;Acc:MGI:104684]
Ankrd50	860.459953	1170.42593	-0.44	0.045778771	0.980354196	1.339335871	Ankrd50	ankyrin repeat domain 50 [Source:MGI Symbol;Acc:MGI:2139777]
Phldb1	303.315191	412.206286	-0.44	0.049988062	0.980354196	1.3011337	Phldb1	pleckstrin homology like domain, family B, member 1 [Source:MGI Symbol;Acc:MGI:2143230]
Stab1	727.06207	987.038647	-0.44	0.013422944	0.980354196	1.872152222	Stab1	stabilin 1 [Source:MGI Symbol;Acc:MGI:2178742]
Itgad	1265.48008	1715.90881	-0.44	0.009803557	0.980354196	2.008616322	Itgad	integrin, alpha D [Source:MGI Symbol;Acc:MGI:3578624]
Zfp763	112.865273	153.355301	-0.44	0.041342709	0.980354196	1.383601069	Zfp763	zinc finger protein 763 [Source:MGI Symbol;Acc:MGI:1920701]
Gsdme	223.7635	303.269382	-0.44	0.015138908	0.980354196	1.81990545	Gsdme	gasdermin E [Source:MGI Symbol;Acc:MGI:1889850]
Ifi207	456.623254	618.244498	-0.44	0.019519088	0.980354196	1.709540478	Ifi207	interferon activated gene 207 [Source:MGI Symbol;Acc:MGI:2138302]
Ccdc8	160.855348	217.76888	-0.44	0.040326063	0.980354196	1.394414176	Ccdc8	coiled-coil domain containing 8 [Source:MGI Symbol;Acc:MGI:3612184]
Mrc1	2236.95879	3026.29292	-0.44	0.014561773	0.980354196	1.836785743	Mrc1	mannose receptor, C type 1 [Source:MGI Symbol;Acc:MGI:97142]
Vill	143.252438	193.528733	-0.44	0.035454001	0.980354196	1.450334747	Vill	villin-like [Source:MGI Symbol;Acc:MGI:1201781]
Cuedc1	295.603736	399.416055	-0.43	0.044196517	0.980354196	1.354611955	Cuedc1	CUE domain containing 1 [Source:MGI Symbol;Acc:MGI:2144281]
Fam102b	522.438184	705.515614	-0.43	0.008040637	0.980354196	2.094709544	Fam102b	family with sequence similarity 102, member B [Source:MGI Symbol;Acc:MGI:3036259]
Pcdh12	549.872682	741.392142	-0.43	0.043566357	0.980354196	1.360848754	Pcdh12	protocadherin 12 [Source:MGI Symbol;Acc:MGI:1855700]
Sema3g	300.317289	404.98252	-0.43	0.047465336	0.980354196	1.32362344	Sema3g	sema domain, immunoglobulin domain (Ig), short basic domain, secreted, (semaphorin) 3G [Source:MGI Symbol;A
Frmd4b	1084.87466	1460.38863	-0.43	0.019239119	0.980354196	1.715814819	Frmd4b	FERM domain containing 4B [Source:MGI Symbol;Acc:MGI:2141794]
Lmbrd2	586.451854	788.877044	-0.43	0.035459551	0.980354196	1.450266768	Lmbrd2	LMBR1 domain containing 2 [Source:MGI Symbol;Acc:MGI:2444173]
Ppfia4	470.666188	632.714842	-0.43	0.002482868	0.980354196	2.605046369	Ppfia4	protein tyrosine phosphatase, receptor type, f polypeptide (PTPRF), interacting protein (liprin), alpha 4 [Source:M
Ccl6	647.167586	868.227536	-0.43	0.011704717	0.980354196	1.931639082	Ccl6	chemokine (C-C motif) ligand 6 [Source:MGI Symbol;Acc:MGI:98263]
Htra3	358.919173	481.8293	-0.42	0.047522161	0.980354196	1.323103819	Htra3	HtrA serine peptidase 3 [Source:MGI Symbol;Acc:MGI:1925808]
Anpep	728.980344	977.609662	-0.42	0.003900838	0.980354196	2.408842085	Anpep	alanyl (membrane) aminopeptidase [Source:MGI Symbol;Acc:MGI:5000466]
Adgre1	1655.43624	2217.22808	-0.42	0.024404396	0.980354196	1.612531937	Adgre1	adhesion G protein-coupled receptor E1 [Source:MGI Symbol;Acc:MGI:106912]
Sdc3	5991.94455	8023.45425	-0.42	0.009431023	0.980354196	2.025441196	Sdc3	syndecan 3 [Source:MGI Symbol;Acc:MGI:1349163]
Fbxw17	157.981057	211.974968	-0.42	0.037970101	0.980354196	1.420558248	Fbxw17	F-box and WD-40 domain protein 17 [Source:MGI Symbol;Acc:MGI:1923584]
Cpne3	844.74928	1130.54593	-0.42	0.025255632	0.980354196	1.597641759	Cpne3	copine III [Source:MGI Symbol;Acc:MGI:1917818]
Tubb1	322.320452	430.321849	-0.42	0.026266384	0.980354196	1.580599711	Tubb1	tubulin, beta 1 class VI [Source:MGI Symbol;Acc:MGI:107814]
Arhgef10	220.555009	295.181998	-0.42	0.039305269	0.980354196	1.405549227	Arhgef10	Rho guanine nucleotide exchange factor (GEF) 10 [Source:MGI Symbol;Acc:MGI:2444453]
Snx21	277.893531	371.750831	-0.42	0.031623413	0.980354196	1.49999126	Snx21	sorting nexin family member 21 [Source:MGI Symbol;Acc:MGI:1917729]
Klf9	273.483252	364.898116	-0.41	0.048441623	0.980354196	1.314781315	Klf9	Kruppel-like factor 9 [Source:MGI Symbol;Acc:MGI:1333856]
Myom1	186.280256	248.740411	-0.41	0.045968157	0.980354196	1.337542908	Myom1	myomesin 1 [Source:MGI Symbol;Acc:MGI:1341430]
Slc40a1	7184.89321	9568.52908	-0.41	0.035723262	0.980354196	1.447048891	Slc40a1	solute carrier family 40 (iron-regulated transporter), member 1 [Source:MGI Symbol;Acc:MGI:1315204]
Msrb3	332.299248	442.407541	-0.41	0.021868863	0.980354196	1.660173796	Msrb3	methionine sulfoxide reductase B3 [Source:MGI Symbol;Acc:MGI:2443538]
Stx3	168.468557	224.362245	-0.41	0.003707909	0.980354196	2.430870933	Stx3	syntaxin 3 [Source:MGI Symbol;Acc:MGI:103077]
Man1c1	383.496353	509.444233	-0.41	0.009111486	0.980354196	2.040410788	Man1c1	mannosidase, alpha, class 1C, member 1 [Source:MGI Symbol;Acc:MGI:2446214]
Sema3f	620.290642	824.067016	-0.41	0.028583195	0.980354196	1.543889228	Sema3f	sema domain, immunoglobulin domain (Ig), short basic domain, secreted, (semaphorin) 3F [Source:MGI Symbol;A
Ltbr	444.133313	589.860579	-0.41	0.003527221	0.980354196	2.452567329	Ltbr	lymphotoxin B receptor [Source:MGI Symbol;Acc:MGI:104875]
Plekhg5	253.204212	336.449071	-0.41	0.007234825	0.980354196	2.140571969	Plekhg5	pleckstrin homology domain containing, family G (with RhoGef domain) member 5 [Source:MGI Symbol;Acc:MGI:
Etv5	372.244099	493.871435	-0.41	0.047875434	0.980354196	1.319887276	Etv5	ets variant 5 [Source:MGI Symbol;Acc:MGI:1096867]
Slc43a2	1554.73462	2060.68651	-0.41	0.030659284	0.980354196	1.513437992	Slc43a2	solute carrier family 43, member 2 [Source:MGI Symbol;Acc:MGI:2442746]
Rnf144b	388.014792	513.712409	-0.40	0.010008351	0.980354196	1.999637472	Rnf144b	ring finger protein 144B [Source:MGI Symbol;Acc:MGI:2384986]
Tep1	1247.75604	1651.15463	-0.40	0.048683621	0.980354196	1.312617127	Tep1	telomerase associated protein 1 [Source:MGI Symbol;Acc:MGI:109573]
Rilpl1	143.598584	190.280155	-0.40	0.010262545	0.980354196	1.988744926	Rilpl1	Rab interacting lysosomal protein-like 1 [Source:MGI Symbol;Acc:MGI:1922945]
Ptgir	188.429727	249.176662	-0.40	0.014573699	0.980354196	1.836430204	Ptgir	prostaglandin I receptor (IP) [Source:MGI Symbol;Acc:MGI:99535]
Ctso	1242.10418	1640.36383	-0.40	0.031238929	0.980354196	1.505303864	Ctso	cathepsin O [Source:MGI Symbol;Acc:MGI:2139628]
Rtn4rl1	359.493734	474.888312	-0.40	0.018067853	0.980354196	1.743093452	Rtn4rl1	reticulon 4 receptor-like 1 [Source:MGI Symbol;Acc:MGI:2661375]
Pura	475.023691	626.944166	-0.40	0.025084496	0.980354196	1.60059462	Pura	purine rich element binding protein A [Source:MGI Symbol;Acc:MGI:103079]
P2ry13	826.36656	1089.28713	-0.40	0.041647364	0.980354196	1.380412481	P2ry13	purinergic receptor P2Y, G-protein coupled 13 [Source:MGI Symbol;Acc:MGI:1921441]
Rgs18	226.874687	298.746669	-0.40	0.023191722	0.980354196	1.634667004	Rgs18	regulator of G-protein signaling 18 [Source:MGI Symbol;Acc:MGI:1927498]
Arsb	341.283497	448.970624	-0.40	0.031206926	0.980354196	1.505749009	Arsb	arylsulfatase B [Source:MGI Symbol;Acc:MGI:88075]
Cflar	2211.33231	2905.27115	-0.39	0.049514437	0.980354196	1.305268155	Cflar	CASP8 and FADD-like apoptosis regulator [Source:MGI Symbol;Acc:MGI:1336166]
Gabra3	311.378474	408.849989	-0.39	0.019643267	0.980354196	1.70678628	Gabra3	gamma-aminobutyric acid (GABA) A receptor, subunit alpha 3 [Source:MGI Symbol;Acc:MGI:95615]
Bmp6	253.086347	332.898719	-0.39	0.02679734	0.980354196	1.571908313	Bmp6	bone morphogenetic protein 6 [Source:MGI Symbol;Acc:MGI:88182]
Hap1	1002.08731	1316.02999	-0.39	0.042968731	0.980354196	1.366847472	Hap1	huntingtin-associated protein 1 [Source:MGI Symbol;Acc:MGI:1261831]
Pcolce2	182.08062	238.491255	-0.39	0.037800694	0.980354196	1.422500227	Pcolce2	procollagen C-endopeptidase enhancer 2 [Source:MGI Symbol;Acc:MGI:1923727]
Klra4	98.3332978	128.956324	-0.39	0.042057148	0.980354196	1.376160181	Klra4	killer cell lectin-like receptor, subfamily A, member 4 [Source:MGI Symbol;Acc:MGI:101904]
Kcnj10	1023.23628	1337.67363	-0.39	0.047521948	0.980354196	1.323105765	Kcnj10	potassium inwardly-rectifying channel, subfamily J, member 10 [Source:MGI Symbol;Acc:MGI:1194504]
Rap1gap2	694.589105	906.700512	-0.38	0.041430449	0.980354196	1.38268036	Rap1gap2	RAP1 GTPase activating protein 2 [Source:MGI Symbol;Acc:MGI:3028623]
5730480H06Rik	154.655911	202.152177	-0.38	0.038231589	0.980354196	1.417577651	5730480H06Rik	RIKEN cDNA 5730480H06 gene [Source:MGI Symbol;Acc:MGI:1917842]
Zdhhc21	636.16016	830.433198	-0.38	0.049078443	0.980354196	1.309109224	Zdhhc21	zinc finger, DHHC domain containing 21 [Source:MGI Symbol;Acc:MGI:1915518]
Nos3	162.674143	212.462446	-0.38	0.036798232	0.980354196	1.434173047	Nos3	nitric oxide synthase 3, endothelial cell [Source:MGI Symbol;Acc:MGI:97362]
C1qtnf1	258.542457	336.853739	-0.38	0.027510373	0.980354196	1.560503521	C1qtnf1	C1q and tumor necrosis factor related protein 1 [Source:MGI Symbol;Acc:MGI:1919254]
H6pd	1297.10176	1688.36789	-0.38	0.028924794	0.980354196	1.538729725	H6pd	hexose-6-phosphate dehydrogenase (glucose 1-dehydrogenase) [Source:MGI Symbol;Acc:MGI:2140356]
Ctbp2	220.226053	286.392351	-0.38	0.027632029	0.980354196	1.558587224	Ctbp2	C-terminal binding protein 2 [Source:MGI Symbol;Acc:MGI:1201686]
Col14a1	1991.90154	2591.64126	-0.38	0.038594409	0.980354196	1.413475605	Col14a1	collagen, type XIV, alpha 1 [Source:MGI Symbol;Acc:MGI:1341272]
Tmem110	446.627679	580.225421	-0.38	0.007495097	0.980354196	2.125222742	Tmem110	transmembrane protein 110 [Source:MGI Symbol;Acc:MGI:1921500]
6530402F18Rik	270.602212	351.529852	-0.38	0.02027829	0.980354196	1.69296867	6530402F18Rik	RIKEN cDNA 6530402F18 gene [Source:MGI Symbol;Acc:MGI:1923470]
Zfp513	464.10757	601.697884	-0.37	0.027317768	0.980354196	1.563554788	Zfp513	zinc finger protein 513 [Source:MGI Symbol;Acc:MGI:2141255]
Mpeg1	8705.59898	11276.8886	-0.37	0.035473527	0.980354196	1.450095629	Mpeg1	macrophage expressed gene 1 [Source:MGI Symbol;Acc:MGI:1333743]
Phf11d	148.297705	192.398279	-0.37	0.022072309	0.980354196	1.656152233	Phf11d	PHD finger protein 11D [Source:MGI Symbol;Acc:MGI:1277133]
Fzd7	208.43115	269.924736	-0.37	0.042858202	0.980354196	1.367966053	Fzd7	frizzled class receptor 7 [Source:MGI Symbol;Acc:MGI:108570]

Lgalsl	283.306927	366.93359	-0.37	0.027847883	0.980354196	1.555207814	Lgalsl	lectin, galactoside binding-like [Source:MGI Symbol;Acc:MGI:1916114]
Palm	327.403702	422.492678	-0.37	0.032285599	0.980354196	1.490991152	Palm	paralemmin [Source:MGI Symbol;Acc:MGI:1261814]
Tlx1	583.124032	752.987596	-0.37	0.02253153	0.980354196	1.647209317	Tlx1	T cell leukemia, homeobox 1 [Source:MGI Symbol;Acc:MGI:98769]
Sema6b	218.834339	282.130427	-0.37	0.032441469	0.980354196	1.488899488	Sema6b	sema domain, transmembrane domain (TM), and cytoplasmic domain, (semaphorin) 6B [Source:MGI Symbol;Acc:
Ptgs1	744.393326	959.087156	-0.37	0.03700641	0.980354196	1.431723044	Ptgs1	prostaglandin-endoperoxide synthase 1 [Source:MGI Symbol;Acc:MGI:97797]
Gsap	676.913873	872.259323	-0.36	0.048111207	0.980354196	1.317753748	Gsap	gamma-secretase activating protein [Source:MGI Symbol;Acc:MGI:2442259]
Havcr2	481.696538	619.718679	-0.36	0.034627805	0.980354196	1.460575037	Havcr2	hepatitis A virus cellular receptor 2 [Source:MGI Symbol;Acc:MGI:2159682]
Abcg3	1817.28556	2335.70146	-0.36	0.037440991	0.980354196	1.426652665	Abcg3	ATP binding cassette subfamily G member 3 [Source:MGI Symbol;Acc:MGI:1351624]
Zfp790	289.495327	372.627136	-0.36	0.045143906	0.980354196	1.345400867	Zfp790	zinc finger protein 790 [Source:MGI Symbol;Acc:MGI:1923431]
Vwa5a	835.761994	1072.83735	-0.36	0.027187821	0.980354196	1.565625598	Vwa5a	von Willebrand factor A domain containing 5A [Source:MGI Symbol;Acc:MGI:1915026]
Adamts5	411.788768	528.297924	-0.36	0.029415616	0.980354196	1.531422053	Adamts5	a disintegrin-like and metallopeptidase (reprolysin type) with thrombospondin type 1 motif, 5 (aggrecanase-2) [Sc
Ddr1	721.82216	924.950211	-0.36	0.03108244	0.980354196	1.507484896	Ddr1	discoidin domain receptor family, member 1 [Source:MGI Symbol;Acc:MGI:99216]
Nabp1	918.166852	1175.77775	-0.36	0.048316981	0.980354196	1.31590021	Nabp1	nucleic acid binding protein 1 [Source:MGI Symbol;Acc:MGI:1923258]
Rab33b	370.273399	473.441803	-0.35	0.046604036	0.980354196	1.331576471	Rab33b	RAB33B, member RAS oncogene family [Source:MGI Symbol;Acc:MGI:1330805]
Axl	5986.9277	7625.08784	-0.35	0.020291641	0.980354196	1.69268283	Axl	AXL receptor tyrosine kinase [Source:MGI Symbol;Acc:MGI:1347244]
Mylip	1615.07619	2056.86806	-0.35	0.027939543	0.980354196	1.553780702	Mylip	myosin regulatory light chain interacting protein [Source:MGI Symbol;Acc:MGI:2388271]
Hcar2	232.514246	295.958766	-0.35	0.046808035	0.980354196	1.32967959	Hcar2	hydroxycarboxylic acid receptor 2 [Source:MGI Symbol;Acc:MGI:1933383]
Cyth4	3029.08044	3857.06142	-0.35	0.003518332	0.980354196	2.453663182	Cyth4	cytohesin 4 [Source:MGI Symbol;Acc:MGI:2441702]
Xcr1	365.944606	466.263222	-0.35	0.040794377	0.980354196	1.389399695	Xcr1	chemokine (C motif) receptor 1 [Source:MGI Symbol;Acc:MGI:1346338]
1700025G04Rik	303.732858	386.446856	-0.34	0.049637195	0.980354196	1.304192768	1700025G04Rik	RIKEN cDNA 1700025G04 gene [Source:MGI Symbol;Acc:MGI:1916649]
Galnt3	286.604852	362.826043	-0.34	0.040095088	0.980354196	1.396908829	Galnt3	polypeptide N-acetylgalactosaminyltransferase 3 [Source:MGI Symbol;Acc:MGI:894695]
Rcan3	368.632103	467.70731	-0.34	0.018098163	0.980354196	1.742365505	Rcan3	regulator of calcineurin 3 [Source:MGI Symbol;Acc:MGI:1858220]
Slc38a9	850.660085	1077.06926	-0.34	0.041392076	0.980354196	1.383082791	Slc38a9	solute carrier family 38, member 9 [Source:MGI Symbol;Acc:MGI:1918839]
Cd300a	1082.38417	1366.28136	-0.34	0.007738797	0.980354196	2.111326545	Cd300a	CD300A molecule [Source:MGI Symbol;Acc:MGI:2443411]
Shtn1	531.166679	670.802546	-0.34	0.019639372	0.980354196	1.706872404	Shtn1	shootin 1 [Source:MGI Symbol;Acc:MGI:1918903]
Selenop	5998.41779	7554.31104	-0.33	0.045939448	0.980354196	1.337814228	Selenop	selenoprotein P [Source:MGI Symbol;Acc:MGI:894288]
Slc35f5	177.813404	224.394481	-0.33	0.040926317	0.980354196	1.387997336	Slc35f5	solute carrier family 35, member F5 [Source:MGI Symbol;Acc:MGI:1921400]
Usp12	2133.97549	2685.67681	-0.33	0.041348381	0.980354196	1.383541491	Usp12	ubiquitin specific peptidase 12 [Source:MGI Symbol;Acc:MGI:1270128]
Star	1467.16002	1846.23395	-0.33	0.022003511	0.980354196	1.657508015	Star	steroidogenic acute regulatory protein [Source:MGI Symbol;Acc:MGI:102760]
Camk2n1	609.596611	766.489596	-0.33	0.009532758	0.980354196	2.020781432	Camk2n1	calcium/calmodulin-dependent protein kinase II inhibitor 1 [Source:MGI Symbol;Acc:MGI:1913509]
Etv6	1175.28866	1476.3155	-0.33	0.031766761	0.980354196	1.498027064	Etv6	ets variant 6 [Source:MGI Symbol;Acc:MGI:109336]
Ubash3b	597.4257	749.544668	-0.33	0.048002951	0.980354196	1.318732063	Ubash3b	ubiquitin associated and SH3 domain containing, B [Source:MGI Symbol;Acc:MGI:1920078]
Src	378.736526	474.952569	-0.33	0.047604382	0.980354196	1.322353068	Src	Rous sarcoma oncogene [Source:MGI Symbol;Acc:MGI:98397]
Gas2l1	507.463657	632.653086	-0.32	0.049464582	0.980354196	1.305705657	Gas2l1	growth arrest-specific 2 like 1 [Source:MGI Symbol;Acc:MGI:1926176]
4931406C07Rik	366.63685	455.519411	-0.31	0.045239027	0.980354196	1.344486744	4931406C07Rik	RIKEN cDNA 4931406C07 gene [Source:MGI Symbol;Acc:MGI:1918234]
Ppt1	3007.86448	3735.93309	-0.31	0.014261211	0.980354196	1.845843595	Ppt1	palmitoyl-protein thioesterase 1 [Source:MGI Symbol;Acc:MGI:1298204]
Scarb2	2542.99633	3153.80702	-0.31	0.022510205	0.980354196	1.64762055	Scarb2	scavenger receptor class B, member 2 [Source:MGI Symbol;Acc:MGI:1196458]
Acp2	1368.6021	1696.74745	-0.31	0.039958215	0.980354196	1.398393921	Acp2	acid phosphatase 2, lysosomal [Source:MGI Symbol;Acc:MGI:87882]
Dglucy	297.007823	368.542584	-0.31	0.047167326	0.980354196	1.326358744	Dglucy	D-glutamate cyclase [Source:MGI Symbol;Acc:MGI:2444813]
Inppl1	630.38919	781.32203	-0.31	0.034315194	0.980354196	1.464513541	Inppl1	inositol polyphosphate phosphatase-like 1 [Source:MGI Symbol;Acc:MGI:1333787]
Tcf7l2	359.228918	444.885374	-0.31	0.033179237	0.980354196	1.479133605	Tcf7l2	transcription factor 7 like 2, T cell specific, HMG box [Source:MGI Symbol;Acc:MGI:1202879]
Cd163	897.074922	1107.95175	-0.31	0.027501234	0.980354196	1.560647819	Cd163	CD163 antigen [Source:MGI Symbol;Acc:MGI:2135946]
Itgb5	1166.28247	1431.04086	-0.30	0.021322825	0.980354196	1.671155257	Itgb5	integrin beta 5 [Source:MGI Symbol;Acc:MGI:96614]
Col18a1	669.050961	821.57766	-0.30	0.044758497	0.980354196	1.349124506	Col18a1	collagen, type XVIII, alpha 1 [Source:MGI Symbol;Acc:MGI:88451]
Zcchc24	846.716228	1038.34295	-0.29	0.045623902	0.980354196	1.340807574	Zcchc24	zinc finger, CCHC domain containing 24 [Source:MGI Symbol;Acc:MGI:1919168]
Slc8b1	1243.10725	1520.11918	-0.29	0.027897463	0.980354196	1.55443529	Slc8b1	solute carrier family 8 (sodium/lithium/calcium exchanger), member B1 [Source:MGI Symbol;Acc:MGI:2180781]
Vamp4	591.494812	723.983638	-0.29	0.039451947	0.980354196	1.403931559	Vamp4	vesicle-associated membrane protein 4 [Source:MGI Symbol;Acc:MGI:1858730]
Igf1	1198.4577	1462.41373	-0.29	0.022715662	0.980354196	1.643674602	Igf1	insulin-like growth factor 1 [Source:MGI Symbol;Acc:MGI:96432]
BC035044	595.542608	724.02049	-0.28	0.009719675	0.980354196	2.012348256	BC035044	cDNA sequence BC035044 [Source:MGI Symbol;Acc:MGI:2448540]
Klhl18	871.948531	1058.23277	-0.28	0.043160679	0.980354196	1.364911732	Klhl18	kelch-like 18 [Source:MGI Symbol;Acc:MGI:2143315]
Crk	1567.99682	1901.34231	-0.28	0.045036434	0.980354196	1.346436004	Crk	v-crk avian sarcoma virus CT10 oncogene homolog [Source:MGI Symbol;Acc:MGI:88508]
Sh3bp2	1194.96736	1449.60019	-0.28	0.024669707	0.980354196	1.607836009	Sh3bp2	SH3-domain binding protein 2 [Source:MGI Symbol;Acc:MGI:1346349]
Neurl3	2301.82483	2790.068	-0.28	0.003683943	0.980354196	2.433687098	Neurl3	neuralized E3 ubiquitin protein ligase 3 [Source:MGI Symbol;Acc:MGI:2429944]
Cadm1	1035.25302	1251.57537	-0.27	0.04620577	0.980354196	1.335303788	Cadm1	cell adhesion molecule 1 [Source:MGI Symbol;Acc:MGI:1889272]
Pla2g15	1422.72653	1719.00665	-0.27	0.028130627	0.980354196	1.550820588	Pla2g15	phospholipase A2, group XV [Source:MGI Symbol;Acc:MGI:2178076]
Zfp385a	1063.46412	1283.75941	-0.27	0.035468896	0.980354196	1.450152329	Zfp385a	zinc finger protein 385A [Source:MGI Symbol;Acc:MGI:1352495]
Stam2	494.900266	597.384315	-0.27	0.027342651	0.980354196	1.563159381	Stam2	signal transducing adaptor molecule (SH3 domain and ITAM motif) 2 [Source:MGI Symbol;Acc:MGI:1929100]
Hdac5	1333.14279	1605.97656	-0.27	0.039359569	0.980354196	1.404949666	Hdac5	histone deacetylase 5 [Source:MGI Symbol;Acc:MGI:1333784]
Fbxo18	810.410559	975.700614	-0.27	0.026556947	0.980354196	1.575821853	Fbxo18	F-box protein 18 [Source:MGI Symbol;Acc:MGI:1354699]
Eml3	1367.66863	1645.46246	-0.27	0.032549804	0.980354196	1.487451622	Eml3	echinoderm microtubule associated protein like 3 [Source:MGI Symbol;Acc:MGI:2387612]
Trp53inp2	592.215966	709.158664	-0.26	0.040335293	0.980354196	1.394314784	Trp53inp2	transformation related protein 53 inducible nuclear protein 2 [Source:MGI Symbol;Acc:MGI:1915978]
Mpzl1	378.451885	452.519666	-0.26	0.040262957	0.980354196	1.395094333	Mpzl1	myelin protein zero-like 1 [Source:MGI Symbol;Acc:MGI:1915731]
Vhl	625.470905	746.80275	-0.25	0.026839353	0.980354196	1.571227958	Vhl	von Hippel-Lindau tumor suppressor [Source:MGI Symbol;Acc:MGI:103223]
Wdr45b	543.714576	647.750296	-0.25	0.02550107	0.980354196	1.593441597	Wdr45b	WD repeat domain 45B [Source:MGI Symbol;Acc:MGI:1914090]
Itgb3	1415.22643	1670.45735	-0.24	0.046791458	0.980354196	1.329833422	Itgb3	integrin beta 3 [Source:MGI Symbol;Acc:MGI:96612]
Snx25	728.272515	859.770967	-0.24	0.035821618	0.980354196	1.445854802	Snx25	sorting nexin 25 [Source:MGI Symbol;Acc:MGI:2142610]
Skap2	1518.22301	1788.92318	-0.24	0.017974279	0.980354196	1.745348521	Skap2	src family associated phosphoprotein 2 [Source:MGI Symbol;Acc:MGI:1889206]
Strip1	983.754822	1158.78156	-0.24	0.010827113	0.980354196	1.965487331	Strip1	striatin interacting protein 1 [Source:MGI Symbol;Acc:MGI:2443884]
Ubl3	1768.47818	2032.4704	-0.20	0.042584638	0.980354196	1.37074704	Ubl3	ubiquitin-like 3 [Source:MGI Symbol;Acc:MGI:1344373]
Asb2	2507.52285	2860.335	-0.19	0.046494199	0.980354196	1.33260123	Asb2	ankyrin repeat and SOCS box-containing 2 [Source:MGI Symbol;Acc:MGI:1929743]

** rows 4-455 = genes with increased expression in Mg2800; rows 457-1110 = genes with decreased expression in Mg2800 synovial tissues, compared with Mg500.*
